# MNK-driven eIF4E phosphorylation regulates the fibrogenic transformation of mesenchymal cells and chronic lung allograft dysfunction

**DOI:** 10.1172/JCI168393

**Published:** 2024-08-15

**Authors:** Natalie M. Walker, Yuta Ibuki, A. Patrick McLinden, Keizo Misumi, Dylan C. Mitchell, Gabriel G. Kleer, Alison M. Lock, Ragini Vittal, Nahum Sonenberg, Amanda L. Garner, Vibha N. Lama

**Affiliations:** 1 Department of Internal Medicine, University of Michigan, Ann Arbor, Michigan, USA.; 2 Division of Pulmonary, Allergy, Critical Care, and Sleep Medicine, Department of Medicine, Emory University School of Medicine, Atlanta, Georgia, USA.; 3 Department of Medicinal Chemistry, University of Michigan, Ann Arbor, Michigan, USA.; 4 Department of Biochemistry and McGill Cancer Center, McGill University, Montreal, Quebec, Canada.

**Keywords:** Pulmonology, Transplantation, Cell migration/adhesion, Extracellular matrix, Translation

## Abstract

Tissue fibrosis remains unamenable to meaningful therapeutic interventions and is the primary cause of chronic graft failure after organ transplantation. Eukaryotic translation initiation factor (eIF4E), a key translational regulator, serves as convergent target of multiple upstream profibrotic signaling pathways that contribute to mesenchymal cell (MC) activation. Here, we investigate the role of MAP kinase–interacting serine/threonine kinase–induced (MNK-induced) direct phosphorylation of eIF4E at serine 209 (Ser209) in maintaining fibrotic transformation of MCs and determine the contribution of the MNK/eIF4E pathway to the pathogenesis of chronic lung allograft dysfunction (CLAD). MCs from patients with CLAD demonstrated constitutively higher eIF4E phosphorylation at Ser209, and eIF4E phospho-Ser209 was found to be critical in regulating key fibrogenic protein autotaxin, leading to sustained β-catenin activation and profibrotic functions of CLAD MCs. MNK1 signaling was upregulated in CLAD MCs, and genetic or pharmacologic targeting of MNK1 activity inhibited eIF4E phospho-Ser209 and profibrotic functions of CLAD MCs in vitro. Treatment with an MNK1/2 inhibitor (eFT-508) abrogated allograft fibrosis in an orthotopic murine lung-transplant model. Together these studies identify what we believe is a previously unrecognized MNK/eIF4E/ATX/β-catenin signaling pathway of fibrotic transformation of MCs and present the first evidence, to our knowledge, for the utility of MNK inhibitors in fibrosis.

## Abstract 2

MNK/eIF4E-Ser209 axis plays a key role in the fibrogenic transformation of mesenchymal cells and is a promising therapeutic target in fibrosis.

## Graphical abstract

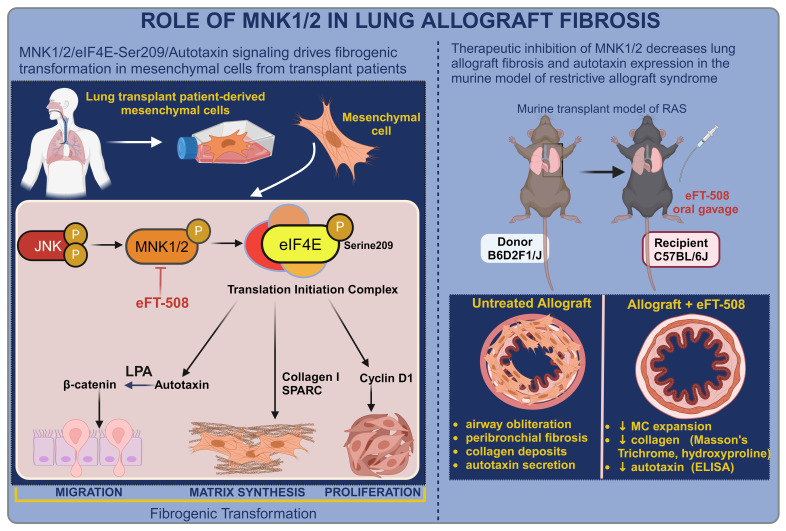

## Introduction

Lung transplantation has the worst long-term prognosis among all solid-organ transplantations, with a 10-year survival of only 20% (1). The major cause of these poor long-term outcomes is progressive irreversible decline in lung function secondary to fibrotic allograft remodeling termed chronic lung allograft dysfunction (CLAD) (2). Two predominant clinical phenotypes of CLAD distinguished by obstructive or restrictive physiology are recognized and termed bronchiolitis obliterans syndrome (BOS) and restrictive allograft syndrome (RAS), respectively. Fibrosis is a key feature of both, with small airway fibrotic obliteration marking BOS and more diffuse and robust fibrosis infiltrating along the bronchovascular bundles and pleura noted in RAS. Progressive tissue fibrosis, unamenable to therapeutic interventions, is also a hallmark of various chronic and often fatal diseases across multiple organs. While often initiated by alloimmune or autoimmune tissue injury, fibrosis is recalcitrant to immunomodulation and mesenchymal cells (MCs) isolated from fibrotic tissues demonstrate cell-autonomous behavior (3–7). Targeting mechanisms of fibrotic transformation of MCs is key to therapeutic targeting of these fibrotic diseases.

Our prior work identified donor-derived mesenchymal stromal cells, marked by their expression of embryonic mesenchymal transcriptional factor forkhead box F1 (Foxf1), in bronchoalveolar lavage (BAL) fluid from human lung allografts (8, 9). These transcriptionally distinct subsets of lung-resident MCs were found to reside along the bronchovascular bundles and play a key role in the pathogenesis of CLAD (10). A persistently activated phenotype marked by increased matrix deposition and stable induction of multiple profibrotic pathways was identified in MCs isolated from patients with CLAD (3, 8, 9, 11, 12). Among factors found to sustain the fibrotic phenotype of MCs was the acquisition of an autocrine signaling program driven by increased expression of secreted lysophospholipase, autotaxin (ATX) (3, 7). Bioactive lipid lysophosphatidic acid (LPA), generated through the actions of secreted ATX, promoted downstream transcriptional activation via β-catenin stabilization (3, 13) and translational activation via signaling to mTOR (11, 12). These profibrotic pathways were shown to converge at the level of eukaryotic translation initiation factor–driven (eIF4E-driven) cap-dependent translation to regulate key fibrotic functions such as collagen I expression in CLAD MCs (11).

The translation initiation factor eIF4E is described as a proto-oncogene secondary to its key role in promoting cellular transformation specifically through the selective translation of mRNAs key to driving processes, such as cellular growth, proliferation, migration, and survival (14–18). Key to the oncogenic transforming potential of eIF4E is its phosphorylation on serine 209 (Ser209), its only known site of phosphorylation (14, 16, 19–21). The requisite role of phosphorylation of eIF4E at Ser209 in cellular activation and oncogenesis has been established in studies of *Eif4e^Ser209A/A^* mutant mice expressing a nonphosphorylatable form of eIF4E (14). These studies suggest that among the mRNAs that preferentially require eIF4E phosphorylation for maximal translation are those that encode proteins involved in extracellular matrix remodeling and paracrine signaling (14). Protein kinases MAP kinase–interacting serine/threonine kinase 1 and 2 (MNK1/2) are exclusively implicated in the phosphorylation of eIF4E at Ser209, and targeting the MNK/eIF4E axis via MNK inhibitors is being actively explored as a therapeutic option in the field of oncology (15, 19, 22–27). However, the role of the MNK/eIF4E axis in regulating MC functions and fibrogenesis remains to be elucidated.

Here, we establish a critical role for eIF4E Ser209 phosphorylation in regulating a key fibrogenic protein ATX and sustaining the fibrotic functions of MCs. We demonstrate that fibrotic MCs are reliant on the MNK/eIF4E axis for functions such as migration and matrix production and that interrupting this axis abrogates allograft fibrosis in an orthotopic murine lung-transplant model. Selectively targeting the increased translational efficiency of fibrotic cells driven by eIF4E Ser209 phosphorylation could represent an effective strategy for halting progressive fibrosis.

## Results

### mTORC1/2 independent, MNK-eIF4E Ser209 phosphorylation in fibrotic MCs.

**Figure 1:**
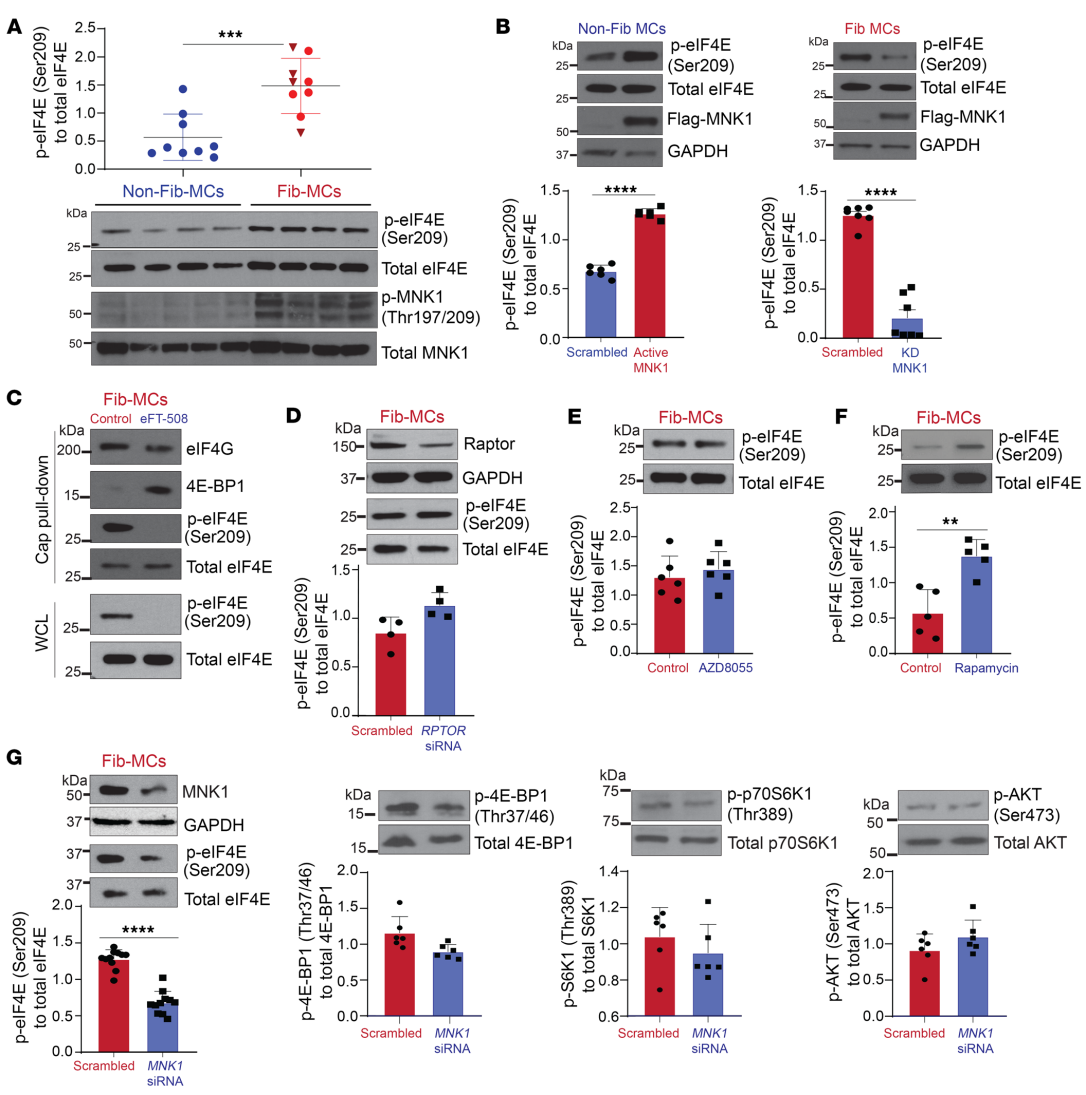
Phosphorylation of eIF4E (Ser209) is MNK1 dependent but mTORC-independent in fibrotic MCs derived from lung-transplant patients. (**A**) Protein expression of phosphorylated and total forms of eIF4E and MNK1 was measured by Western blot analysis in MCs derived from normal (non-Fib-MCs) or fibrotic (Fib-MCs) human-lung allografts. Densitometry analyses of phospho-eIF4E to total eIF4E are shown with Fib-MCs for RAS (circles) and BOS (triangles). (**B**) Lentiviral infections were performed using empty pLenti-LoxEV vector or vector expressing active MNK1 (*T344D*) in nonfibrotic MCs or kinase-dead MNK1 (*D191A*) in Fib-MCs. Western blotting and corresponding densitometry analyses were performed for phospho-eIF4E and total eIF4E. (**C**) Fibrotic MCs treated with eFT-508 (10 μM, 24 hours) were subjected to m^7^GDP cap pulldown assay followed by Western blotting analyses. (**D**) Fib-MCs were transfected with *RPTOR*-specific or scrambled siRNA, and protein lysates were analyzed by Western blotting. Representative immunoblots and corresponding densitometry are shown for phospho-eIF4E and total eIF4E. (**E** and **F**) Fib-MCs were treated with ATP-competitive mTORC inhibitors (AZD8055: 250 nM; rapamycin: 250 nM) for 24 hours and analyzed for phospho-eIF4E and total eIF4E by Western blotting and densitometry. (**G**) Fib-MCs were transfected with *MNK1*-specific or scrambled siRNA. Protein lysates were immunoblotted for mTORC1/2 substrates. Representative immunoblots and corresponding densitometry are shown for phosphorylated and total forms of 4E-BP1, p70S6K1, and AKT. Data are represented as means ± SEM. **P < 0.01; ***P < 0.001; ****P < 0.0001, unpaired *t* test.

eIF4E activity is regulated by 2 major signaling pathways, mTOR and MAPK, both of which play critical roles in regulating cellular functions (27–29). Our previous reports demonstrate that PI3K/mTOR pathways are activated in MCs in CLAD (11, 12). To determine whether eIF4E phosphorylation at Ser209 is also dysregulated in CLAD, we compared protein phosphorylated eIF4E (phospho-eIF4E) (Ser209) expression in MCs from lung-transplant patients with (*n* = 9) or without CLAD (*n* = 9) (Figure 1A). Each sample represents MCs derived from the BAL fluid of an individual patient, and clinical variables for the patients and BAL samples are presented in Table 1. Whole-cell lysates of MCs derived from patients with CLAD (Fib-MCs) had significantly higher eIF4E (Ser209) protein expression as compared with MCs derived from control, CLAD-free patients (non-Fib-MCs) (*P* = 0.006). CLAD samples included both predominant phenotypes of RAS and BOS; no significant difference was noted in eIF4E (Ser209) phosphorylation between RAS and BOS MCs (*P* = 0.59).

To ascertain that MNK is the primary kinase that phosphorylates eIF4E in human lung MCs, plasmids expressing flag-tagged MNK1 were utilized to overexpress constitutively active or kinase-dead MNK1 in non-Fib and Fib-MCs, respectively. As seen in Figure 1B, non-Fib-MCs expressing constitutively active MNK1 demonstrated significantly higher levels of phospho-eIF4E Ser209 compared with scrambled control. Fib-MCs demonstrated higher MNK1 phosphorylation (Figure 1A), and expression of kinase-dead MNK1 resulted in significantly decreased eIF4E (Ser209) phosphorylation in these cells (Figure 1B). To confirm the role of MNK-driven eIF4E (Ser209) phosphorylation in modulating cap-dependent translation, a cap pulldown assay was utilized to assess the amount of eIF4E (Ser209) on the cap-initiation complex and its response to MNK inhibition. As seen in Figure 1C, phospho-eIF4E (Ser209) was found to be highly associated with eIF4G at baseline in Fib-MCs, suggesting phosphorylation is present during active cap translation. Upon MNK1/2 inhibition with eFT-508, eIF4E (Ser209) phosphorylation was almost ablated and 4E-BP1 was reassociated with eIF4E, demonstrating that MNK inhibition is effective at halting cap translation in Fib-MCs.

Next, we investigated whether increased eIF4E (Ser209) phosphorylation noted in Fib-MCs depends on mTOR activation. Neither silencing of mTORC1 critical component *RPTOR* nor treatment with combined mTORC1 and mTORC2 inhibitor AZD8055 (30) resulted in any change in eIF4E phosphorylation in Fib-MCs (Figure 1, D and E). Consistent with what has been previously reported in other cell types (15, 19, 31), rapamycin treatment of Fib-MCs led to increased phosphorylation of eIF4E (Ser209) (Figure 1F). As a previous report implicates a role for MNK-driven mTORC1 activation (31), *MNK1* silencing was performed in fibrotic MCs and mTORC substrate phosphorylation was assessed. No significant reduction in 4E-BP1 (Thr37/46), p70S6 kinase (Thr389), or AKT (Ser473) phosphorylation was noted in response to *MNK1* silencing (Figure 1G). Together, these data suggest that mTORC1/2 and MNK/eIF4E represent 2 separately activated pathways in lung MCs and an mTORC-independent eIF4E phosphorylation is noted in CLAD MCs.

**Figure 2:**
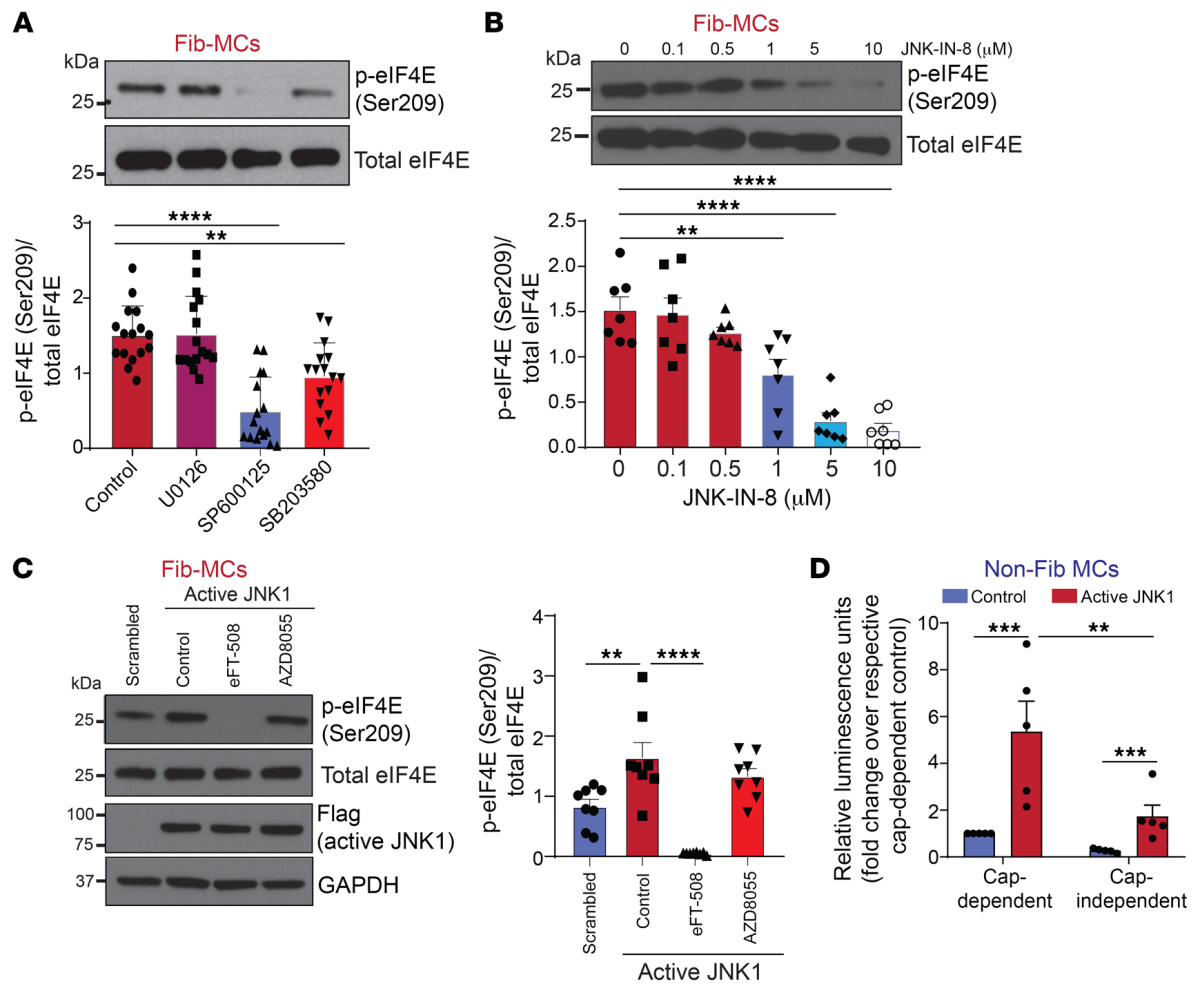
MNK/eIF4E (Ser209) activation is mediated by upstream JNK signaling. (**A**–**C**) MCs derived from fibrotic human lung allografts (Fib-MCs) were treated with pharmacologic inhibitors against MEK1/2 (U0126), JNK (broad spectrum effect — SP600125), and p38 MAPK (SB203580) at 10 μM for 2 hours (**A**), treated with the indicated doses of irreversible JNK1/2/3 inhibitor (JNK-IN-8) (**B**, 2 hours), or subjected to lentiviral infections of empty pLenti-LoxEV vector or vector expressing constitutively active JNK1, followed by treatment with eFT-508 (**C**; 10 μM, 2 hours) or AZD8055 (**C**; 250 nM, 2 hours). Protein lysates were analyzed by Western blotting. Representative immunoblots and corresponding densitometry are shown for phospho-eIF4E and total eIF4E. (**D**) MCs derived from normal human lung allografts (non-Fib-MCs) were infected with lentiviral particles containing constitutively active JNK1 for 48 hours, followed by infection with particles containing rLuc-PolIRES-fLuc cap-translation luciferase vector. Twenty-four hours later, lysates were collected and readings for Renilla and Firefly luminescence were performed. Data are represented as means ± SEM. **P < 0.01; ***P < 0.001; ****P < 0.0001, 1-way ANOVA; post hoc test: Bonferroni’s test (**A**–**C**); 2-way ANOVA; post hoc test: Bonferroni’s test (**D**).

To further investigate the upstream signaling contributing to constitutive phosphorylation of eIF4E in fibrotic MCs, human lung allograft-derived Fib-MCs were treated with MAPK inhibitors U0126, SP600125, and SB203580 targeting MEK/ERK, JNK, and p38MAPK, respectively. Interestingly, JNK inhibition resulted in the most robust decrease in eIF4E phosphorylation in these fibrotic MCs (Figure 2A). This surprising role of JNK in regulating constitutive phosphorylation of eIF4E in fibrotic MCs was further confirmed by utilizing JNK-IN-8, a more selective JNK inhibitor (32), which also significantly decreased eIF4E phosphorylation in a dose-dependent manner (Figure 2B). To determine whether JNK activity is sufficient to drive eIF4E phosphorylation, we induced the expression of a constitutively active form of JNK1 in nonfibrotic MCs as previously described (12). A significant increase in eIF4E phosphorylation, sensitive to MNK1 but not mTORC inhibition, was noted in non-Fib-MCs with constitutive activation of JNK1 (Figure 2C). Next, utilizing MCs expressing a bicistronic reporter plasmid by which translation of renilla luciferase occurs via cap-dependent translation and firefly luciferase directed by a polio internal ribosome entry site measures cap-independent translation (12) (33), active JNK1 expression was noted to induce an increase in cap-dependent translation (Figure 2D).

### MNK/eIF4E Ser209 signaling axis drives the profibrotic function of MCs.

**Figure 3:**
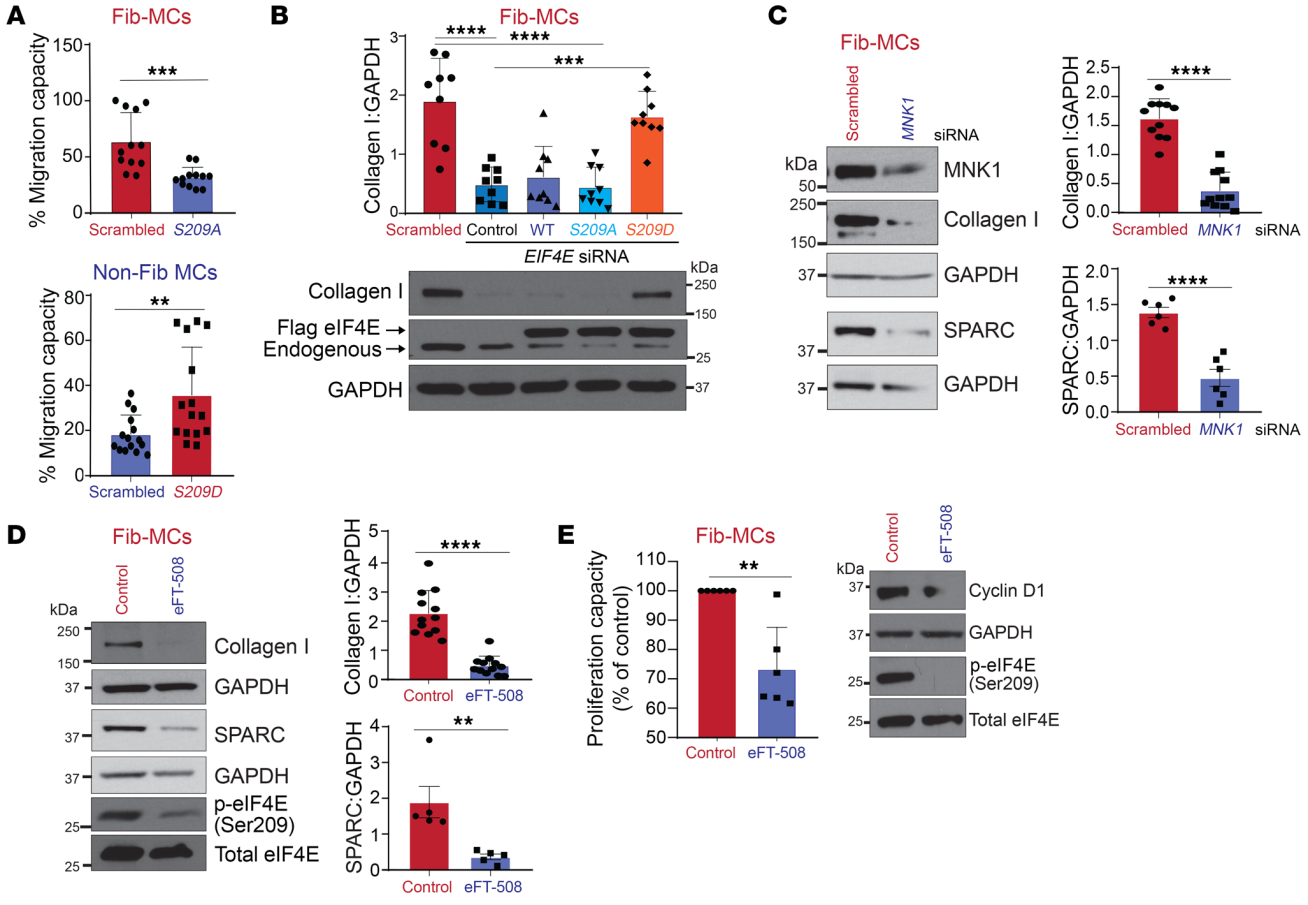
MNK/eIF4E (Ser209) activation drives profibrotic phenotypes in lung MCs. (**A**) MCs isolated from fibrotic (Fib-MCs) and normal human lung allografts (non-Fib-MCs) were infected with lentiviral particles containing pLenti-LoxEV–eIF4E (*S209A*) and pLenti-LoxEV–eIF4E (*S209D*), respectively, and then cultured in Matrigel-coated Transwells. Cell migration was assessed by MTT assay. (**B**) Fib-MCs were transfected with *EIF4E* or scrambled control siRNA followed by lentiviral infection of empty vector or vector expressing *S209A* or *S209D*. Protein lysates were immunoblotted against collagen I. Representative immunoblots and corresponding densitometry are shown. (**C** and **D**) Fib-MCs were transfected with scrambled or *MNK1* siRNA (**C**) or treated with eFT-508 (10 μM, 2 hours) (**D**). Protein lysates were immunoblotted against collagen I and SPARC. Representative immunoblots and corresponding densitometry are shown. (**E**) Proliferation was assessed in Fib-MCs treated with eFT-508 (10 μM, 72 hours) using Cyquant Proliferation Assay Kit. Protein lysates were immunoblotted for cyclin D1 as well as phosphorylated and total eIF4E. Data are represented as means ± SEM. **P < 0.01; ***P < 0.001; ****P < 0.0001, unpaired *t* test (**A**, **C**, **D**, and **E**); 1-way ANOVA; post hoc test: Bonferroni’s test (**B**).

To investigate the role of eIF4E Ser209 phosphorylation in regulating fibrotic functions of MCs, we infected Fib-MCs with a lentiviral vector containing the nonphosphorylatable eIF4E mutant, harboring a serine-to-alanine mutation (*S209A*). As seen in Figure 3A, infection of Fib-MCs with *S209A* lentiviral vector significantly decreased MC migration in a modified Boyden chamber Transwell assay (13). Conversely, expression of a constitutively phosphorylated mutant with a serine–to–aspartic acid mutation (*S209D*) in non-Fib-MCs resulted in increased migration capacity (Figure 3A). To investigate the role of eIF4E (Ser209) phosphorylation in regulating matrix protein expression, Fib-MCs were transfected with nontargeting control or *EIF4E*-specific siRNA and collagen I protein expression assessed by immunoblotting. As seen in Figure 3B, *EIF4E* silencing significantly inhibited collagen I expression in Fib-MCs. To subsequently determine whether eIF4E phosphorylation is required to drive collagen I expression, MCs were silenced with siRNA for *EIF4E*, followed by infection with lentiviral vectors containing WT, *S209A*, or *S209D* mutants of eIF4E. Only MCs reintroduced with the constitutively phospho-eIF4E *S209D* mutant exhibited a rescue in collagen I expression, supporting the notion that in Fib-MCs, phosphorylation of eIF4E (Ser209) is required for driving collagen I expression. The effect of MNK inhibition on collagen I expression was also studied by genetic and pharmacologic approaches. *MNK1* silencing resulted in a 90% decrease in collagen I expression in Fib-MCs along with significantly blunted expression of SPARC, a marker of matrix remodeling (Figure 3C). Pharmacologic inhibition of MNK1/2 utilizing eFT-508 significantly decreased collagen I and SPARC expression in Fib-MCs (Figure 3D) and was also noted to decrease MC proliferation and cyclin D1 expression (Figure 3E).

### eIF4E Ser209 phosphorylation as a translational regulator of key fibrogenic factor ATX in lung MCs.

**Figure 4:**
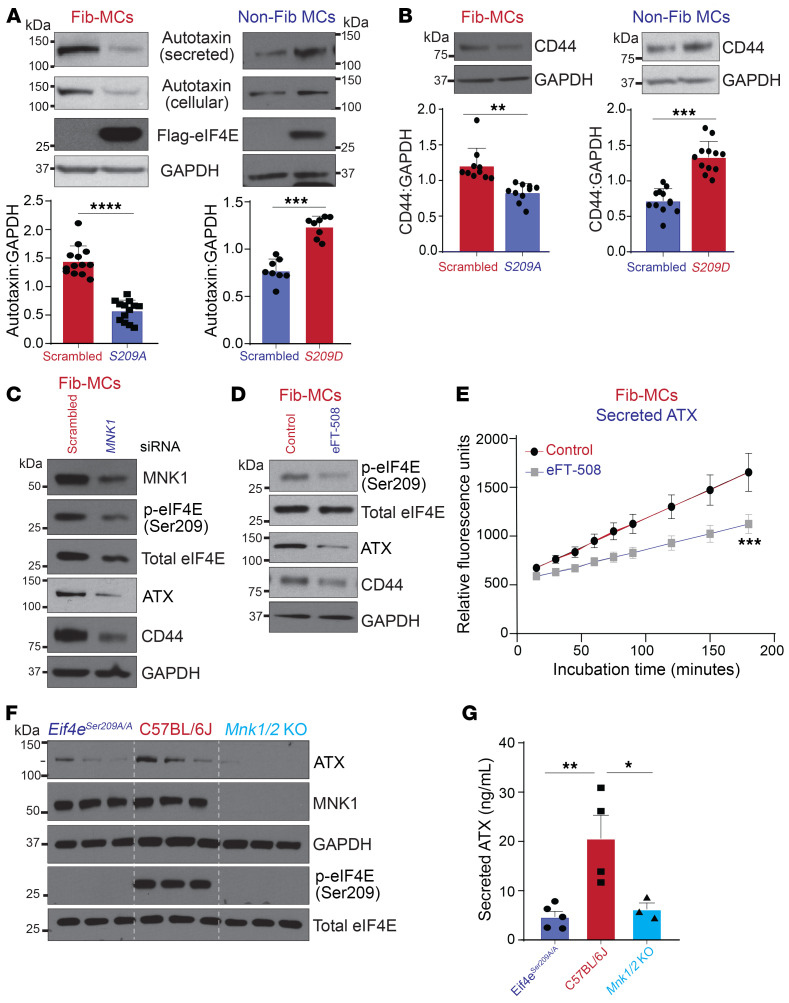
MNK/eIF4E (Ser209) positively regulates ATX expression and activity in fibrotic lung MCs. (**A**) MCs isolated from fibrotic (Fib-MCs) or normal (non-Fib-MCs) human lung allografts were infected with lentiviral vectors containing pLenti-LoxEV–eIF4E (*S209A*) and pLenti-LoxEV–eIF4E (*S209D*) plasmids, respectively. Protein lysates were analyzed by Western blotting for cellular and secreted ATX expression. Representative immunoblots and densitometry for cellular ATX are shown. (**B**) Immunoblots for CD44 protein expression in lentiviral-infected MCs described in **A**. (**C** and **D**) Fib-MCs were transfected with *MNK1* or scrambled siRNA (**C**) or treated with eFT-508 (10 μM, 2 hours; **D**). Protein lysates were analyzed by Western blotting. Representative immunoblots of phospho-eIF4E and total forms of eIF4E, and expression of cellular ATX and CD44 are shown. (**E**) Conditioned media of Fib-MCs treated with eFT-508 (10 μM, 24 hours) were measured for ATX activity utilizing the fluorogenic substrate FS-3. *n* = 9. (**F**) Cultured mouse lung fibroblasts from WT, *Eif4e^Ser209A/A^,* and *Mnk1/2*-KO mice were assessed by immunoblotting for cellular ATX, MNK1, phospho-eIF4E, and total eIF4E. (**G**) Secreted ATX levels were analyzed in the conditioned media by sandwich ELISA. Data are represented as means ± SEM. *P < 0.05; **P < 0.01; ***P < 0.001; ****P < 0.0001, unpaired t test (**A** and **B**); simple linear regression (**E**); 1-way ANOVA; post hoc test: Bonferroni’s test (**G**).

We have previously demonstrated that human lung–derived MCs secrete ATX and that ATX expression is significantly increased in Fib-MCs (3). This autocrine ATX generates LPA, and subsequent downstream LPA1 signaling can regulate both MC migration and collagen I expression (3, 13). However, despite the recognition of the substantial role of ATX in fibrotic diseases, regulation of ATX expression in MCs is not well understood. Interestingly, the ATX gene *ENPP2* was a top hit among mRNAs significantly downregulated in polysome profiling utilized to sequence the specific mRNAs engaged on actively translating ribosomes between immortalized embryonic fibroblasts derived from WT mice and *Eif4e^Ser209A/A^* mice (14). *Eif4e^Ser209A/A^* mice developed in house have a serine to alanine mutation that renders eIF4E nonphosphorylatable (14). To investigate whether eIF4E (Ser209) regulates ATX expression in lung MCs, infection of Fib-MCs with *S209A* lentiviral vector was first utilized. As shown in Figure 4A, significantly decreased expression of secreted and cellular ATX was noted. Conversely, expression of a constitutively phosphorylated mutant *S209D* in non-Fib-MCs resulted in elevated ATX cellular and secreted levels. Expression of CD44, a cell-surface protein with reported migratory (6, 34) and profibrotic functions (6, 35), in MCs was also significantly abrogated in Fib-MCs upon *S209A* expression, while non-Fib-MCs expressing *S209D* eIF4E mutant demonstrated increased expression of CD44 (Figure 4B). We next investigated the role of MNK1 in regulating ATX expression of MCs. Fib-MCs demonstrated decreased ATX and CD44 expression upon *MNK1* silencing (Figure 4C). MNK1/2 inhibition utilizing the pharmacological inhibitor eFT-508 resulted in a significant reduction in both expression and activity of ATX, along with reduced expression of CD44 (Figure 4, D and E), with no significant reduction in *ATX* mRNA expression noted (data not shown). This pathway was further validated by utilizing MCs from lungs of WT, *Eif4e^Ser209A/A^*, and *Mnk1/2* double-knockout mice. MCs isolated from lungs of *Eif4e^Ser209A/A^* and *Mnk1/2*-KO mice demonstrated no detectable eIF4E Ser209 phosphorylation when compared with MCs from WT lungs. Lower expression of ATX protein was noted in cell homogenate (Figure 4F) and supernatant (Figure 4G) in *Eif4e^Ser209A/A^* and *Mnk1/2*-KO lung MCs.

### MNK1/eIF4E Ser209-driven ATX expression drives β-catenin expression in fibrotic MCs.

**Figure 5:**
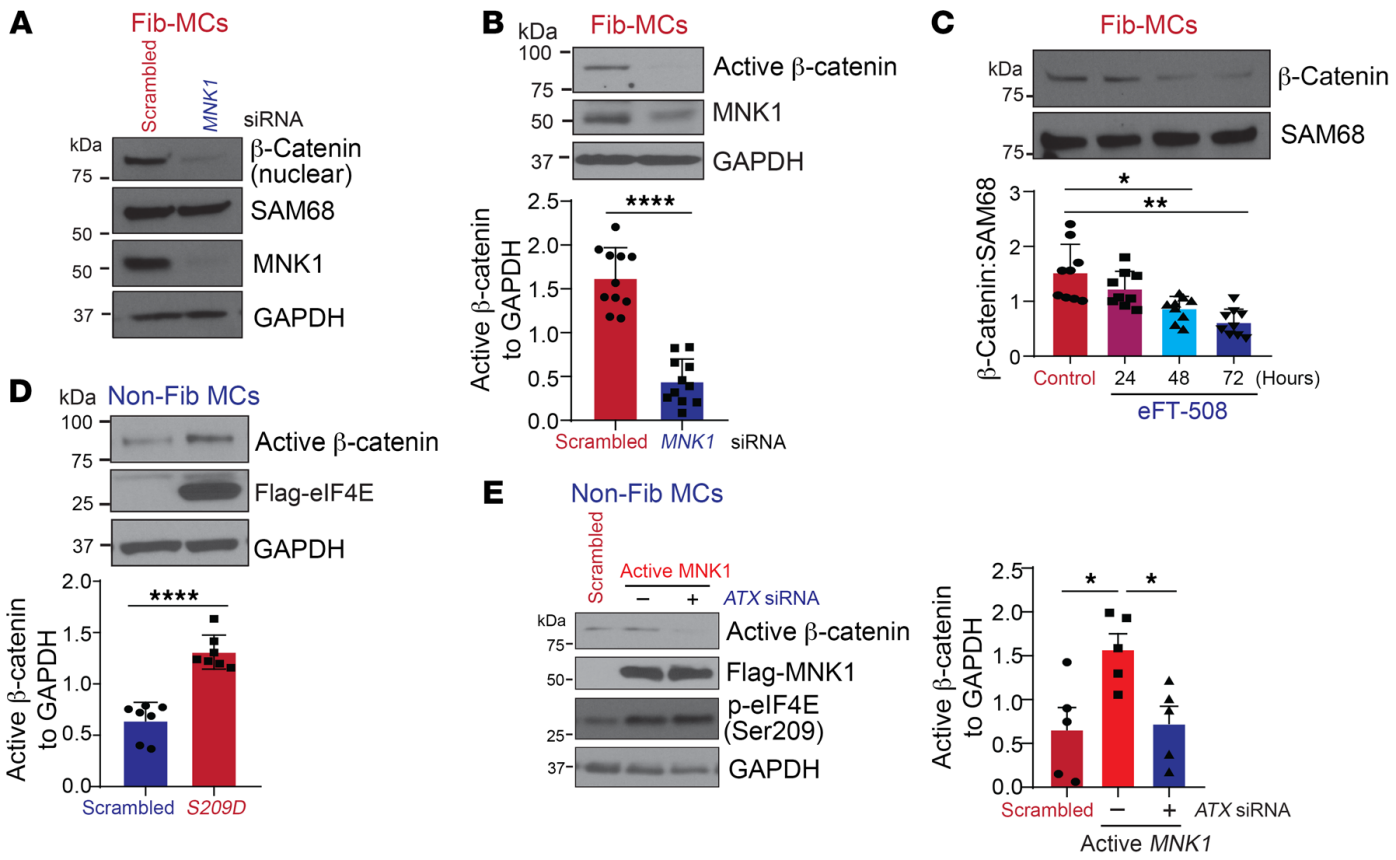
MNK/eIF4E (Ser209)/ATX signaling drives β-catenin expression in lung MCs. (**A** and **B**) MCs derived from fibrotic human-lung allografts (Fib-MCs) were transfected with *MNK1* or scrambled control siRNA. (**A**) Nuclear extracts were analyzed by immunoblotting for β-catenin expression. Representative image from 6 cell lines. (**B**) Whole-cell lysates were assessed for active, nonphosphorylated β-catenin by immunoblotting and densitometry. (**C**) Fib-MCs were treated with eFT-508 (10 μM). Nuclear extracts were assessed for β-catenin expression by immunoblotting and densitometry. (**D**) Nonfibrotic MCs (non-Fib-MCs) were subjected to lentiviral infection with pLenti-LoxEV–eIF4E *S209D* plasmid. Protein lysates were assessed for active β-catenin expression. (**E**) Non-Fib-MCs were silenced with scrambled or *ATX* siRNA followed by lentiviral expression of active mutant pLenti-LoxEV–MNK1 (*T344D*). Western blotting analyses were performed for active β-catenin, MNK1, and phospho-eIF4E. Data are represented as means ± SEM. *P < 0.05; **P < 0.01; ****P < 0.0001, unpaired *t* test (**B** and **D**); 1-way ANOVA; post hoc test: Bonferroni’s test (**C** and **E**).

MCs derived from fibrotic lung allografts demonstrate constitutive nuclear β-catenin expression, which has been shown to be dependent on autocrine ATX secretion and LPA1 signaling (3). Secondary to the ability of MNK/eIF4E Ser209 to regulate protein expression of ATX, we investigated to determine whether β-catenin is regulated via this pathway in Fib-MCs. In Fib-MCs, *MNK1* siRNA–mediated silencing led to a 3-fold decrease in nuclear β-catenin levels (Figure 5A). Additionally, the nonphosphorylated active form of β-catenin was significantly decreased in lysates of *MNK1*-silenced MCs (Figure 5B). Time-course treatment with MNK inhibitor eFT-508 led to a significant decrease in nuclear β-catenin expression at 48 and 72 hours after treatment in fib-MCs (Figure 5C). Non-Fib-MCs expressing constitutively active *S209D* eIF4E mutant demonstrated an over 2-fold induction in the expression of active β-catenin (Figure 5D). Finally, to determine the requirement of ATX expression in MNK/eIF4E-induced β-catenin activation, non-Fib-MCs expressing constitutively active MNK1 were silenced for *ATX* and immunoblotted for active β-catenin protein expression. As seen in Figure 5E, *ATX* silencing significantly reduced MNK1-driven active β-catenin expression in non-Fib-MCs.

### MNK1/eIF4E Ser209 drives ATX expression in vivo and MNK inhibition attenuates fibrosis in a murine orthotopic lung-transplant model of chronic rejection.

**Figure 6:**
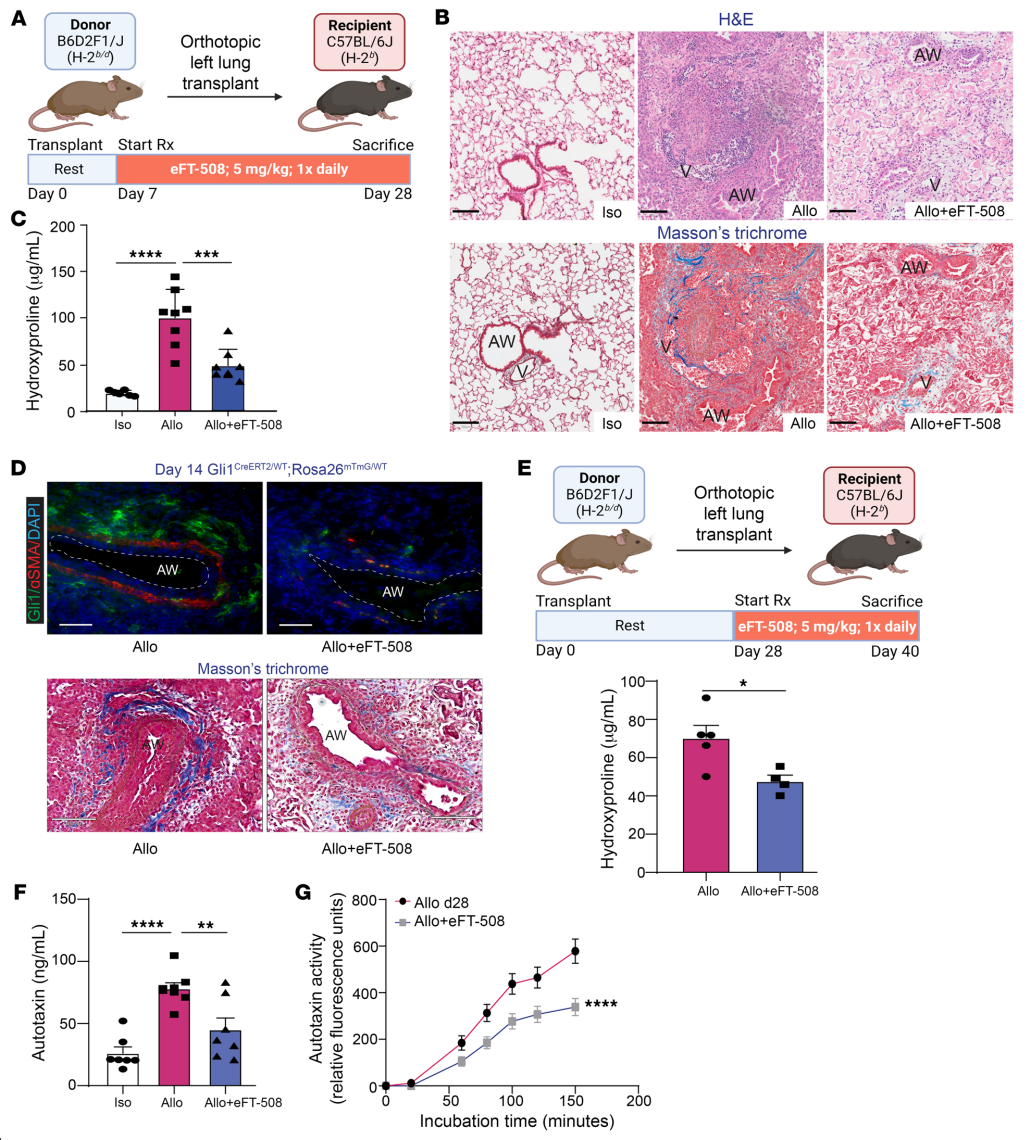
Pharmacologic MNK inhibition utilizing eFT-508 decreases lung allograft fibrosis and ATX expression in a murine orthotopic left lung–transplant model of RAS. (**A**) Experimental schematic. B6D2F1/J donor lungs were transplanted into C57BL/6J recipient mice, followed by treatment with eFT-508 (5 mg/kg/d; oral gavage) between days 7 and 28. (**B**) Histopathology examination of formalin-fixed, paraffin-embedded tissues — isografts (Iso), RAS allografts (Allo), and eFT-508-treated RAS allografts (Allo+eFT-508) at day 28 using H&E staining (top panels) and Masson’s trichrome staining (bottom panels). Scale bars: 100 μm. V, vessel; AW, airway. (**C**) Acid-digested lung homogenates were assessed for collagen content by hydroxyproline assay. *n* = 6–8. Data are represented as means ± SEM. ***P < 0.001; ****P < 0.0001, 1-way ANOVA; post hoc test: Bonferroni’s test. (**D**) Gli1^CreERT2/WT^;Rosa26^mTmG/WT^ murine allograft controls or treated with eFT-508 were harvested at day 14 after transplant. Top panels: dual-labeling against GFP (green) and α-smooth muscle actin (α-SMA) (red). Nuclear counterstaining was by DAPI. Original magnification, ×400. Scale bars: 50 μm. Bottom panels: Masson’s trichrome staining in contiguous sections indicating collagen deposition (blue). (**E**) eFT-508 therapeutic treatment regimen from days 28 to 40 after transplant resulted in reduced collagen content as measured by hydroxyproline assay. *P < 0.05, unpaired t test. (**F** and **G**) Supernatants from day 28–transplanted lung homogenates were analyzed for ATX levels by sandwich ELISA (**F**) and ATX activity utilizing FS-3 fluorogenic substrate (**G**). *n* = 7–9. Data are represented as means ± SEM. **P < 0.01; ****P < 0.0001, simple linear regression.

Our in vitro investigations of human lung allograft–derived MCs suggested an obligatory role for MNK1-induced eIF4E phosphorylation at Ser209 in upregulation of key profibrotic autocrine protein ATX and fibrogenic transformation of these cells. Next, we aimed to investigate the in vivo significance of the MNK/eIF4E Ser209 signaling axis in regulating ATX and the pathogenesis of chronic allograft rejection by utilizing an established orthotopic murine lung-transplant model of chronic allograft rejection. These F1–to–parent mouse (B6D2F1/J→C57BL/6J) left lung transplants mimic RAS, a particularly aggressive form of CLAD marked by robust peribronchial and pleural fibrosis (36). Lung allograft recipients were treated with MNK1/2 inhibitor eFT-508 (5 mg/kg) or vehicle administered once daily by oral gavage from days 7 to 28 after transplant (Figure 6A). Trichrome staining demonstrated decreased bronchovascular bundle fibrosis (Figure 6B). A 50% reduction in transplant lung hydroxyproline content, a measure of total collagen levels, was noted upon eFT-508 administration when compared with placebo controls (Figure 6C). We have recently demonstrated that Gli1^+^ bronchovascular bundle MCs play a key role in allograft fibrogenesis (10). Gli1^CreERT2/WT^;Rosa26^mTmG/WT^ mice treated with eFT-508 confirmed decreased expansion of this cell population with MNK inhibition (Figure 6D). To investigate whether the effect of MNK inhibition on allograft fibrogenesis was predominantly by its effect on the MC functions and to determine whether it can be utilized in established CLAD, treatment was started at day 28 when peak fibrosis occurs. These allografts were harvested at day 40, and collagen content quantitated by hydroxyproline assay was found to be lower in eFT-508–treated allografts compared with vehicle (Figure 6E). To determine the in vivo effect of MNK inhibition on ATX expression, protein levels were measured in transplant lung homogenates by ELISA. A 50% decrease in lung ATX expression was noted upon eFT-508 treatment (Figure 6F). Consistent with reduced ATX levels, ATX activity, as measured with the fluorogenic substrate FS-3, was significantly reduced in lung allografts administered eFT-508 compared with placebo controls (Figure 6G).

Utilizing gene-deleted mice as donors versus recipients in transplant models allows for delineation of the specific contribution of resident somatic versus infiltrating immune cells to disease pathogenesis. Our murine lung-transplant model utilizes lung grafts from B6D2F1/J, which are the first filial generation of C57BL/6J and DBA2/J. This precluded *Eif4e^Ser209A/A^* and *Mnk1/2*-KO mice being used as donors. However, *Eif4e^Ser209A/A^* mice and *Mnk1/2*-KO C57BL/6J mice were utilized as recipients and allograft fibrosis assessed by hydroxyproline assay and trichrome staining. No difference was seen in allograft fibrosis between B6D2F1/J grafts transplanted into WT, *Eif4e^Ser209A/A^*, and *Mnk1/2*-KO mice, suggesting that the protective effect of MNK1 inhibition on allograft fibrogenesis is not mediated via immune cells (Supplemental Figure 1, A–D). Flow cytometry was utilized to compare infiltrating immune cell populations in these allografts placed into WT, *Eif4e^Ser209A/A^*, and *Mnk1/2*-KO mice, and no significant differences were noted (Supplemental Figure 1E).

**Figure 7:**
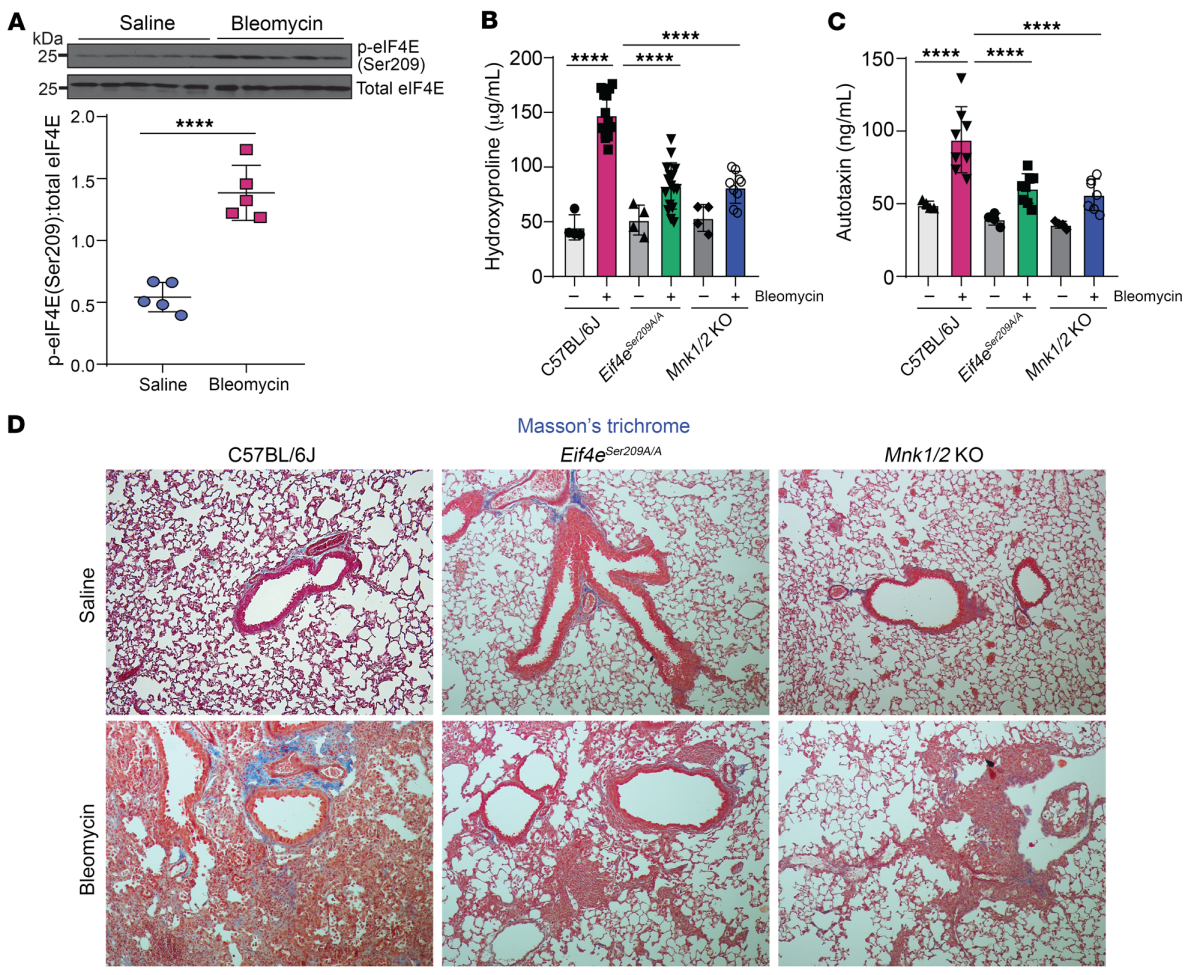
Genetic deficiency of *Eif4e^Ser209A/A^* and *Mnk1/2* prevents collagen deposition and ATX synthesis in bleomycin-injured mice. (**A**) Representative immunoblot and densitometric analysis of phospho-eIF4E (Ser209) from lung homogenates of mice 21 days after bleomycin administration. *n* = 5. Unpaired *t* test. (**B**) Quantitation of collagen content in lung homogenates of C57BL/6J, *Eif4e^Ser209A/A^*, and *Mnk1/2*-KO by hydroxyproline assay. *n* = 4 in saline control groups; *n* = 9–15 in bleomycin-treated groups. One-way ANOVA; post hoc test: Bonferroni’s test. (**C**) ATX protein expression levels in saline- and bleomycin-treated lung homogenates, as measured by sandwich ELISA. *n* = 4–8. One-way ANOVA; post hoc test: Bonferroni’s test. Data are represented as means ± SEM. ***P < 0.001; ****P < 0.0001. (**D**) Representative images of trichrome staining of day-21 tissue sections of C57BL/6J, *Eif4e^Ser209A/A^*, and *Mnk1/2*-KO mice administered bleomycin or saline control. Original magnification, ×200. *n* = 6 mice for each group.

To further validate the role of the MNK/eIF4E-S209 axis in lung fibrogenesis, we utilized another murine model in which intratracheal administration of bleomycin leads to lung fibrosis with evidence of peribronchial fibrosis. Lung homogenate from bleomycin-treated lungs demonstrated increased eIF4E phosphorylation at Ser209 by Western blot analysis as compared with that in saline-treated controls (Figure 7A). C57BL/6J (WT), *Eif4e^Ser209A/A^*, and *Mnk1/2*-KO mice were administered bleomycin, and the extent of fibrosis was investigated on day 21. Histology demonstrated decreased fibrosis in the peribronchial regions, and collagen induction in response to bleomycin was significantly reduced in *Eif4e^Ser209A/A^* and *Mnk1/2*-KO mice as compared with WT controls when quantitated by hydroxyproline assay (Figure 7, B and D). ATX expression in lung homogenates was measured by ELISA. While bleomycin-injured WT mice demonstrated an approximately 2-fold increase in ATX, no change was noted in *Eif4e^Ser209A/A^* and *Mnk1/2*-KO mice upon bleomycin-induced injury (Figure 7C).

## Discussion

Stable activation of various profibrotic signaling pathways is a well-recognized hallmark of transformed MCs in fibrotic tissues. Here, we characterize the MNK/eIF4E axis as a previously unrecognized translational control mechanism in MCs via which dysfunctional protein expression and tissue fibrosis are orchestrated. We found that increased phosphorylation of eIF4E at Ser209 is a consistent feature of MCs derived from fibrotic lung allografts and that activation of the MNK/eIF4E axis is critical for their fibrogenic transformation. Furthermore, we uncovered eIF4E phosphorylation as a regulator of key autocrine profibrotic mediator ATX and downstream β-catenin activation. Our preclinical studies demonstrating the efficacy of the MNK inhibitor in abrogating allograft fibrosis and ATX expression in a murine orthotopic lung-transplant model demonstrates the in vivo relevance of this pathway and its potential utility as a therapeutic strategy for CLAD.

We were the first, to our knowledge, to identify a resident population of mesenchymal progenitors in human adult lungs by demonstrating the donor origin of mesenchymal stromal cells isolated from BAL of sex-mismatched human lung allografts (8). Unique expression of embryonic lung mesenchyme-associated transcription factors, among them Foxf1, established the tissue specificity of these cells (9). Expansion, mobilization, and stable fibrotic transformation of these graft-derived Foxf1^+^ collagen I–expressing (Col1-expressing) MCs was shown to accompany development of CLAD in human translational studies (3, 9, 11–13, 37, 38). Utilizing single-cell RNA-Seq and a double-*Foxf1/Col1* transgenic mouse, we recently established that Foxf1-expressing MCs represent a transcriptionally distinct Gli1^+^Sca1^+^ subset of Col1-expressing MCs and reside in a subepithelial niche along the bronchovascular bundles in an adult lung (39). Foxf1^+^Gli1^+^Col1^+^ cells were found to expand and contribute to allograft fibrosis in the murine lung-transplant model (39). Combined, our clinical and preclinical investigations established the role of these transcriptionally and topographically distinct subsets of graft-resident MCs as effector cell of fibrosis in chronically rejecting lung allografts. In the present study, we utilize these functionally relevant human MCs and the murine lung-transplant model to investigate the role of the MNK/eIF4E axis in allograft fibrogenesis. We demonstrate that an mTORC-independent MNK/eIF4E signaling axis is active in human CLAD MCs and that MNK-induced Ser209 phosphorylation of eIF4E plays a critical role in regulating their fibrotic functions. Treatment with a small molecule MNK inhibitor (eFT-508) was demonstrated to decrease collagen expression and specifically thwart expansion of Foxf1^+^Gli1-expressing graft-resident bronchovascular bundle MCs in the murine lung-transplant model. This demonstration of a previously unrecognized role of the MNK/eIF4E signaling axis in maintaining fibrogenic functions of MCs represents what we believe to be a novel paradigm in understanding and targeting the progressive nature of fibrosis with potential relevance beyond lung transplantation.

We delineate a unique JNK/MNK/eIF4E signaling axis in fibrotic MCs with an interesting and surprising finding that JNK is the primary kinase upstream of constitutively increased phosphorylation of eIF4E in these cells derived from chronically rejecting lung allografts. JNK1 upregulation was also found to be sufficient, though in an MNK1-dependent manner, to induce eIF4E phosphorylation in non-Fib-MCs. Among the MAPK pathways, p38 and ERK1/2 are the most described in the regulation of MNK/eIF4E phosphorylation (33, 40). However, the role of JNK in mediating oxidative stress–induced eIF4E phosphorylation has been described in hepatocellular carcinoma (41), and JNK activation was noted to increase eIF4E phosphorylation in fetal MC lines (42). We have also previously identified JNK1 as a regulator of mSin1, a critical mTORC2 component, and provide evidence for a fibrotic activation pathway that involves the JNK/mSin1/mTORC axis, by which JNK-induced mSin1 expression drives mTORC2 activity, AKT phosphorylation, and subsequent translational activation (12). These data, together with direct evidence of an increase in cap-dependent translation noted in MCs transfected with active JNK1 shown in the present study, suggest a previously unappreciated role of MAPK JNK in modulating fibrogenic activation of MCs via both mTORC/4EBP/eIF4E and MNK/eIF4E Ser209 pathways.

Our studies uncover a unique link of the MNK/eIF4E axis to the ATX/LPA signaling axis, a finding which is of significance in the context of both fibrogenic and oncogenic transformation and has relevance to a wide variety of diseases. ATX, a secreted glycoprotein, profoundly affects the local microenvironment via its effect on the generation of LPA, with downstream LPA receptor signaling driving fibrosis (43–46). ATX is widely recognized to be regulated at the transcriptional level, with the most well-studied activator being NFAT1 (47–49). We have previously demonstrated an autocrine loop in CLAD MCs where increased ATX expression induces NFAT1 activation via LPA1-induced calcium signaling and further promotes *ATX* mRNA expression (3). We have also recently identified Foxf1 as a transcriptional repressor of *ATX* in these graft-resident lung MCs (14). MC activation was marked by loss of Foxf1 and increased *ATX* (*ENPP2*) mRNA expression (10, 50). Our present findings now add another dimension of regulation of ATX by demonstrating that in these activated MCs with upregulated *ATX (ENPP2)* mRNA, MNK-induced eIF4E phosphorylation regulates ATX protein expression. Lung MCs from mice with nonphosphorylatable eIF4E demonstrated lower ATX expression, building on previously published work where ENPP2 was noted to be the highest among mRNAs more actively translated in embryonic fibroblasts derived from WT versus *Eif4e^Ser209A/A^* mice (14). Transfection with constitutively phosphorylated (*S209D*) mutant forms of eIF4E in nonfibrotic human MCs increased ATX expression. More importantly, in fibrotic MCs, transfection with nonphosphorylatable eIF4E mutant and inhibition of MNK1 were sufficient to abrogate the constitutively high ATX expression, suggesting the MNK/eIF4E axis as the final step in the regulation of autocrine ATX in activated MCs.

Regulation of ATX protein expression by eIF4E phosphorylation also links the MNK/eIF4E axis to an important transcriptional regulator, β-catenin. GPCR signaling is now recognized as an important non-WNT mechanism of β-catenin activation (51, 52), and we have previously shown that ATX/LPA signaling via LPA1 induces glycogen synthase kinase-3β (GSK3β) phosphorylation, with resulting cytoplasmic accumulation and nuclear translocation of β-catenin in lung MCs (3, 13). Here, we demonstrate that targeting MNK1 or eIF4E phosphorylation inhibits constitutive β-catenin nuclear localization seen in fibrotic MCs. Active MNK1 overexpression induced β-catenin activation in nonfibrotic MCs, which was shown to be ATX dependent. These findings of the MNK/eIF4E axis regulating β-catenin activation via ATX/LPA/LPA1 signaling are supported by a previous study in chronic myeloid leukemia granulocyte-macrophage progenitors where MNK kinase–dependent eIF4E phosphorylation at Ser209 was shown to be specifically linked to nuclear translocation and activation of β-catenin (15). Linking eIF4E phosphorylation with β-catenin activation via ATX as an intermediary provides a potential targetable signaling mechanism of cellular transformation.

Both MNK and eIF4E phosphorylation have been demonstrated to be dispensable in normal development and standard growth conditions (16, 26), making MNK an attractive therapeutic target. MNK inhibition has been shown to be effective in animal models of cancer, and the MNK inhibitor eFT-508 has already entered human clinical trials in this field (22, 53). However, MNK inhibitors have not been studied in fibrosis and this is the first study, to our knowledge, to investigate the role of the MNK/eIF4E Ser209 pathway in an animal model of fibrosis. MNK inhibitor eFT-508 demonstrated strong antifibrotic actions in the murine orthotopic lung-transplant model and was associated with decreased quantitative collagen expression in lung allografts when given both early and late after transplant. Lack of protection from allograft fibrosis in *Eif4e^Ser209A/A^* and *Mnk1/2*-KO recipient mice in the lung-transplant model further suggested that the protective effect of targeting the MNK/eIF4E pathway is not secondary to its effects on host-derived alloimmune responses. This is important, as MNK has a reported role in the proinflammatory signaling of macrophages and T cells and MNK inhibitors are reported to blunt cytokine production in macrophages and Th17 T cells (54, 55). Furthermore, recent work in melanoma suggests a role for eIF4E phosphorylation in cellular phenotypic switching, with consequences for local immune responses (56). More in-depth work is needed to establish whether MNK-eIF4E activation regulates immunomodulatory functions of MCs along with fibrotic differentiation.

Lack of meaningful therapeutic strategies for CLAD has consequentially limited successful long-term outcomes after lung transplantation. The efficacy of pharmacologic MNK inhibition in the preclinical model of CLAD is of abundant clinical interest. Amelioration of allograft fibrosis was noted at early and late time points, suggesting its role as both a preventive and therapeutic strategy. These findings are particularly relevant, as the murine model (B6D2F1/J→C57BL/6J) utilized in these studies mimics RAS, the CLAD phenotype with worse prognosis and severely limited survival (2). Furthermore, evidence of increased eIF4E-ser209 phosphorylation in both BOS and RAS human MCs and the inhibition of bronchovascular bundle fibrosis by MNK inhibitors, a key component of both BOS and RAS phenotypes, suggests the wider relevance of clinically targeting this pathway in CLAD. Future investigation and validation of the effect of MNK1/2 inhibitors in other murine transplant models of allograft fibrogenesis can further strengthen these findings and pave the way for future clinical trials.

In summary, we demonstrate that eIF4E phosphorylation and the JNK/MNK/eIF4E/ATX/β-catenin axis are critical players in the fibrogenic transformation of MCs, offering avenues for intervention in fibrotic diseases.

## Methods

### Sex as a biological variable.

MCs were cultured from lavage fluid (BAL fluid) derived from lung-transplant patients of both female and male sexes (Table 1). Male and female mice were utilized in mouse lung-transplant experiments. None of the experiments were limited to samples from either sex. Sex was not evaluated as a biological variable in the experiments.

### Primary cell isolation and cell culture.

Human MCs were isolated from BAL fluid of transplant recipients and expanded on standard cell culture dishes as described previously (8). CLAD diagnosis and phenotyping were performed in accordance with International Society for Heart and Lung Transplantation (ISHLT) guidelines (34) and as previously described (35). CLAD onset date was assigned as the first date of spirometric decline. CLAD samples were defined as BAL obtained 90 days or less before CLAD or after CLAD onset date, and MCs derived from these samples were labeled Fib-MCs. MCs derived from control CLAD-free patients with no evidence of rejection or infection at the time of BAL collection were labeled non-Fib-MCs. MCs cultured from individual patient’s BAL samples were treated as unique cell lines. DMEM containing high glucose and glutamine (Invitrogen, catalog 11965) supplemented with penicillin/streptomycin (100 units/mL) and amphotericin B (0.5%) was utilized as a growth medium, and cells were utilized at passages 3 through 6 in all experiments. For treatment experiments, MCs were plated into 6 cm dishes and grown to 80% confluence, followed by overnight serum starvation. Cells were then treated for 16 hours with eFT-508 (10 μM), AZD8055 (250 nM; Selleck Chemicals), or rapamycin (250 nM; Cayman Chemical) in serum-free medium.

### Protein isolation and immunoblotting.

Conditioned medium was harvested, and protein lysates were collected from treated or untreated cells lysed directly in-well utilizing 1× cell lysis buffer (Cell Signaling Technology) supplemented with 1× phosphatase inhibitor cocktail. Lysates were separated on 4%–12% Bis Tris gels (Invitrogen) prior to transfer onto PVDF membranes, followed by immunoblotting. Antibodies against phospho-MNK1(Thr197/202) (2111), total MNK1 (2195), total eIF4E (9742), eIF4G (2498), phospho–4E-BP1 (Thr37/46) (2855), total 4E-BP1 (9644), raptor (2280), phospho-AKT (Ser473) (4060S), total AKT (9272), phospho–p70 S6K1 (Thr389) (9234), total p70 S6K1 (2708), cyclin D1 (2978), SPARC (8725) and CD44 (3570) were purchased from Cell Signaling Technologies. Antibodies against phospho-eIF4E (Ser209) (ab76256), ATX (ab140915), and total β-catenin (ab6302) were purchased from Abcam, with loading controls GAPDH (sc-365062) and SAM68 (sc-333), obtained from Santa Cruz Biotechnology Inc. Collagen I (PA5-29569) was purchased from Thermo Fisher Scientific; active β-catenin (no. 05-665) and Flag M2 (F1804) were purchased from Sigma-Aldrich.

### ELISA and activity assays.

Conditioned medium levels were assessed as previously described (3, 50) using the human ENPP2/ATX Quantikine ELISA Kit (catalog DENP20, R&D Systems) and fluorogenic phospholipid substrate FS-3 (L-2000, Echelon Biosciences) (57).

### m^7^GDP cap pulldown assay.

m^7^GDP agarose resin was prepared as previously described (58). MCs were grown to 80% confluence in 10 cm dishes, serum starved overnight, and treated with eFT-508 (10 μM, 16 hours). Cells were lysed utilizing cap pulldown buffer (50 mM HEPES-KOH pH 7.5, 150 mM KCl, 1 mM EDTA, 2 mM DTT, and 0.1% Tween 20) containing protease and phosphatase inhibitors. Lysates were freeze-thawed twice and centrifuged at 20,000 *g* for 25 minutes to remove cellular debris; this was followed by total protein quantification using BCA assay (Thermo Fisher Scientific); 500 μg of lysate was loaded into microcentrifuge tubes containing m^7^GDP resin and rotated at 4°C for 2 hours. After incubation, agarose resin was spun down, supernatant removed, and washed 3 times using cap pulldown buffer. Following washes, proteins were eluted from agarose resin by boiling (70°C for 10 minutes) with 4× LDS sample buffer containing dithiothreitol and resolved on 4%–12% Bis Tris gels, followed by immunoblotting.

### siRNA-mediated silencing.

Dharmacon SMARTpool ON-TARGETplus human siRNAs specific to *RPTOR* (M-004107-01), *MNK1* (L-004879-00), *EIF4E* (L-003884-00), *ATX (ENPP2)* (L-004601-00), or nontargeting pool (D-001810-10) were utilized (Horizon Discovery). Briefly, MCs were plated to 50% confluence in 6-well plates overnight, followed by transfection with oligofectamine (Invitrogen) reagent in Opti-MEM medium utilizing 100 nM siRNA per well. The following day, medium was replaced with serum-free DMEM for 48 to 72 hours before protein harvesting and immunoblot analysis.

### Luciferase assays, MNK, and eIF4E plasmid generation and lentiviral infection.

pcDNA3-rLuc-PolIRES-fLuc cap-translation luciferase vector was a gift of Peter Bitterman (University Of Minnesota Health Clinics and Surgery Center Inc., Minneapolis, Minnesota, USA). pcDNA3.1-flag-MNK1-WT, MNK1-D191A (catalytically inactive), and MNK1-T344D (constitutively active) plasmids were a gift of Michael C. Brown (Duke University, Durham, North Carolina, USA). pLentilox-IRES-Puro- FLAG-MKKB2-JNK1α1 (constitutively active JNK1) was generated as previously described (12). To make lentiviral expression plasmids, coding sequences were digested out of pcDNA3 utilizing Nhe1 and Xho1 restriction enzymes, followed by ligation into pLenti-LoxEV (plasmid containing lentivirus expression vector a gift of the University of Michigan Vector Core). pcDNA3-3XFLAG-WT-eIF4E was a gift from Dylan C. Mitchell. pcDNA3-3XFLAG-eIF4E *S209A* (nonphosphorylatable) and *S209D* (constitutively phosphorylated) plasmids were generated by site-directed mutagenesis of pcDNA3-3XFLAG-WT-eIF4E utilizing the Q5 Site-Directed Mutagenesis Kit (New England Biolabs). Mutagenesis reactions were performed per the manufacturer’s instructions utilizing 63°C (for eIF4E-*S209D*) and 67°C (eIF4E-*S209A*) annealing temperatures and the following primers: eIF4E *S209D* forward primer: 5′-TAAGAGCGGCGACACCACTAAAAATAGG-3′ and reverse primer: 5′-GTAGCTGTGTCTGCGTGG-3′; and eIF4E *S209A* forward primer: 5′-TAAGAGCGGCGCCACCACTAAAA-3′ and reverse primer: 5′-GTAGCTGTGTCTGCGTGG-3′. Following mutagenesis, 3XFLAG-eIF4E inserts were digested out of pcDNA3 using BAMH1 and Xba1 followed by ligation into pLenti-LoxEV. All plasmids were verified by Sanger sequencing before use in cell experiments.

### Lentiviral infection and cell migration assay.

For lentiviral infection, MCs were plated to 60% confluence followed by the addition of 1× lentiviral solution in serum-free DMEM containing 8 μg/mL protamine sulfate linker. Twenty-four hours later, lentivirus was removed and DMEM containing 10% FBS was added for an additional 48 hours before protein harvesting. For luciferase assays, cells were subsequently reinfected with lentiviral particles containing cap-translation luciferase vector, followed by 24 hours in serum-containing media before harvest according to the manufacturer’s protocol (Promega Dual-Luciferase Reporter Assay System). Luminescence readings were performed utilizing a Promega GloMax Explorer. For cell-migration assay, lentiviral-infected MCs were serum deprived an additional 24 hours, trypsinized, and counted; 1 × 10^5^ MCs resuspended in serum-free DMEM were plated onto the top chamber of 8 μm Transwells (Corning, catalog 422) precoated with 1 mg/mL Matrigel (Corning, catalog 354234). DMEM containing 10% FBS was loaded onto the bottom chamber as the chemoattractant. After 16 hours, Transwells were removed to a fresh culture plate, the top chamber was wiped clean of any remaining cells, and 1 mg/mL MTT diluted in serum-free DMEM was added to the bottom chamber to incubate for 1 hour at 37°C. MTT solution was subsequently removed, and DMSO was added to the bottom chamber to dissolve the incorporated MTT; 100 μL aliquot of the solution was loaded onto 96-well plates and read at 570 nm on a Molecular Devices SpectraMax M3 plate reader. A standard curve obtained from plated dilutions of MCs was used to convert absorbance into cell numbers and calculate percentage of migration.

### Murine orthotopic left-lung transplant, bleomycin-injury model, and hydroxyproline assay.

C57BL/6J and B6D2F1/J mice were purchased from The Jackson Laboratory. *Mnk1/2*-KO mice and *Eif4e^Ser209A/A^* mice were provided by Rikiro Fukunaga (Osaka University of Pharmaceutical Sciences, Osaka, Japan) and in house, respectively. Gli1^CreERT2/WT^; Rosa26^mTmG/WT^ donor mice for transplant were generated by crossing tdTomato^fl^ (B6.129(Cg)-Gt(ROSA)26Sor^tm4(ACTB-tdTomato,-EGFP)Luo^/J (stock 007676; The Jackson Laboratory) and Gli1^CreERT2^ (Gli1^tm3(cre/ERT2)Alj^/J; stock 007913; The Jackson Laboratory) backcrossed 10 generations onto C57BL/6J background. Four- to six-week-old Gli1^CreERT2/WT^;Rosa26^mTmG/WT^ mice were given tamoxifen chow for 14 days, followed by 3 days of normal chow before transplantation. Orthotopic left-lung transplantation was performed as previously described using a modified cuff technique (36, 59–62). Isograft transplants were performed utilizing B6D2F1/→B6D2F1/J, with chronic restrictive allograft transplants utilizing the B6D2F1/J→C57BL/6J strain combination. Mice were administered eFT-508 (5 mg/kg/d; oral gavage) or vehicle control to transplanted mice between days 7 and 28 after transplant. The bleomycin injury experiments were performed by oropharyngeal administration of bleomycin (0.025 U) to naive C57BL/6J, *Mnk1/2*-KO, or *Eif4e^Ser209A/A^* mice. Animals were euthanized at experimental end points, and lungs were harvested and fixed in 10% neutral-buffered formalin solution or homogenized for hydroxyproline assay, as previously described (3, 36, 62).

### Flow cytometry, immunohistochemical staining, and murine fibroblast isolation.

Flow cytometric analysis was utilized to quantify infiltrating immune populations after transplantation. Minced lungs were digested to a single-cell suspension by rocking at 37°C for 40 minutes utilizing 0.1% collagenase A solution followed by dissociation with an 18-gauge needle attached to a syringe. Suspensions were filtered through a 70 μm strainer, and 1 × 10^6^ cells were stained with fluorescently conjugated antibodies for 30 minutes on ice to resolve CD45^+^CD3^–^CD19^–^Ly6G^–^, followed by CD3^+^CD4^+^ T cells, CD11c^+^CD24^+^CD103^+^ and CD11b^+^ conventional DCs, CD11b^+^Ly6C^+^ inflammatory monocytes, and CD64^+^CD11b^+^ exudative macrophages. CD45^+^CD3^-^Ly6G^–^CD19^+^ gates were used to distinguish B cells.

Immunohistochemical staining was performed as previously described (10) on 5 μm formalin-fixed, paraffin-embedded sections utilizing Cy3-conjugated mouse anti–α-smooth muscle actin (1:200, MilliporeSigma, catalog C6198) and FITC-conjugated goat anti-GFP (1:100, Abcam, ab6662). For collagen determination in tissues, Masson’s trichrome and Picrosirius red staining were performed according to the manufacturer’s protocol (IHC World). Morphometric analysis of pleural thickening was performed on Picrosirius red–stained sections as previously described (36). Images were taken utilizing an ECHO Revolve microscope.

Murine fibroblast isolation was performed by collagenase digestion of lungs to obtain single-cell suspensions, which were subsequently plated on 10 cm dishes in DMEM supplemented with 10% FBS. Dishes were rinsed 48 hours later, and fibroblasts were grown to confluence before passaging and protein harvest.

### Statistics.

When comparing the means of 2 groups, Student’s 2-tailed *t* test was used to determine *P* values. When comparing the means of 3 or more groups, 1-way ANOVA was performed with a post hoc Bonferroni’s test to determine which groups showed significant differences unless otherwise specified. A *P* value of less than 0.05 was considered significant and was analyzed using GraphPad Prism (version 8.0.0) for Windows 64-bit.

### Study approval.

The study was conducted in accordance with relevant guidelines and regulations using a protocol for human studies approved by the University of Michigan Institutional Review Board (approval number HUM00042443) in compliance with the Helsinki declaration, and all participants provided written, informed consent prior to participation in the study. All murine experiments were performed with approval of the Institutional Animal Care and Use Committee and the Institutional Review Board at the University of Michigan.

### Data availability.

Values for all data points in graphs are reported in the Supporting Data Values file. Additional data are available upon request.

## Author contributions

VNL and NMW designed research studies. NMW, YI, APM, KM, DCM, GGK, AML, and ALG performed experiments and acquired data. VNL, NMW, RV, NS, YI, APM, KM, DCM, GGK, AML, and ALG analyzed data. VNL, NMW, and RV prepared the manuscript.

## Supplementary Material

Supplemental data

Unedited blot and gel images

Supporting data values

## Figures and Tables

**Table 1:**
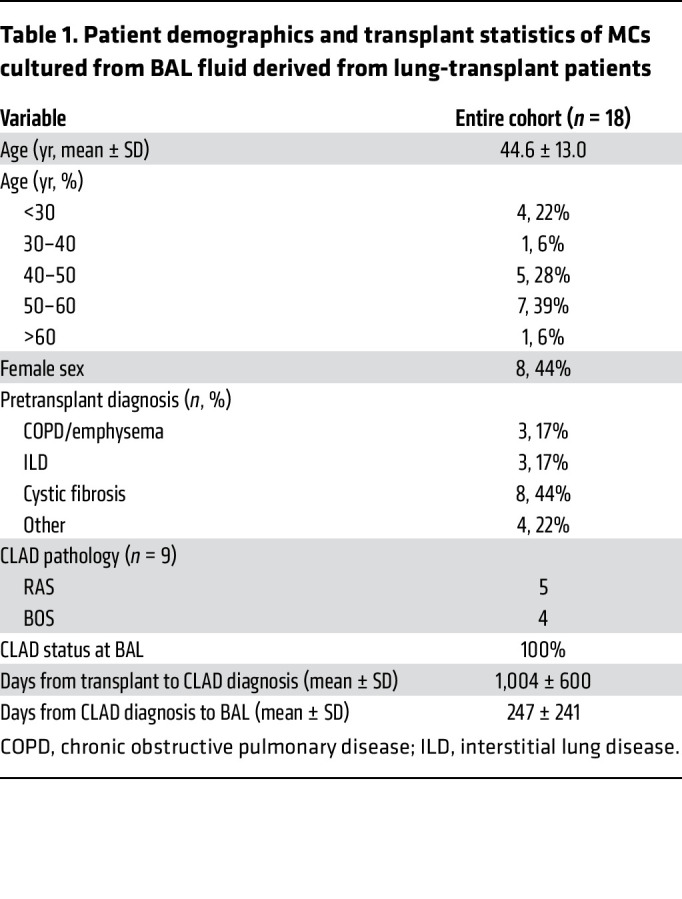
Patient demographics and transplant statistics of MCs cultured from BAL fluid derived from lung-transplant patients

## Version 1 08/15/2024

Electronic publication
