# Supplementary material for: MNK-driven eIF4E phosphorylation regulates the fibrogenic transformation of mesenchymal cells and chronic lung allograft dysfunction
Source: J Clin Invest. 2024 Aug 15;134(16):e168393. doi: 10.1172/JCI168393 (PMC11324311; doi:10.1172/JCI168393)
Supplement: Supplemental data [file jci-134-168393-s080.pdf]

SUPPLEMENTAL FIGURE 1

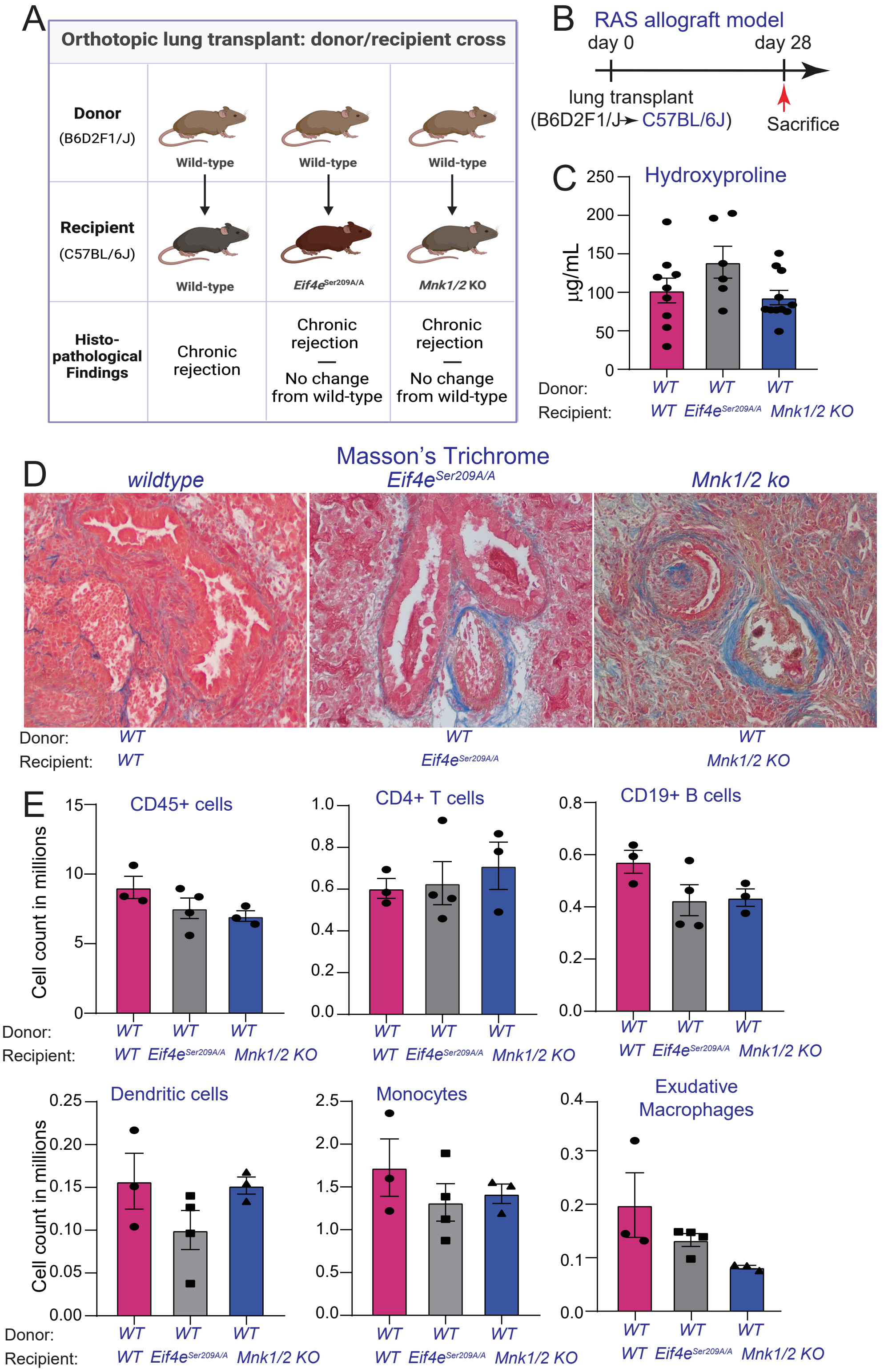

Supplemental Figure 1. **Genetically deficient Eif4e$^{Ser209A/A}$ and Mnk1/2 lung transplant recipients demonstrate lack of protection from allograft fibrosis.** Contribution of Mnk1/2/eIF4E signaling axis in the hematopoietic compartment to allograft fibrogenesis was investigated by utilizing *Eif4e*$^{Ser209A/A}$ and *Mnk*$^{1/2}$ knockout mice as recipients in the B6D2F1/J to C57BL/6J murine lung transplant model. Experimental outcomes and schematic representation shown in (**A**,**B**). Fibrosis was assessed by hydroxyproline assay and Masson's trichrome staining (**C**,**D**). Infiltrating immune cell populations isolated from collagenase-digested single cell suspensions were subjected to FACS analysis (**E**). Values are expressed as means ± SEM. Disclosure: Please note that the representative RAS allografts (wildtype) presented in (**D**) also appears in Figure 6B Masson's Trichrome.
