# Supplementary material for: MNK-driven eIF4E phosphorylation regulates the fibrogenic transformation of mesenchymal cells and chronic lung allograft dysfunction
Source: J Clin Invest. 2024 Aug 15;134(16):e168393. doi: 10.1172/JCI168393 (PMC11324311; doi:10.1172/JCI168393)
Supplement: Unedited blot and gel images [file jci-134-168393-s081.pdf]

FIGURE 1

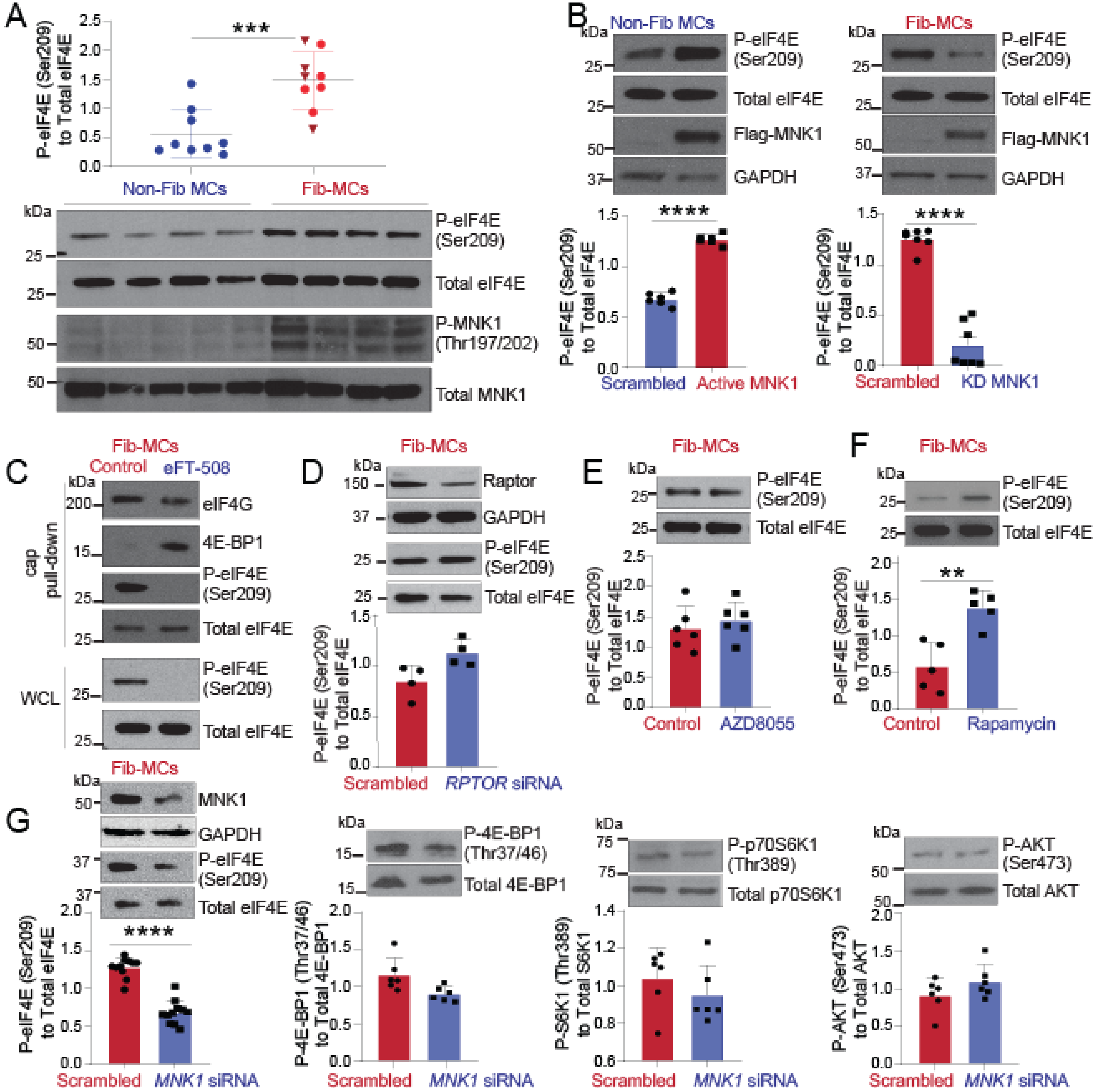

A
***
P-eIF4E (Ser209) to Total eIF4E
Non-Fib MCs
Fib-MCs
kDa
P-eIF4E (Ser209)
Total eIF4E
P-MNK1 (Thr197/202)
Total MNK1
B
Non-Fib MCs
Fib-MCs
P-eIF4E (Ser209)
Total eIF4E
Flag-MNK1
GAPDH
****
Scrambled
Active MNK1
KD MNK1
C
Fib-MCs
Control
eFT-508
cap pull-down
eIF4G
4E-BP1
WCL
D
Raptor
RPTOR siRNA
E
Control
AZD8055
F
**
Rapamycin
G
MNK1
P-4E-BP1 (Thr37/46)
Total 4E-BP1
P-p70S6K1 (Thr389)
Total p70S6K1
P-AKT (Ser473)
Total AKT
P-4E-BP1 (Thr37/46) to Total 4E-BP1
P-S6K1 (Thr389) to Total S6K1
P-AKT (Ser473) to Total AKT
MNK1 siRNA

# Figure 1A: Phospho and Total eIF4E

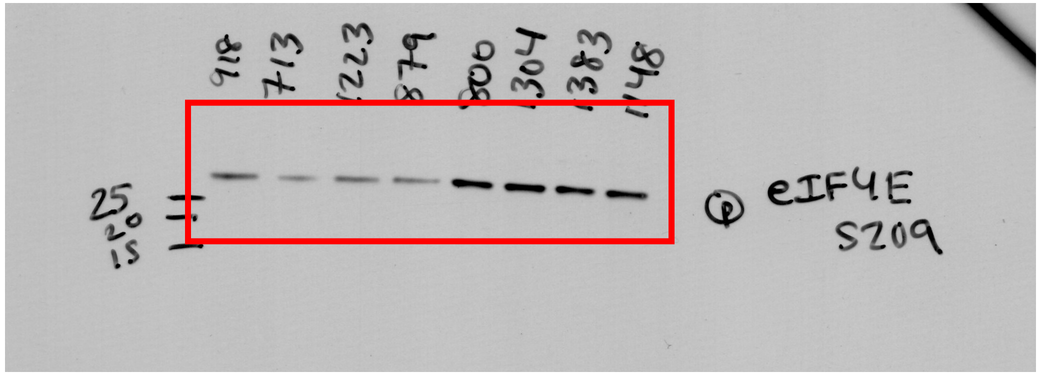


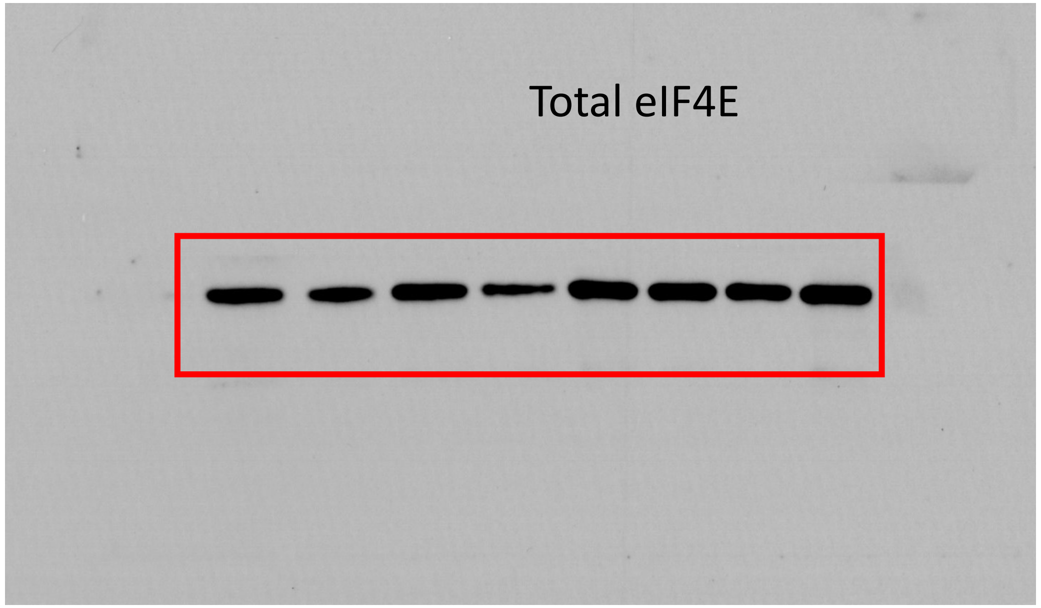

# Figure 1A: Phospho and Total MNK1

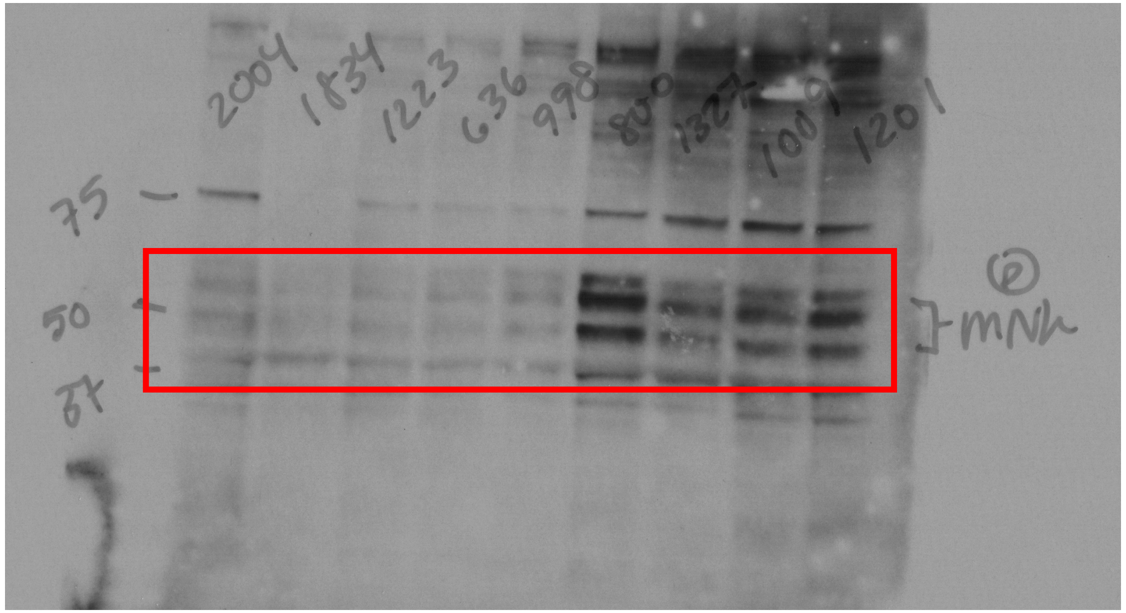


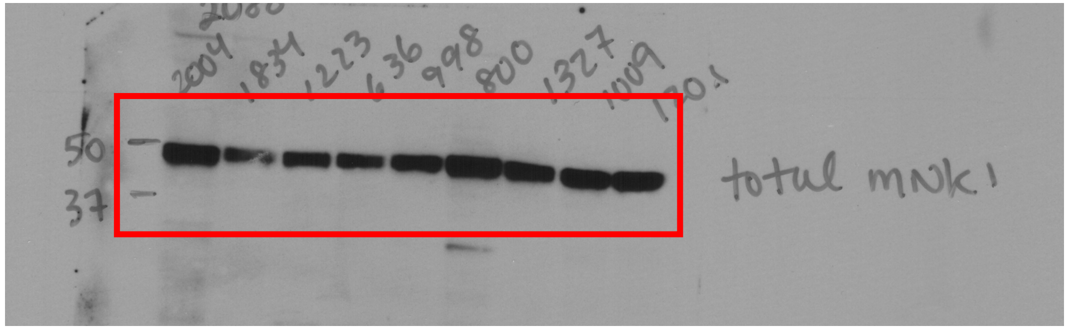

# Figure 1B: Active/KD MNK figure

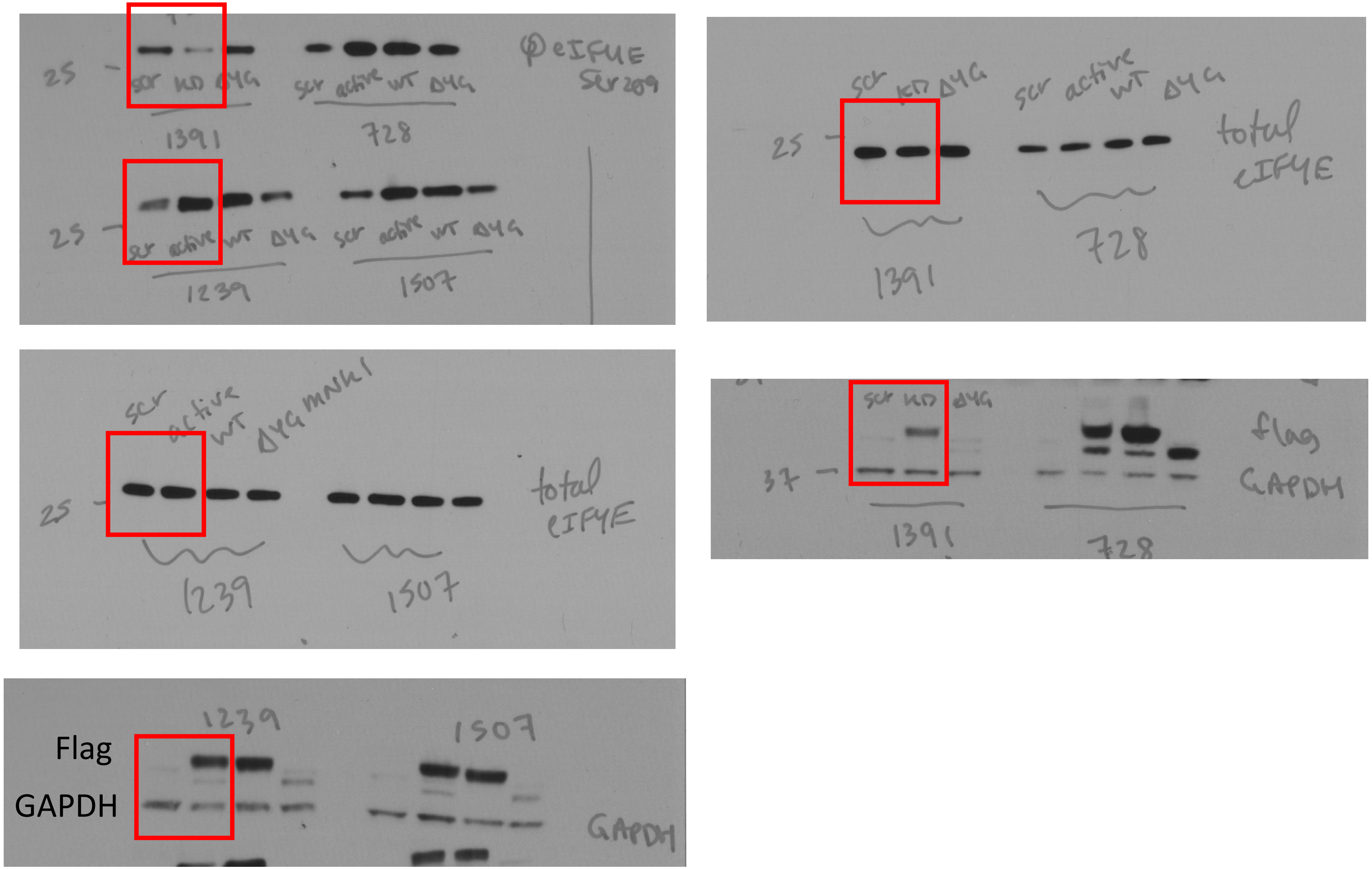

# Figure 1C: cap pull down figure

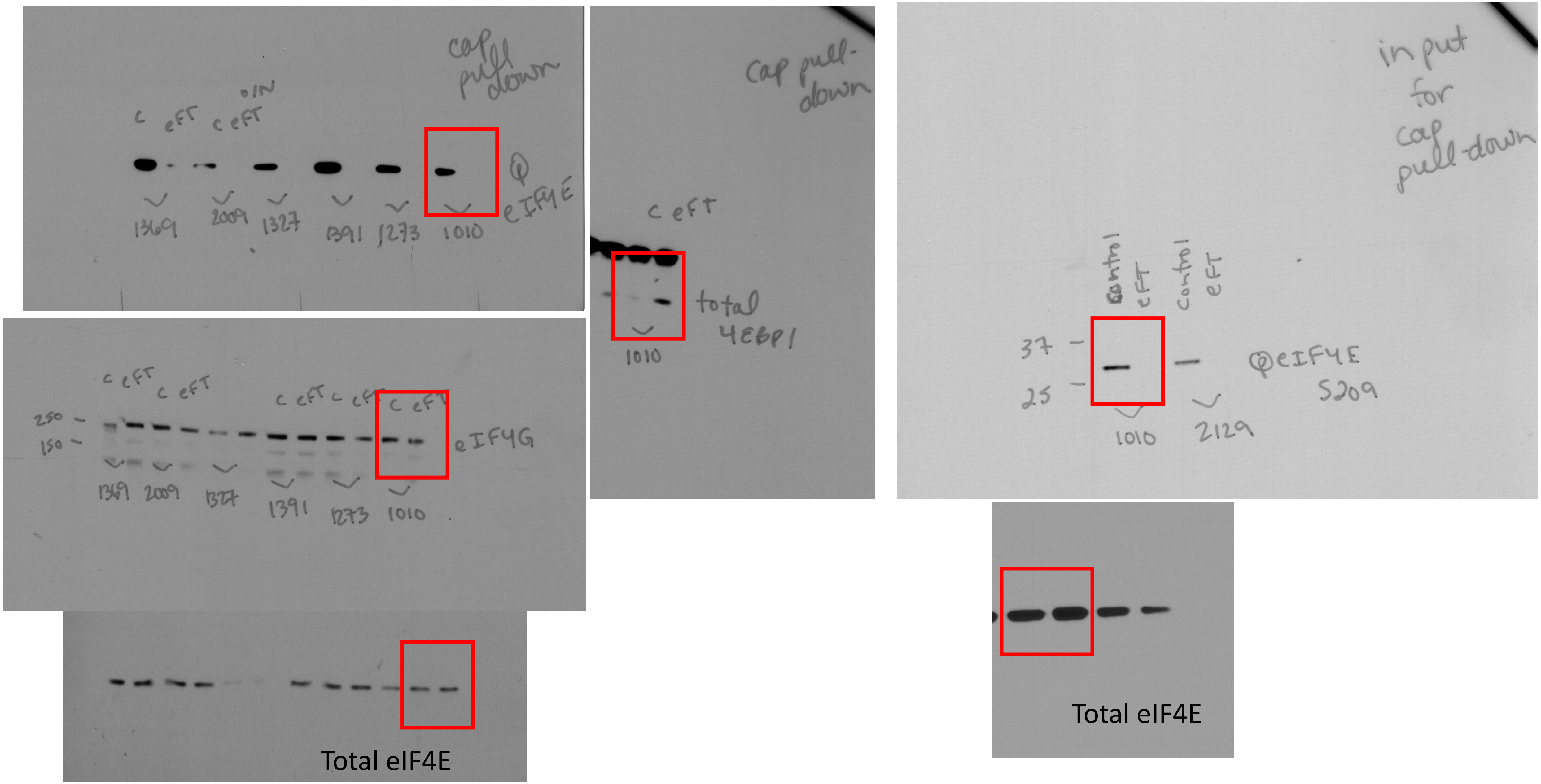

# Figure 1D: Raptor silencing

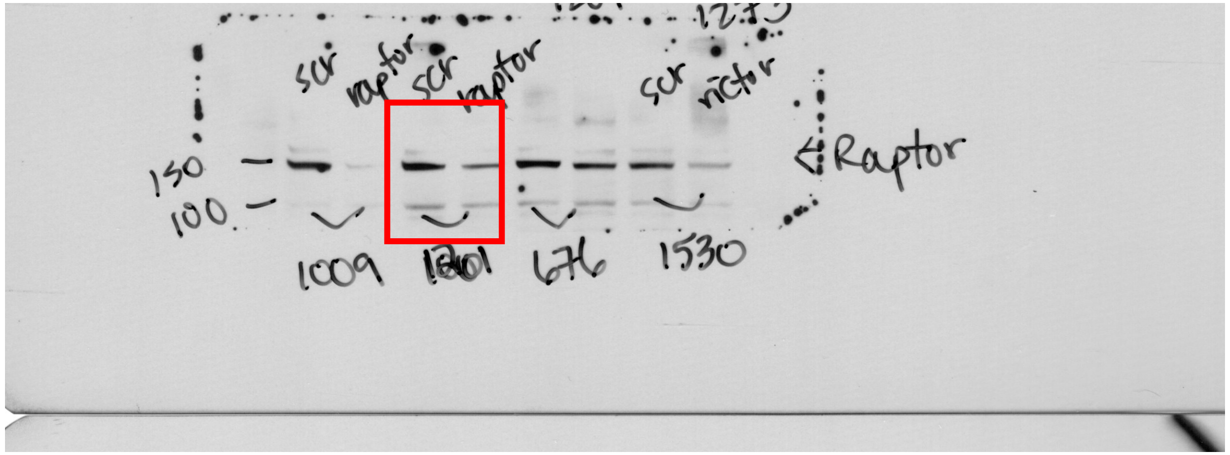


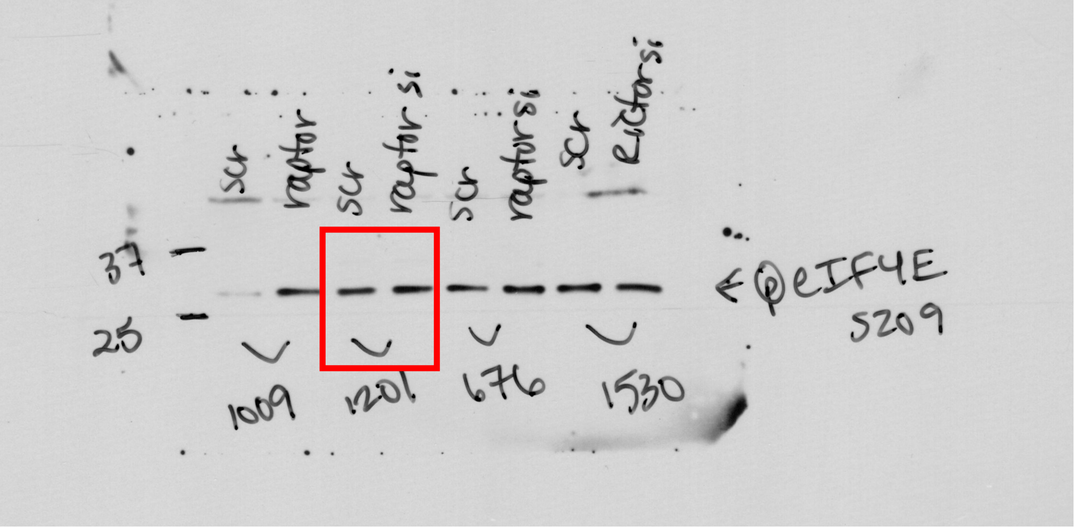


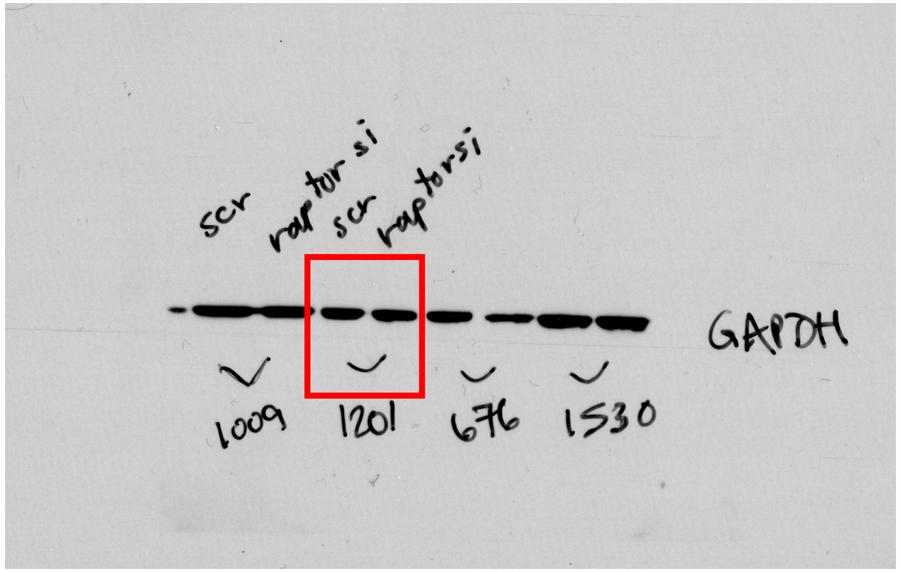


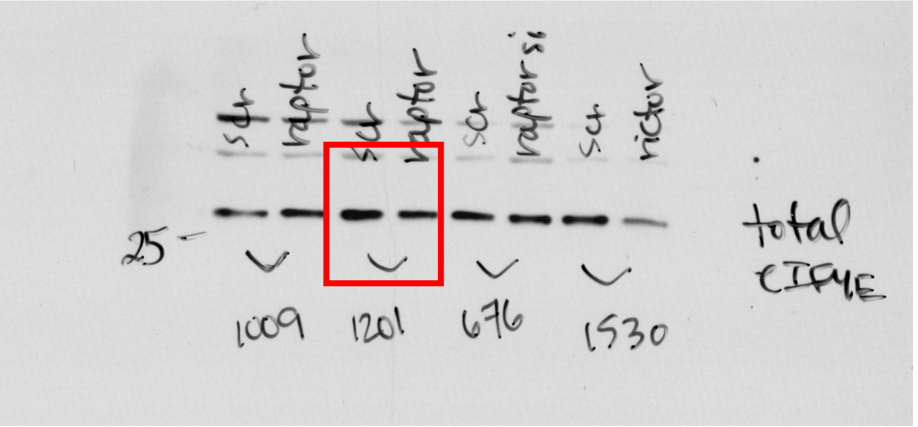

# Figure 1E,F: AZD and rapa treatments

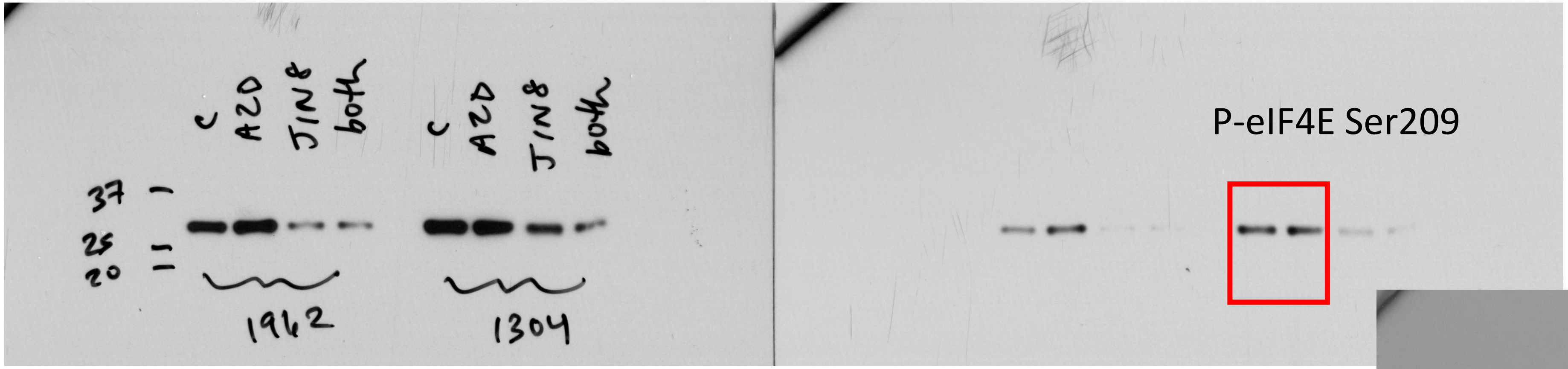


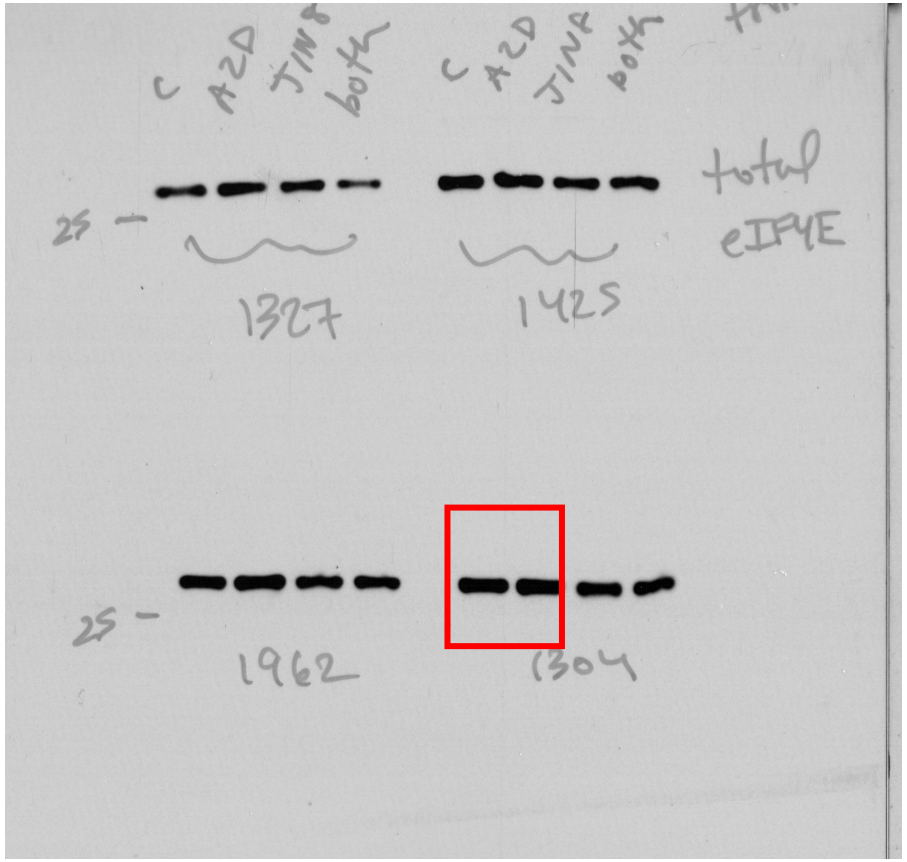


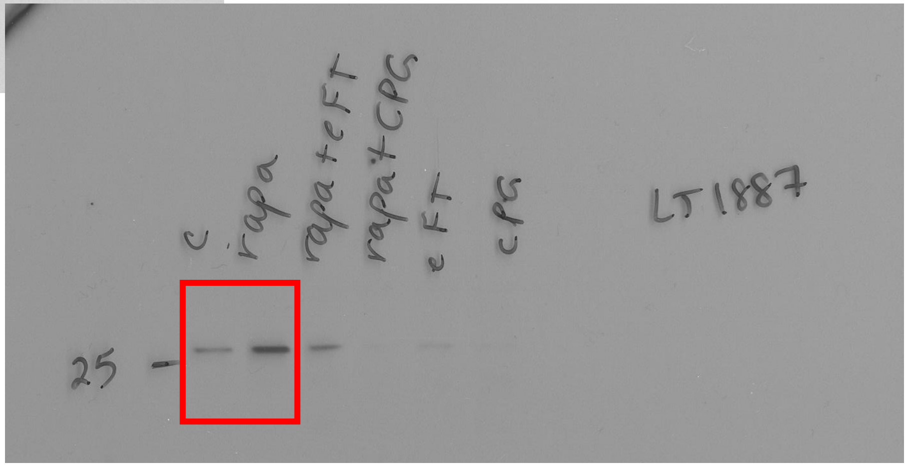


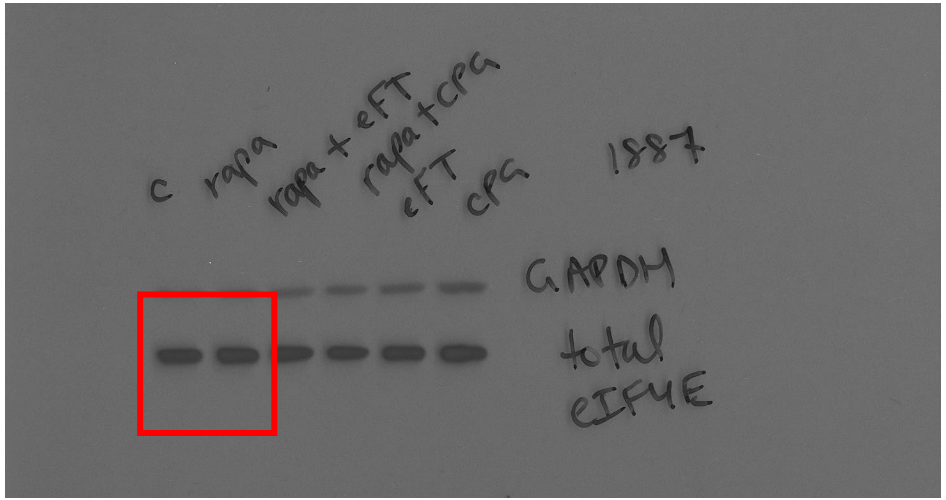

# Figure 1G: MNK silencing probed for mTORC endpts

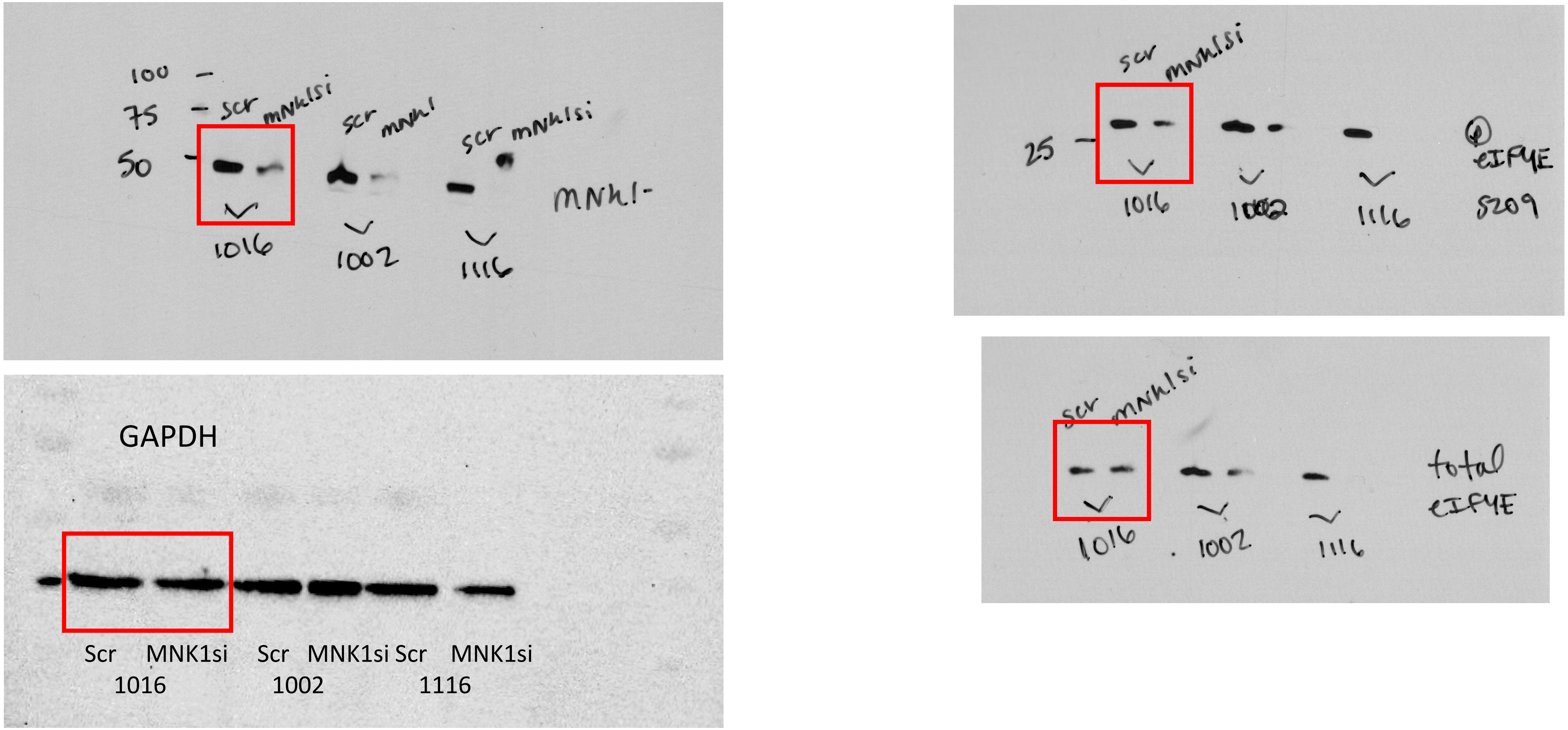

# Figure 1G: MNK silencing probed for mTORC endpts

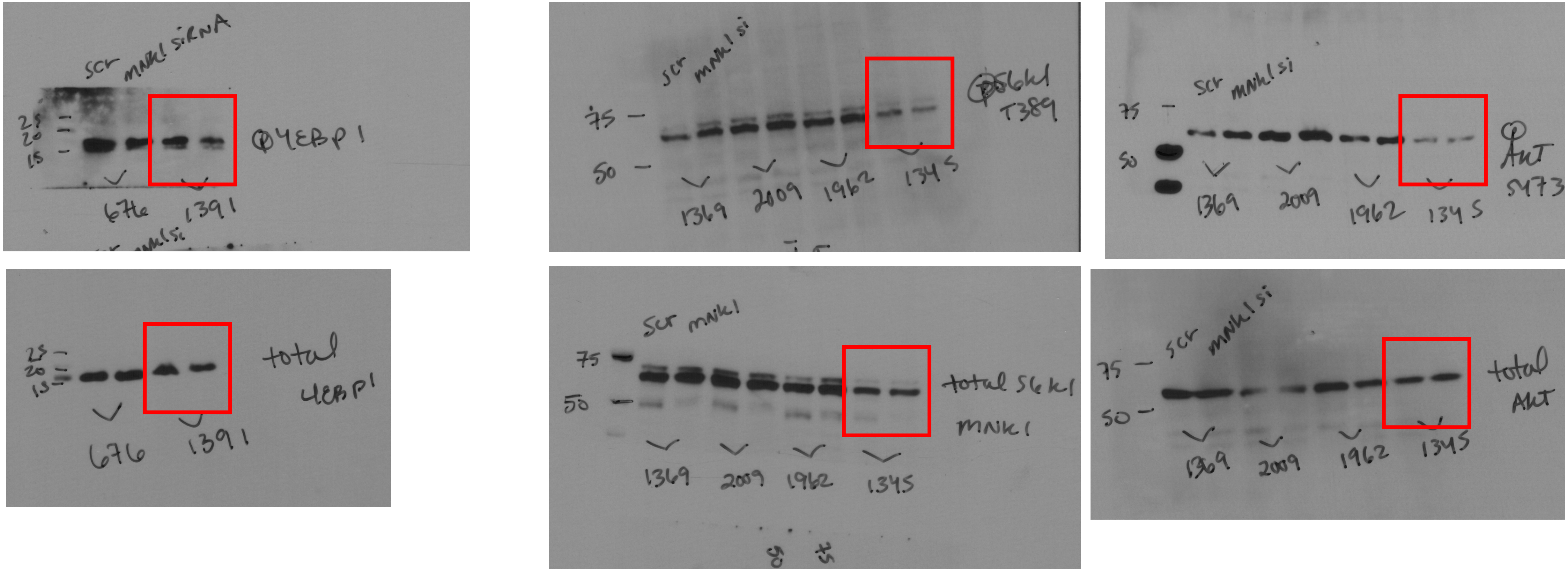

FIGURE 2

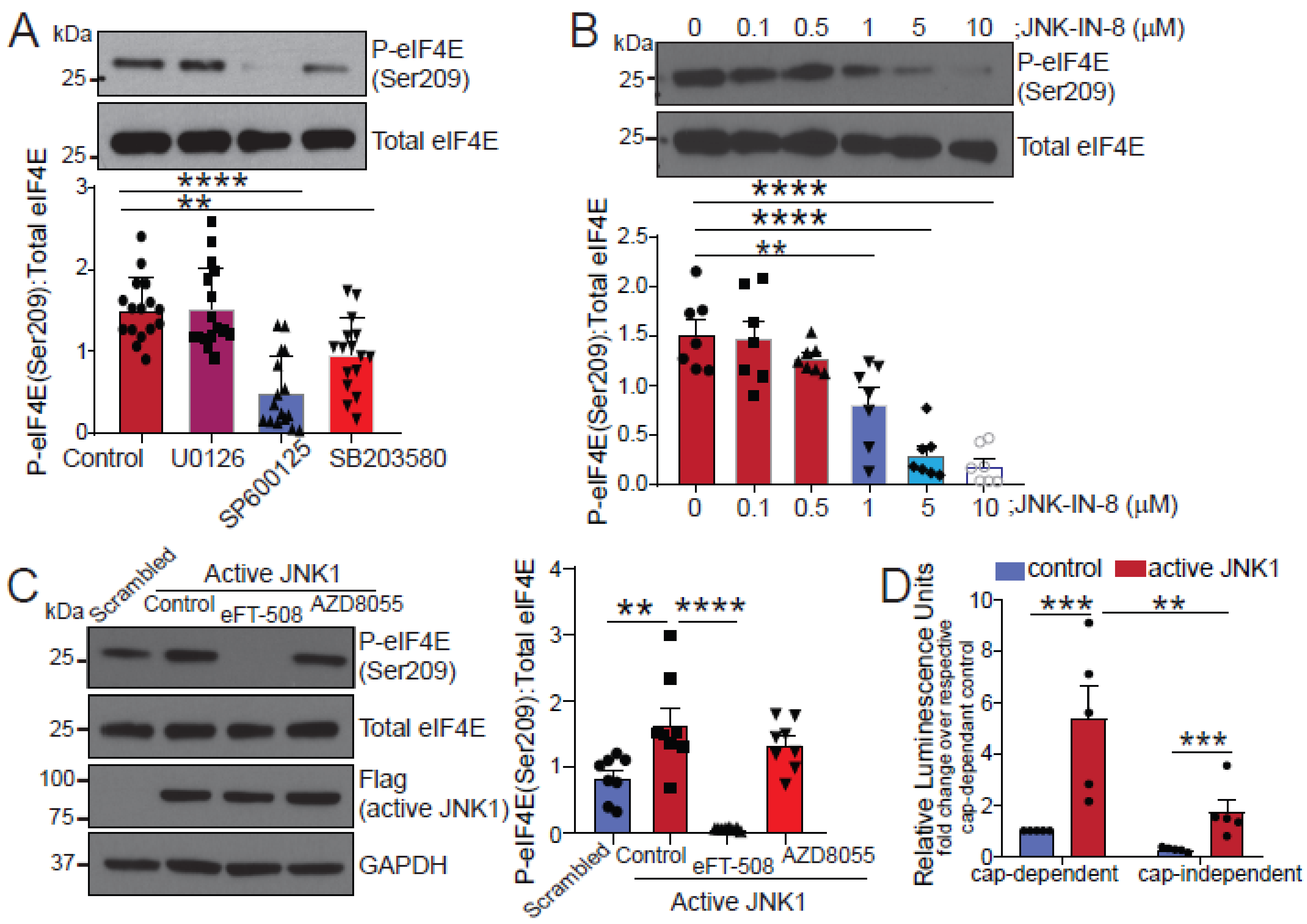
A
kDa
25
P-eIF4E (Ser209)
25
Total eIF4E
P-eIF4E(Ser209):Total eIF4E
****
**
0
1
2
3
Control
U0126
SP600125
SB203580
B
0 0.1 0.5 1 5 10 ;JNK-IN-8 (µM)
kDa
25
P-eIF4E (Ser209)
25
Total eIF4E
P-eIF4E(Ser209):Total eIF4E
****
****
**
0.0
0.5
1.0
1.5
2.0
2.5
0 0.1 0.5 1 5 10 ;JNK-IN-8 (µM)
C
kDa
Scrambled
Active JNK1
Control
eFT-508
AZD8055
25
P-eIF4E (Ser209)
25
Total eIF4E
100
75
Flag (active JNK1)
37
GAPDH
P-eIF4E(Ser209):Total eIF4E
**
****
0
1
2
3
4
Scrambled
Control
eFT-508
AZD8055
Active JNK1
D
control
active JNK1
Relative Luminescence Units
fold change over respective cap-dependant control
***
**
***
0
2
4
6
8
10
cap-dependent
cap-independent

# JNK figure 2A

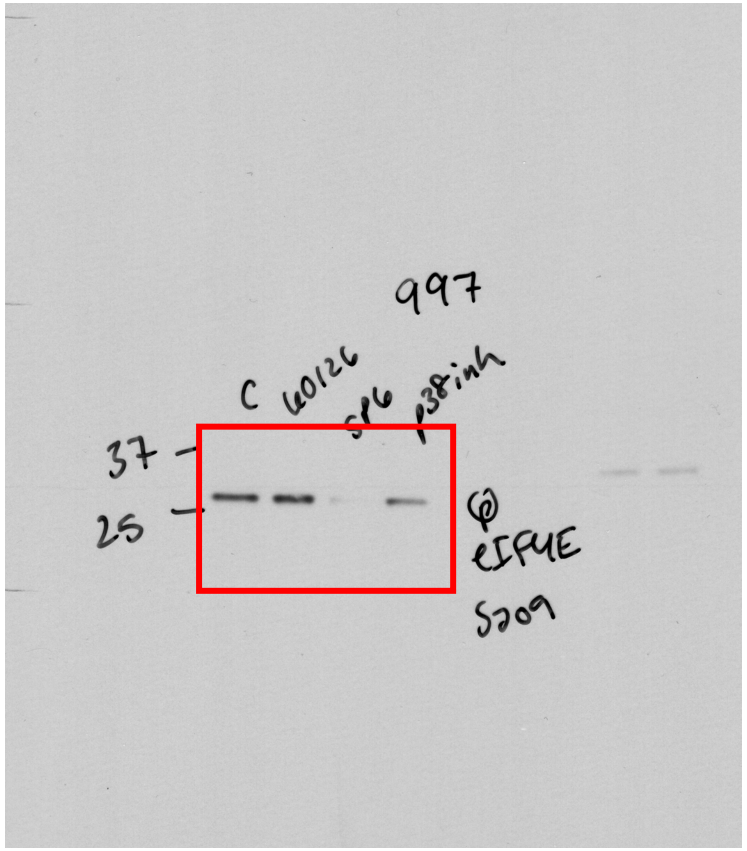


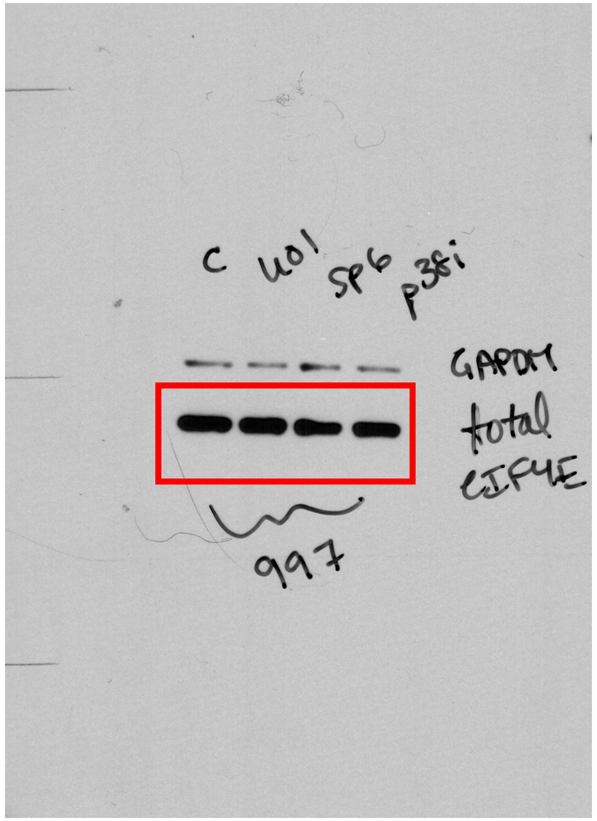

# JNK figure 2B

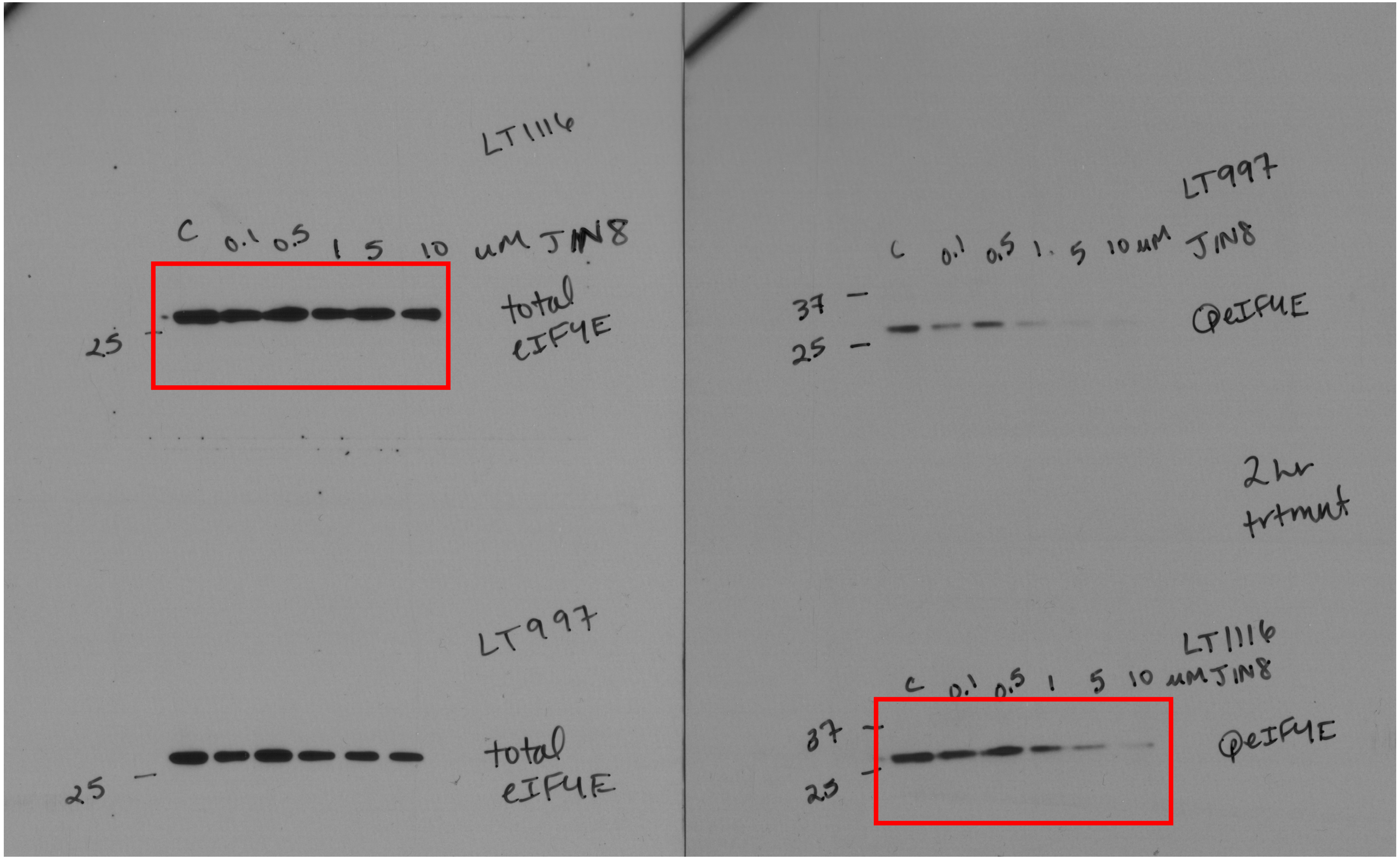

# JNK figure 2C

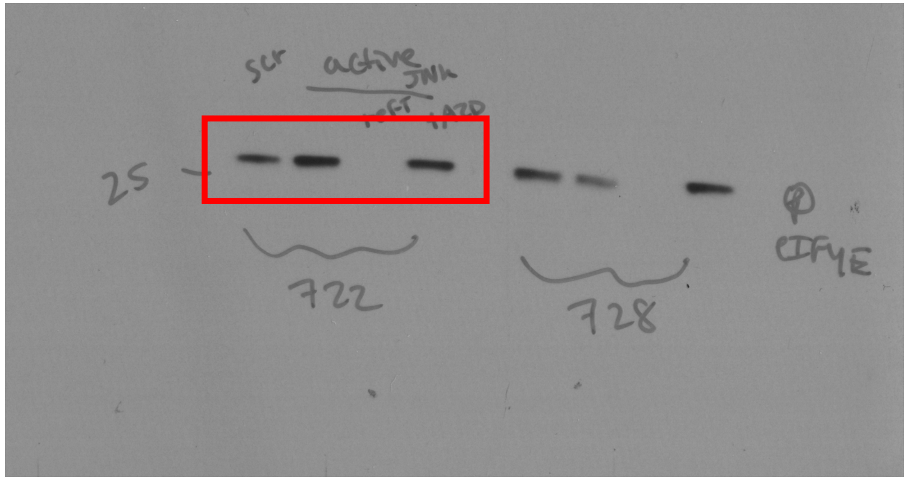


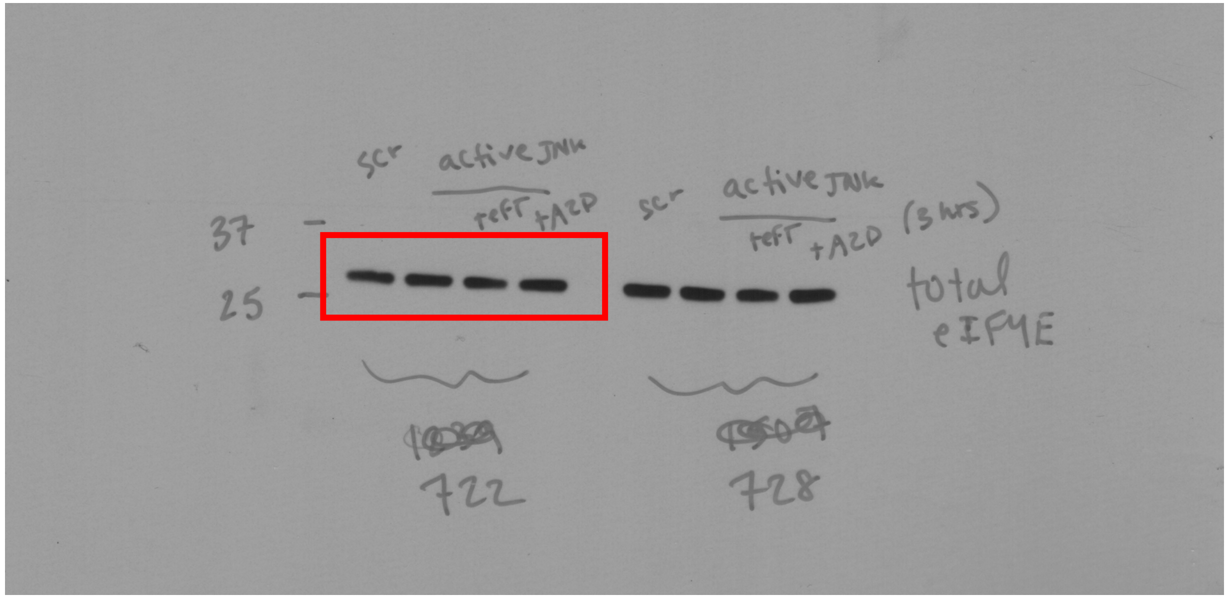


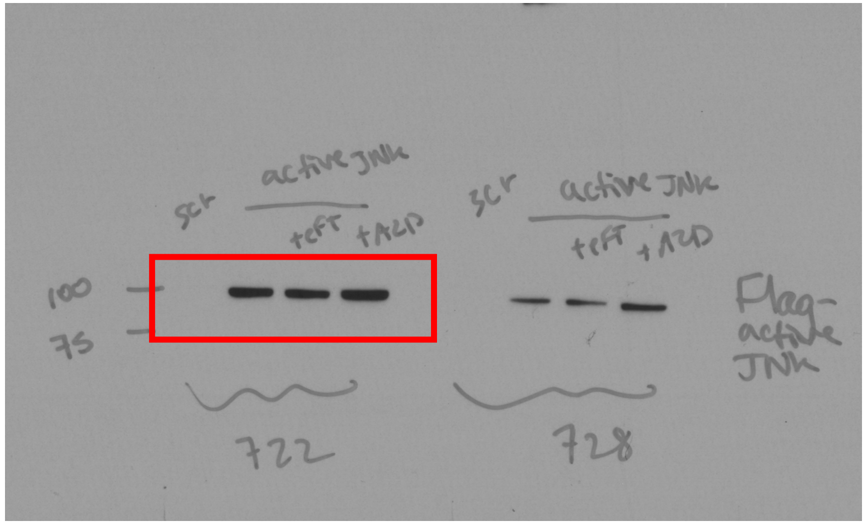


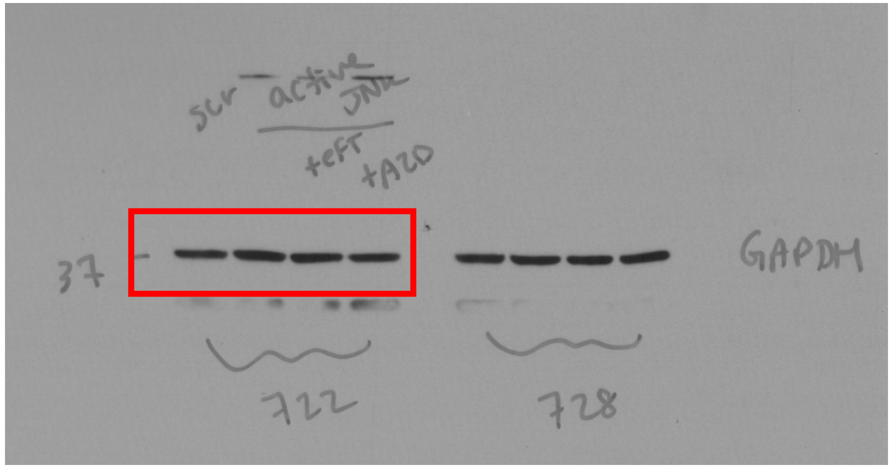

FIGURE 3

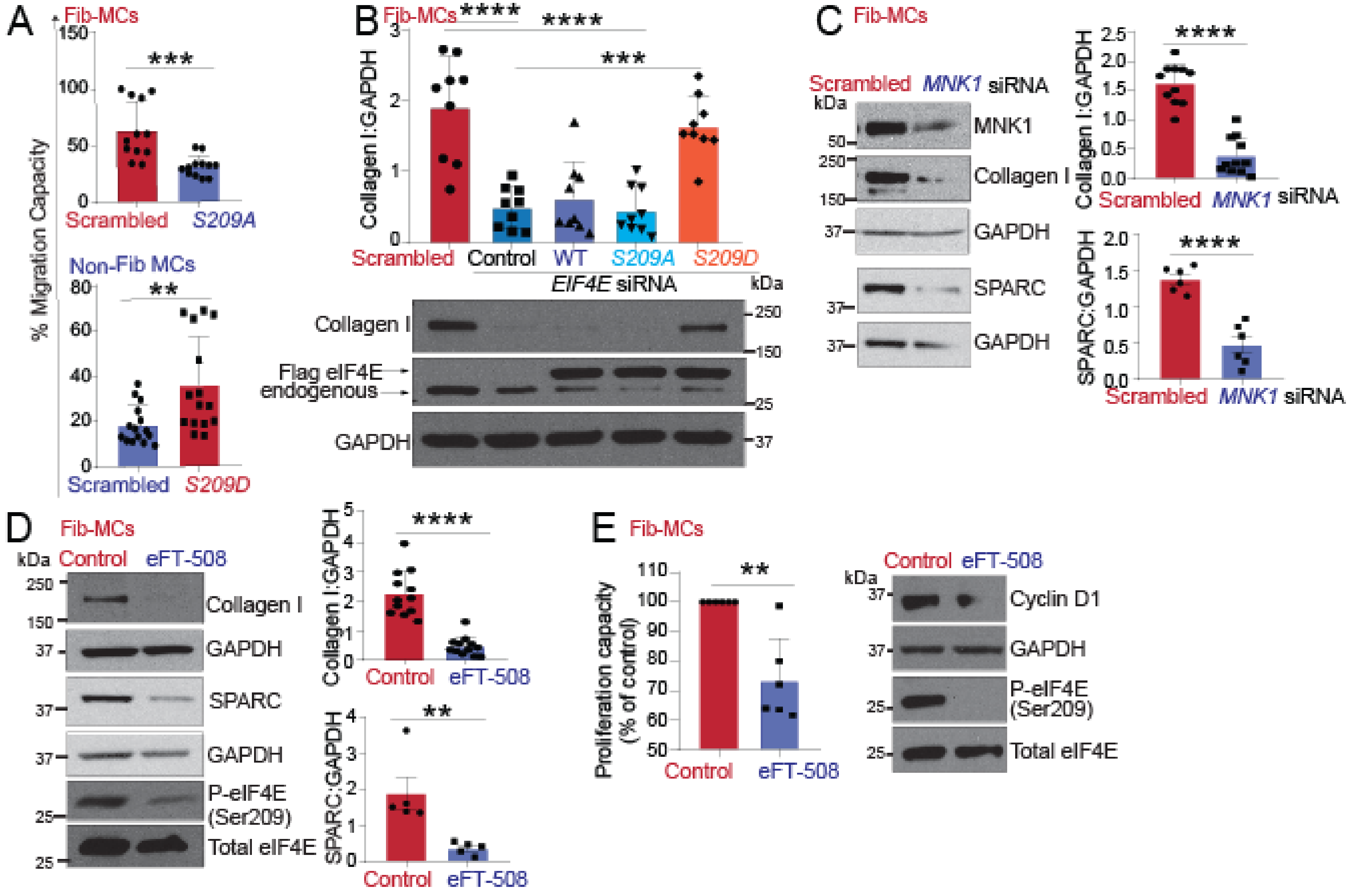

A
Fib-MCs
% Migration Capacity
150
100
50
0
***
Scrambled
S209A
Non-Fib MCs
80
60
40
20
0
**
Scrambled
S209D
B
Fib-MCs
****
****
***
Collagen I:GAPDH
3
2
1
0
Scrambled
Control
WT
S209A
S209D
EIF4E siRNA
kDa
Collagen I
Flag eIF4E
endogenous
GAPDH
250
150
25
37
C
Fib-MCs
Scrambled
MNK1 siRNA
kDa
50
250
150
37
37
37
MNK1
Collagen I
GAPDH
SPARC
GAPDH
Collagen I:GAPDH
2.5
2.0
1.5
1.0
0.5
0.0
****
Scrambled
MNK1 siRNA
SPARC:GAPDH
2.0
1.5
1.0
0.5
0.0
****
Scrambled
MNK1 siRNA
D
Fib-MCs
kDa
Control
eFT-508
250
150
37
37
37
25
25
Collagen I
GAPDH
SPARC
GAPDH
P-eIF4E
(Ser209)
Total eIF4E
Collagen I:GAPDH
5
4
3
2
1
0
****
Control
eFT-508
SPARC:GAPDH
4
3
2
1
0
**
Control
eFT-508
E
Fib-MCs
Proliferation capacity
(% of control)
110
100
90
80
70
60
50
**
Control
eFT-508
kDa
Control
eFT-508
37
37
25
25
Cyclin D1
GAPDH
P-eIF4E
(Ser209)
Total eIF4E

# Figure 3B

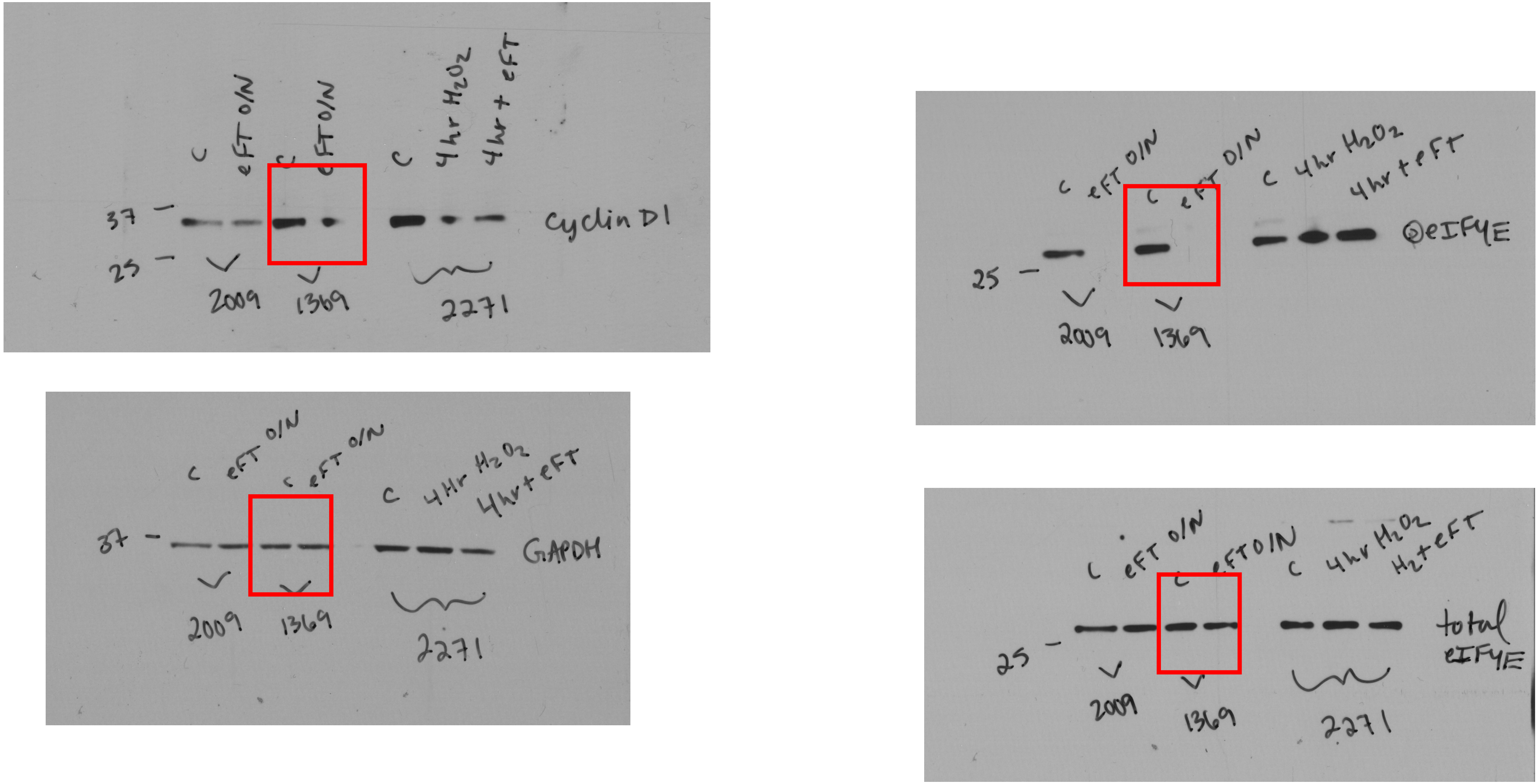

c
eFT O/N
c
eFT O/N
c
4hr H2O2
4hr + eFT
37
25
cyclin D1
2009
1369
2271
37
GAPDH
eIF4E
25
total
eIF4E

# Figure 3C

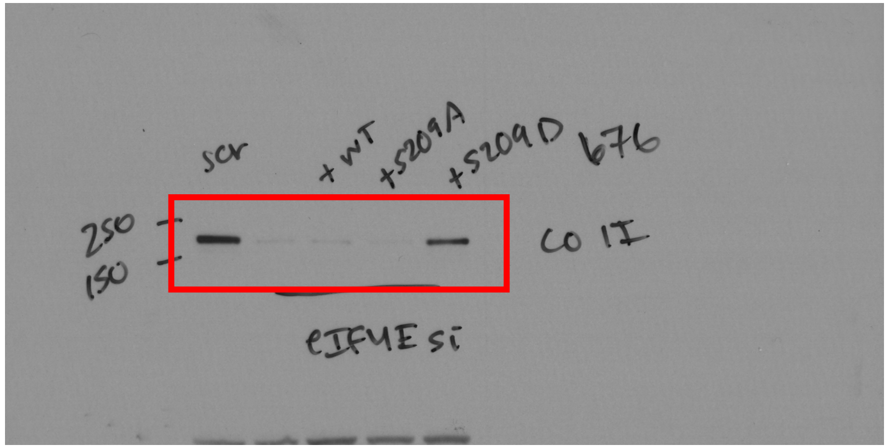

scr
+WT
+S209A
+S209D
676
250
150
Co II
eIF4E si


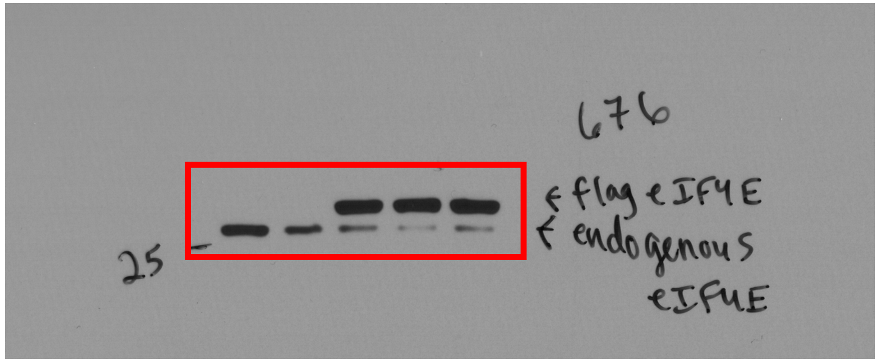

676
← flag eIF4E
← endogenous eIF4E
25


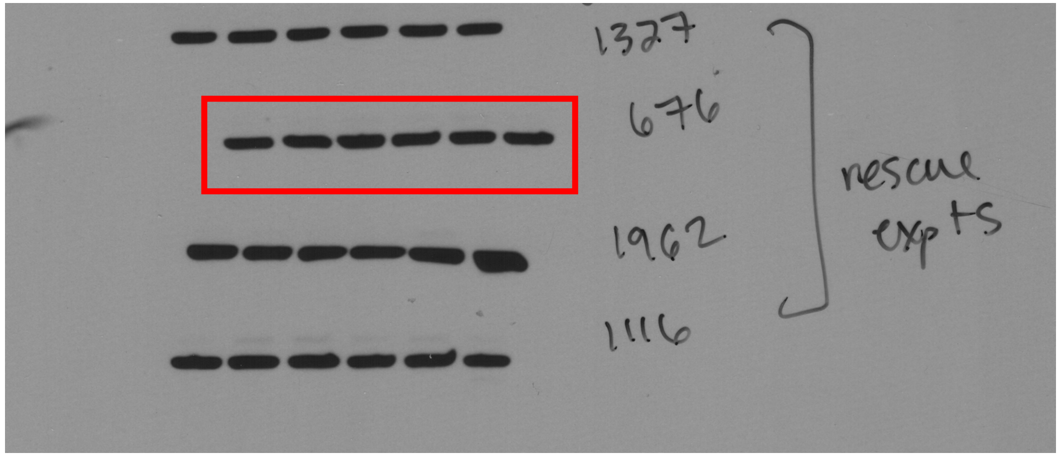

1327
676
1962
1116
rescue expts

# Figure 3D

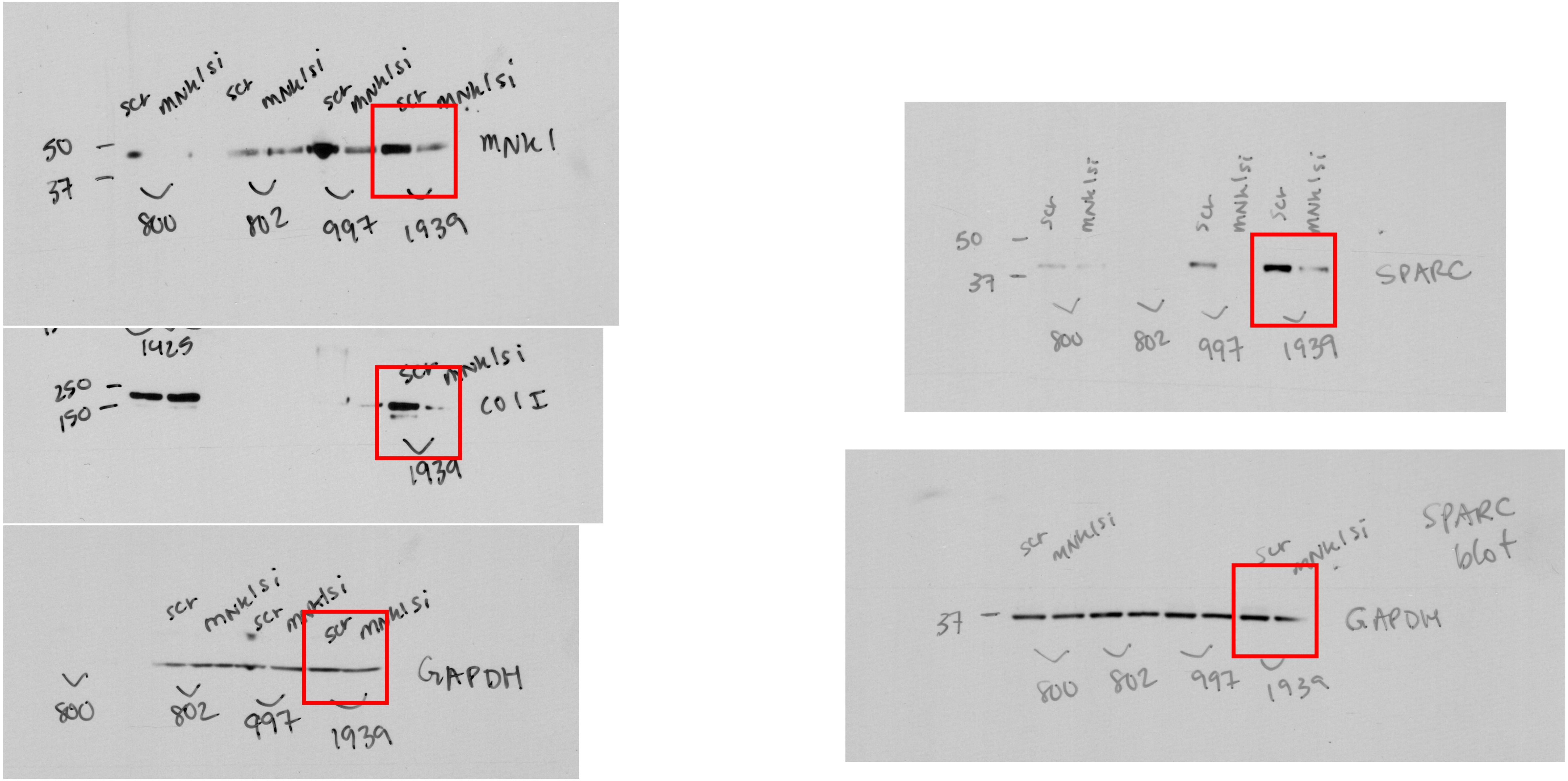
scr
MNK1si
50
37
MNK1
800
802
997
1939
1425
250
150
COL I
GAPDH
SPARC
SPARC blot

# Figure 3E

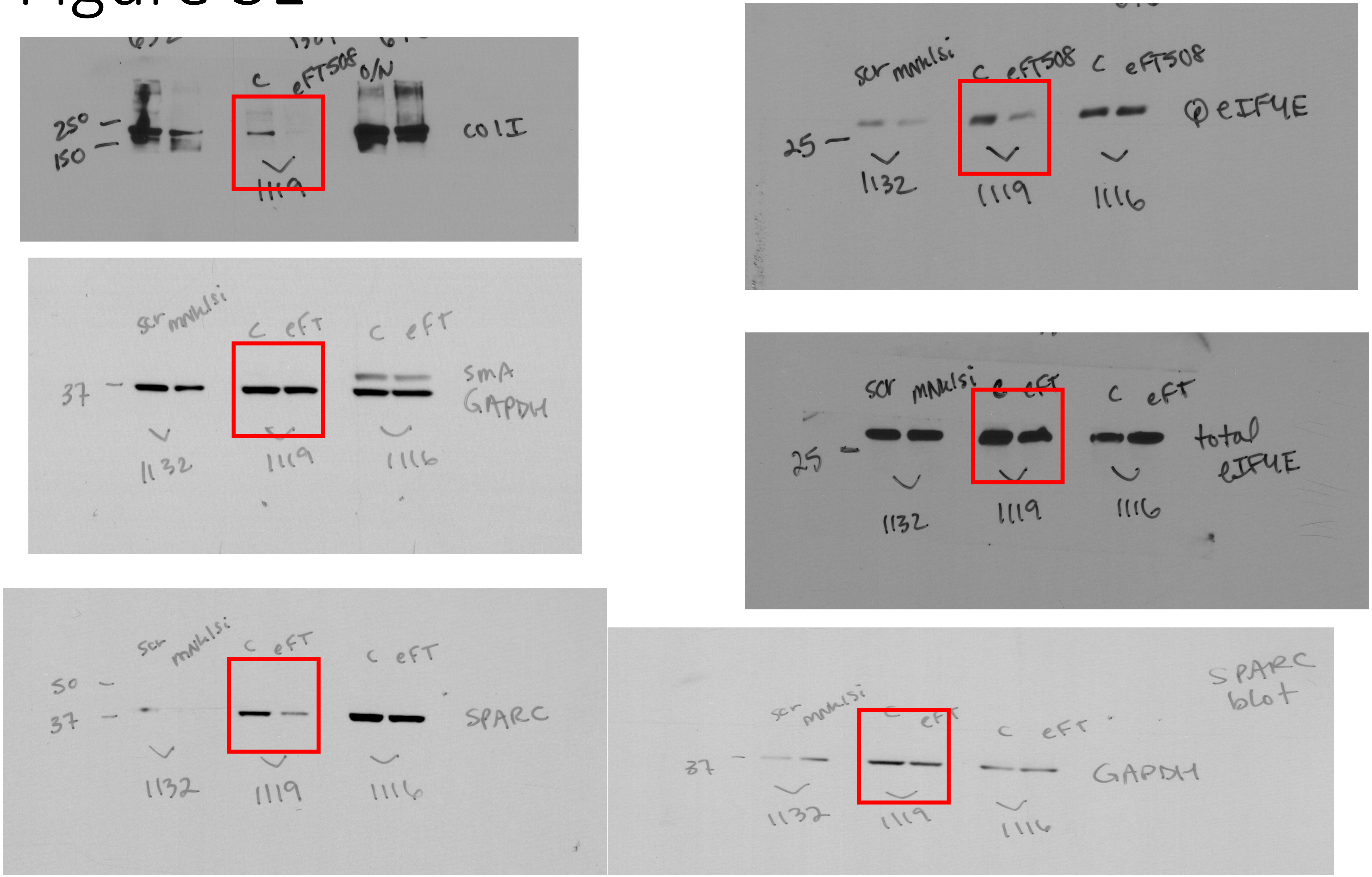

c eFT508
o/N
250
150
1119
col I
scr mnk1si
c eFT508
c eFT508
25
1132
1119
1116
ⓟ eIF4E
c eFT
c eFT
37
SMA
GAPDH
1132
1119
1116
scr mnk1si
c eFT
c eFT
25
total
eIF4E
1132
1119
1116
Scr mnk1si
c eFT
c eFT
50
37
SPARC
1132
1119
1116
SPARC
blot
scr mnk1si
c eFT
c eFT
37
GAPDH
1132
1119
1116

FIGURE 4

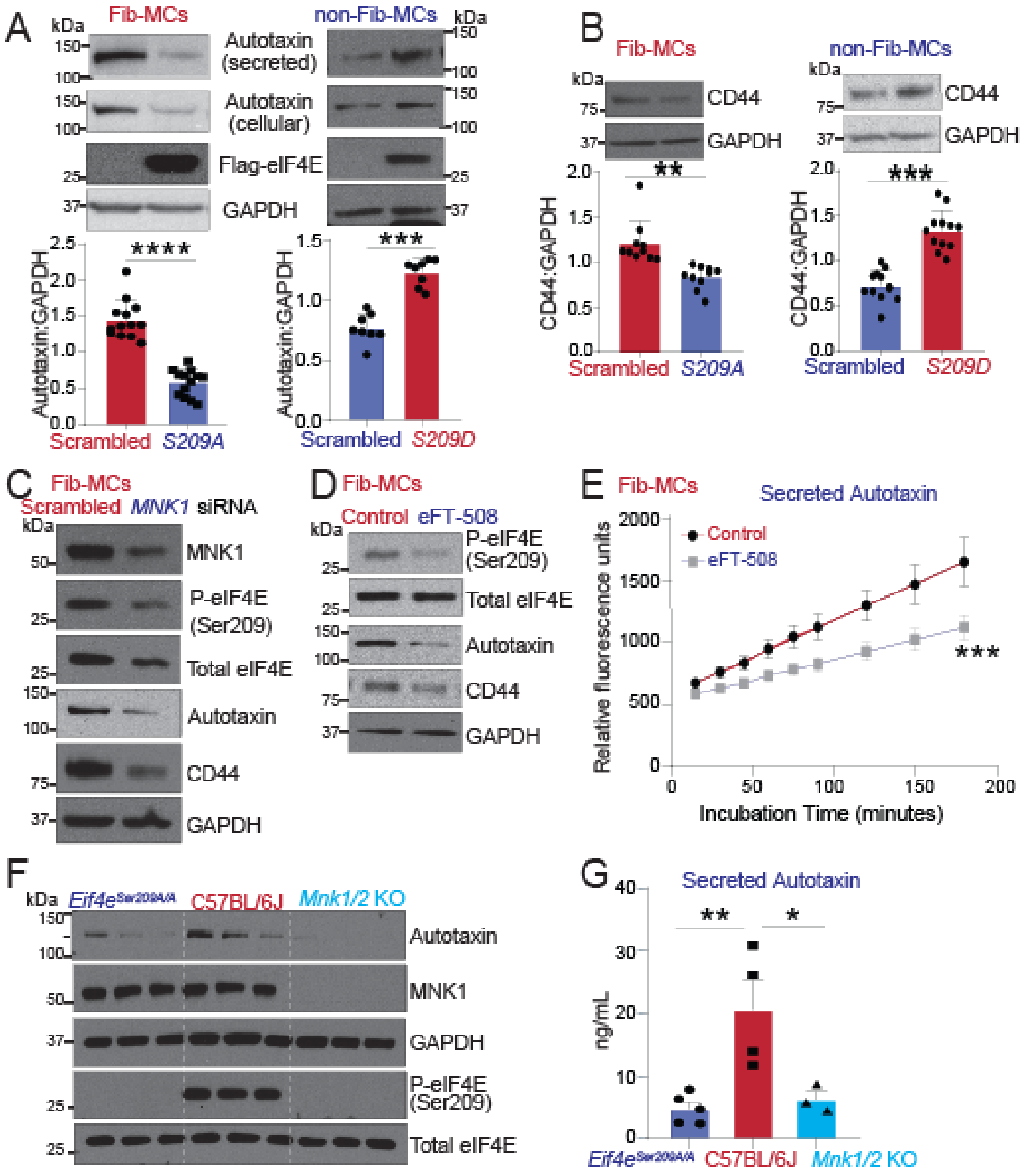

A
Fib-MCs
non-Fib-MCs
kDa
Autotaxin (secreted)
Autotaxin (cellular)
Flag-eIF4E
GAPDH
Autotaxin:GAPDH
****
***
Scrambled
S209A
S209D
B
CD44
GAPDH
CD44:GAPDH
**
***
C
Scrambled MNK1 siRNA
MNK1
P-eIF4E (Ser209)
Total eIF4E
Autotaxin
CD44
D
Control eFT-508
E
Secreted Autotaxin
Control
eFT-508
Relative fluorescence units
Incubation Time (minutes)
F
Eif4e^Ser209A/A
C57BL/6J
Mnk1/2 KO
G
ng/mL

# Figure 4A-S209A blots

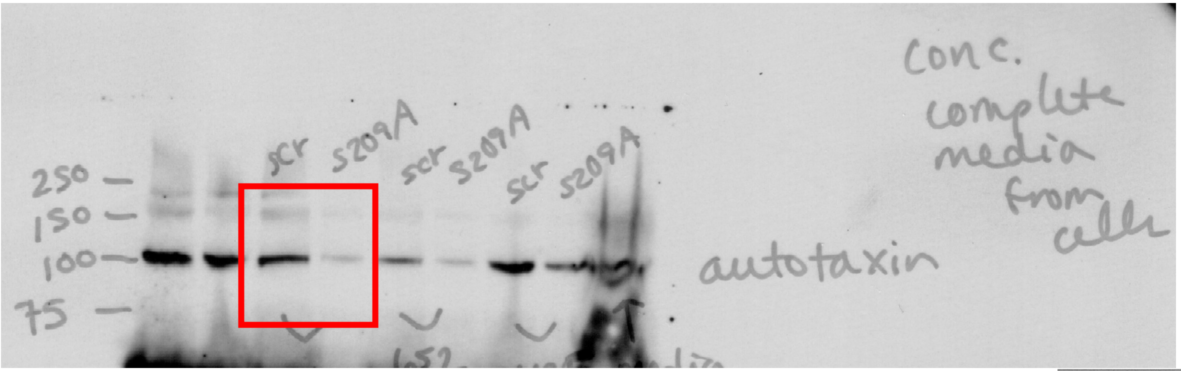


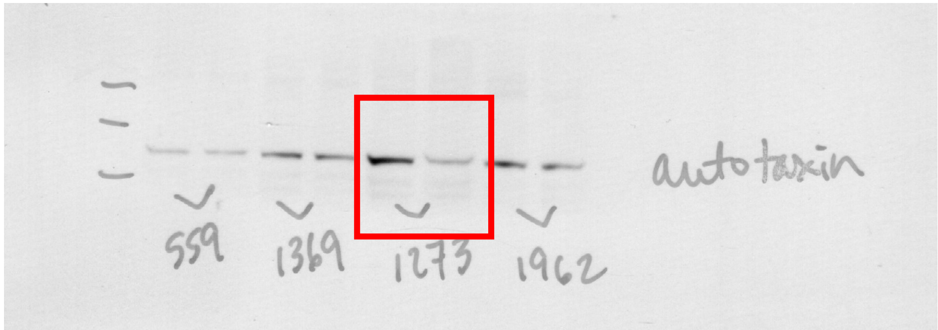


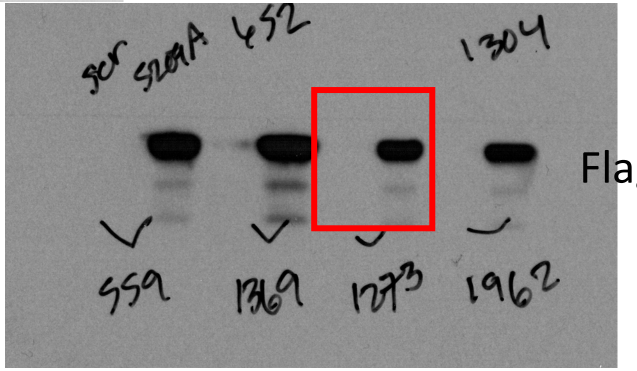


Flag

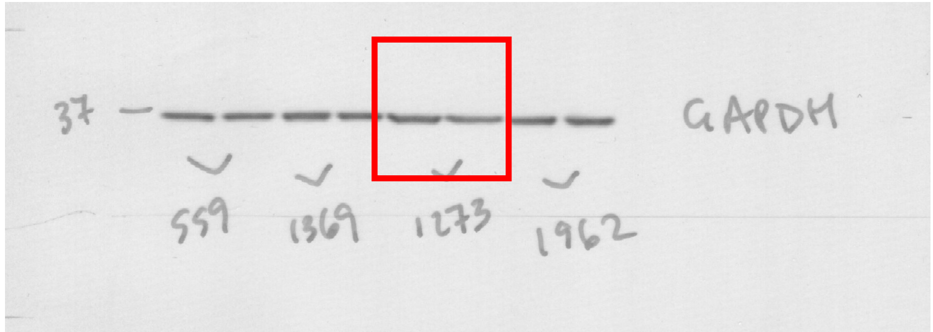

# Figure 4A-S209D blots

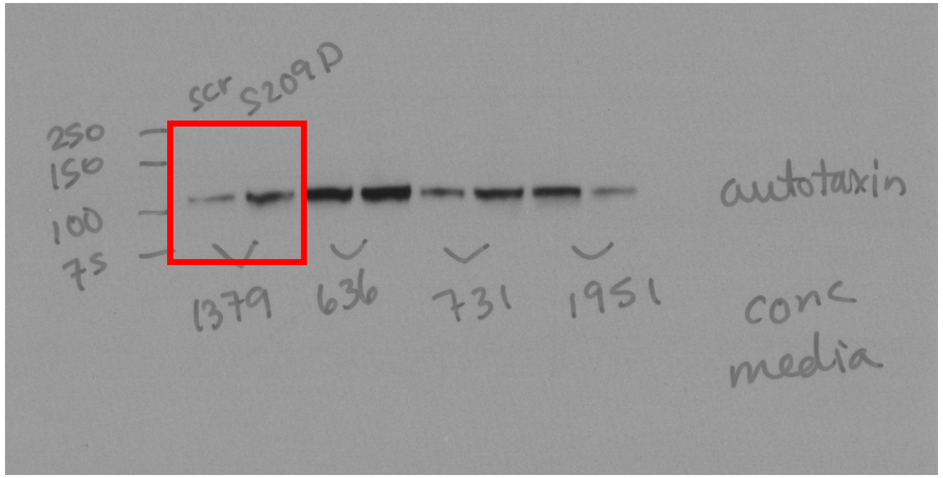


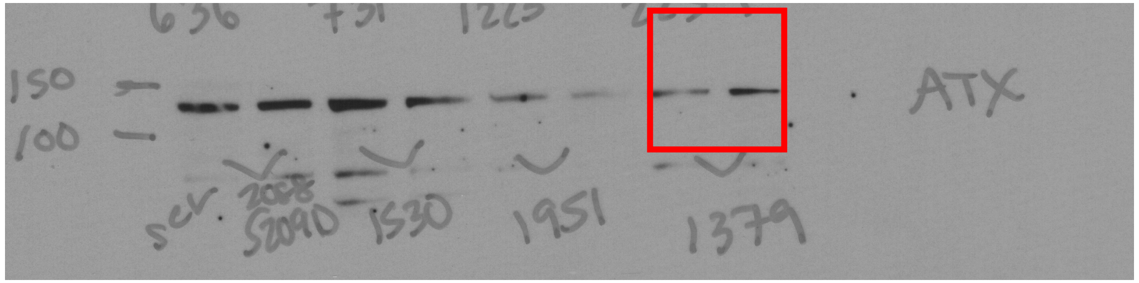


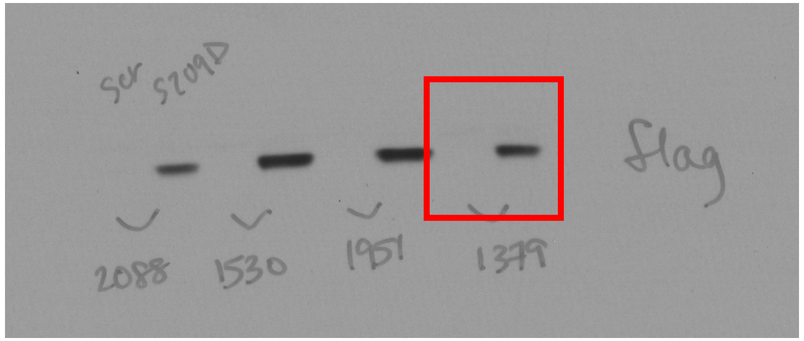


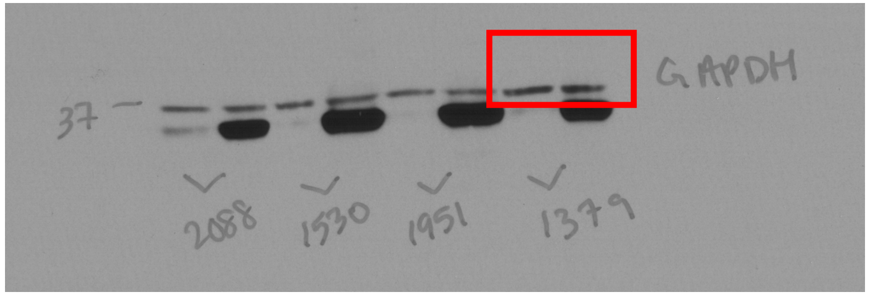

# Figure 4B

S209A figure:

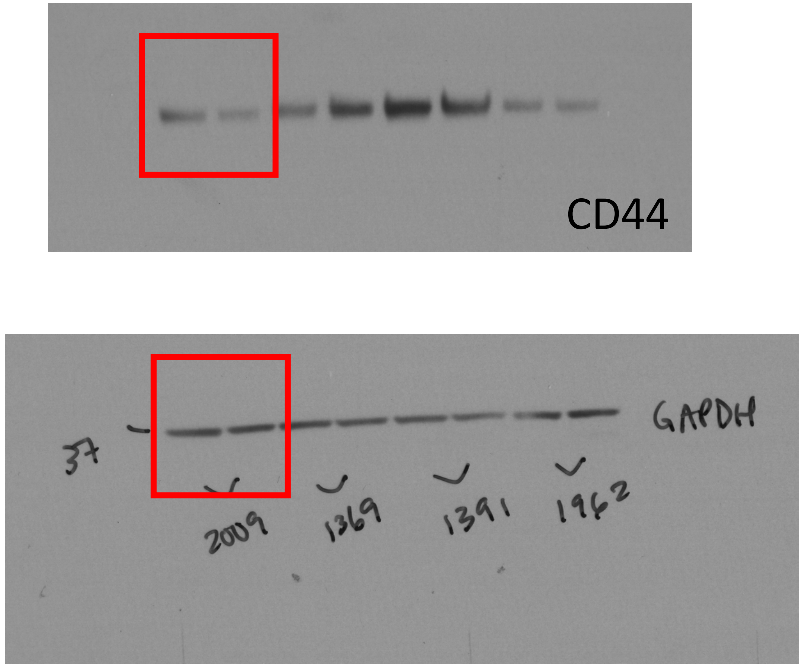


S209D figure:

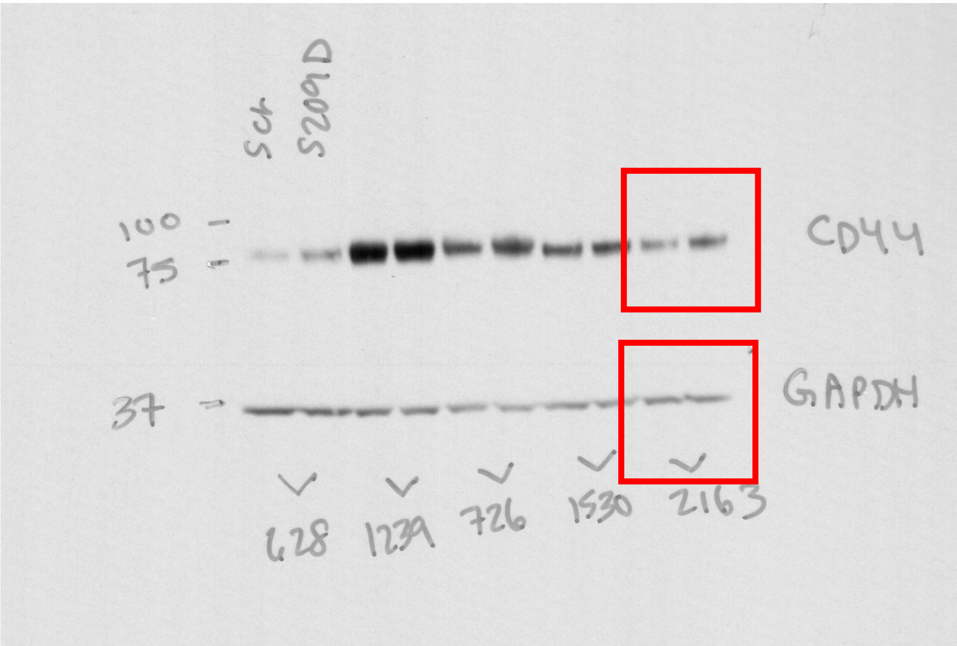

# Figure 4C

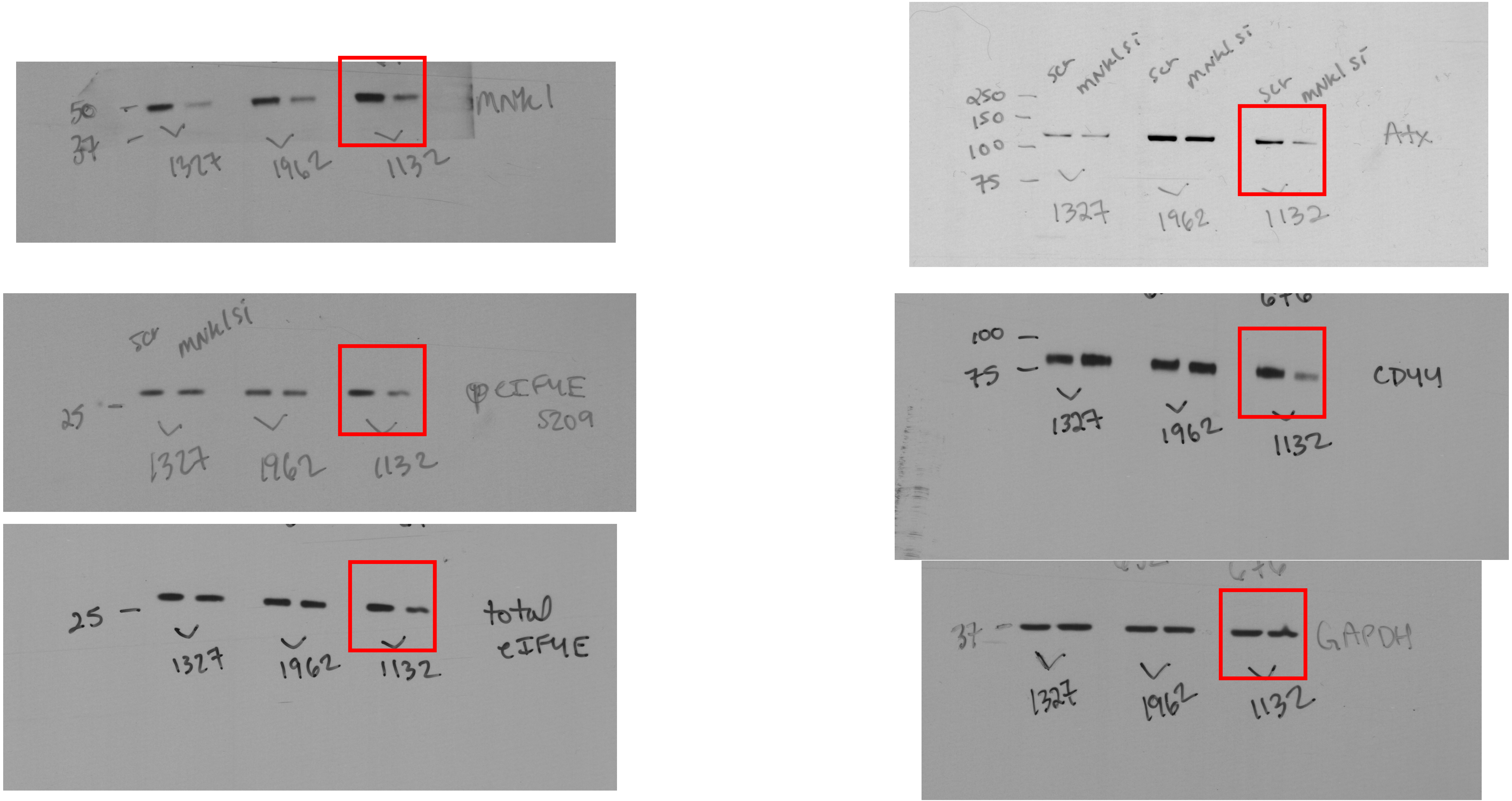

MNK1
1327
1962
1132
scr
mnk1 si
eIF4E S209
total eIF4E
Atx
CD44
GAPDH

# Figure 4D--***P-eIF4E (and maybe total) need fixed/replaced in final figure

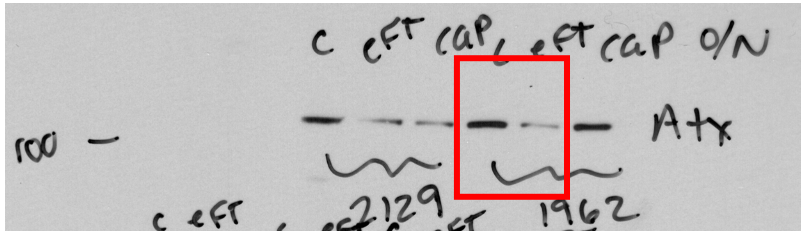


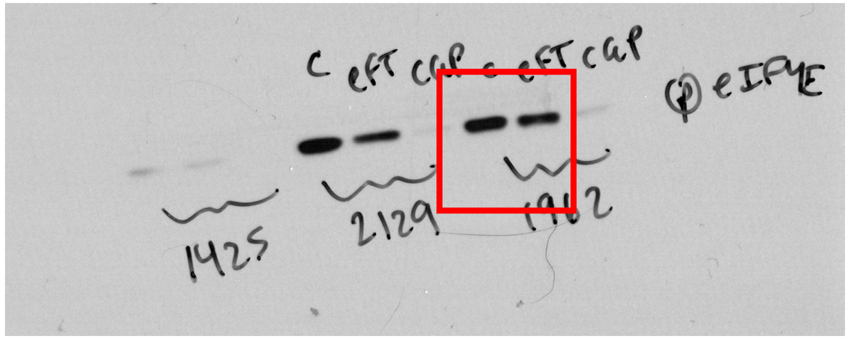


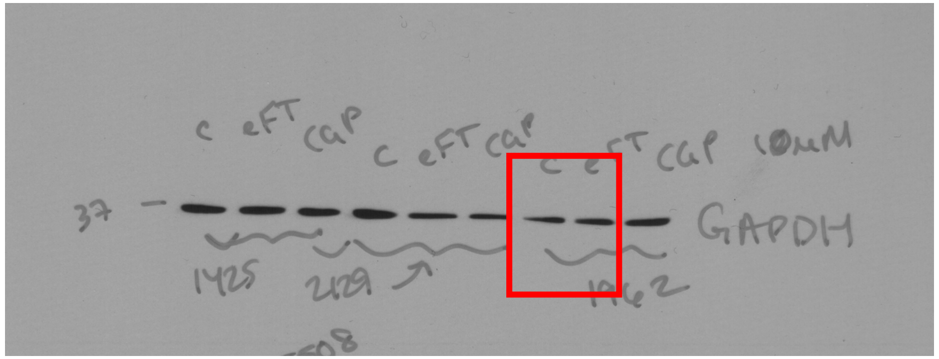


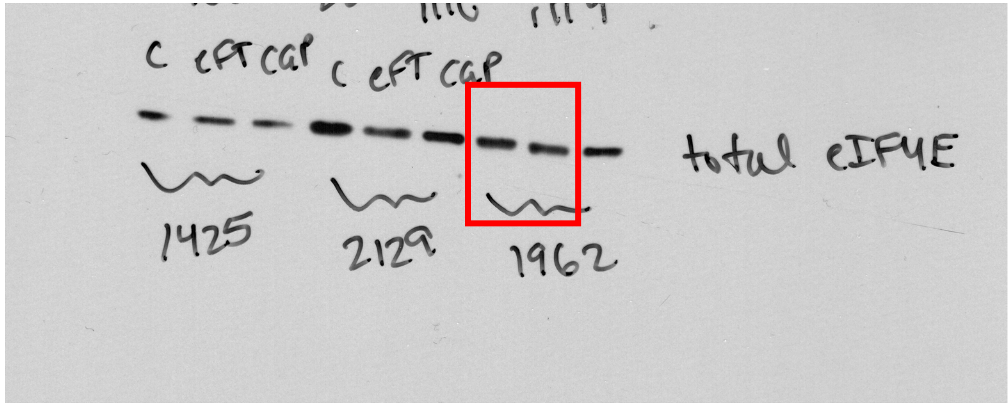


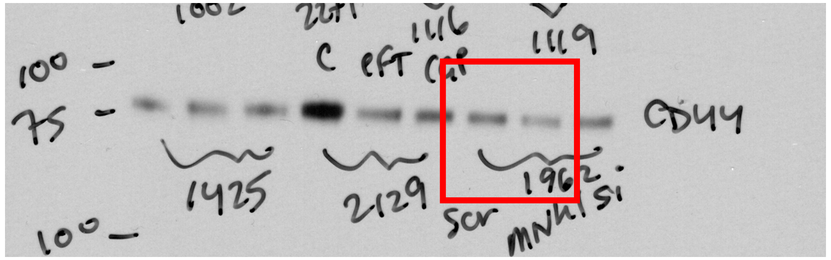

# Figure 4F

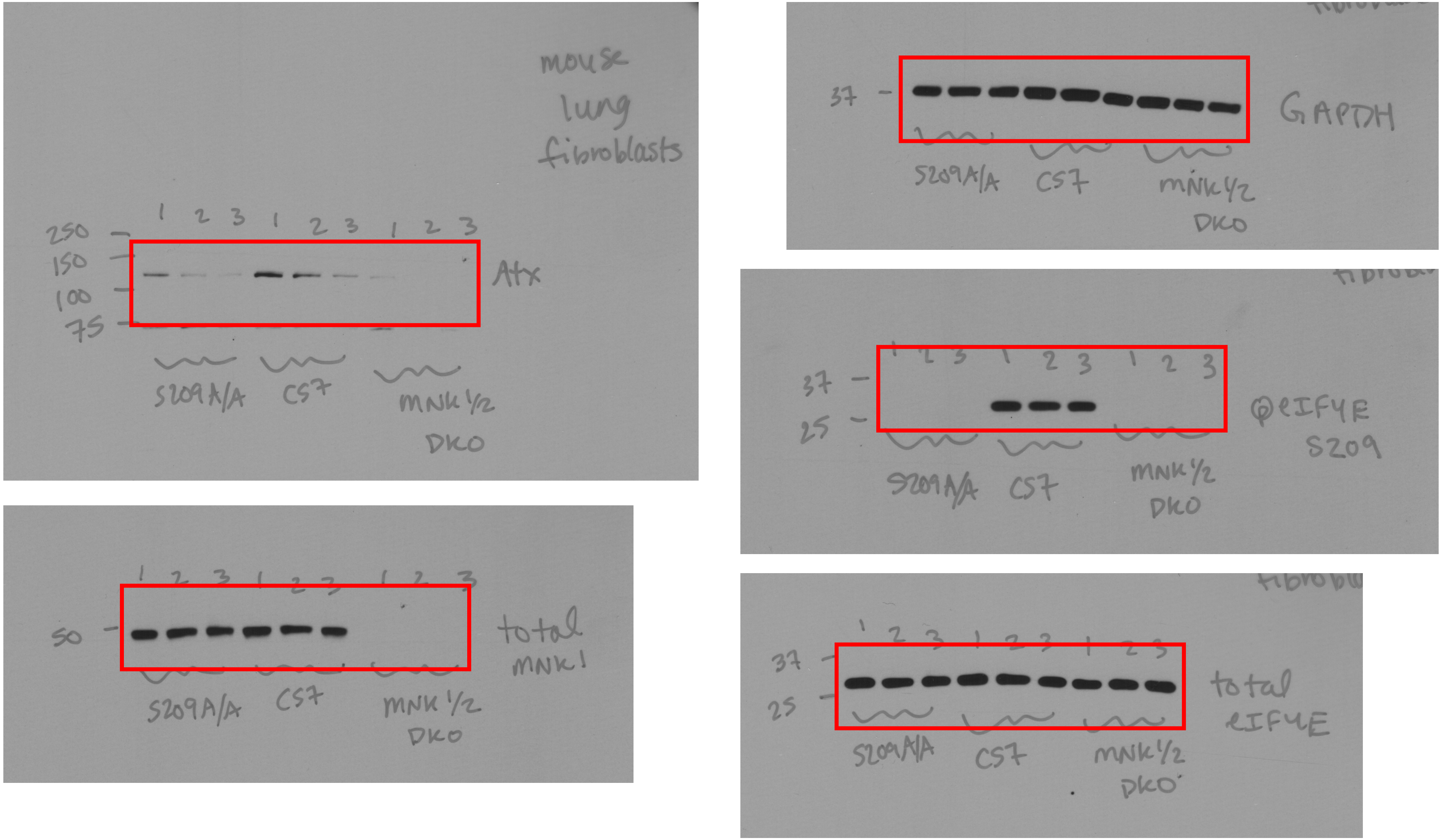

mouse lung fibroblasts
Atx
250
150
100
75
S209A/A
C57
MNK1/2 DKO
GAPDH
37
pEIF4E S209
25
total MNK1
50
total eIF4E

FIGURE 5

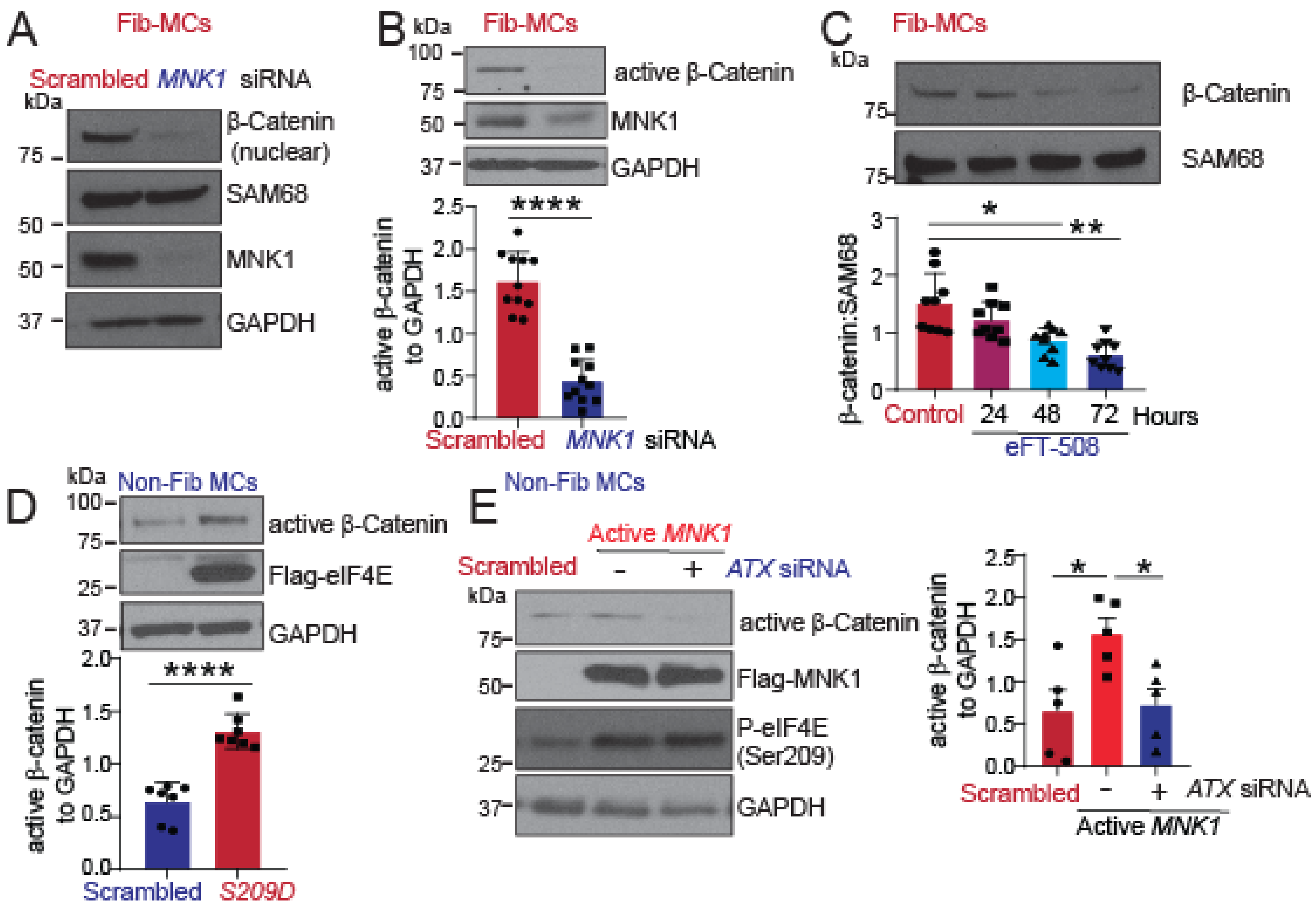

A
Fib-MCs
Scrambled MNK1 siRNA
kDa
75
50
50
37
β-Catenin (nuclear)
SAM68
MNK1
GAPDH
B
kDa
Fib-MCs
100
75
50
37
active β-Catenin
MNK1
GAPDH
****
active β-catenin to GAPDH
2.5
2.0
1.5
1.0
0.5
0.0
Scrambled MNK1 siRNA
C
Fib-MCs
kDa
75
75
β-Catenin
SAM68
*
**
β-catenin:SAM68
3
2
1
0
Control 24 48 72 Hours
eFT-508
D
kDa
Non-Fib MCs
100
75
25
37
active β-Catenin
Flag-eIF4E
GAPDH
****
active β-catenin to GAPDH
2.0
1.5
1.0
0.5
0.0
Scrambled S209D
E
Non-Fib MCs
Active MNK1
Scrambled - + ATX siRNA
kDa
75
50
25
37
active β-Catenin
Flag-MNK1
P-eIF4E (Ser209)
GAPDH
* *
active β-catenin to GAPDH
2.5
2.0
1.5
1.0
0.5
0.0
Scrambled - + ATX siRNA
Active MNK1

# Figure 5A

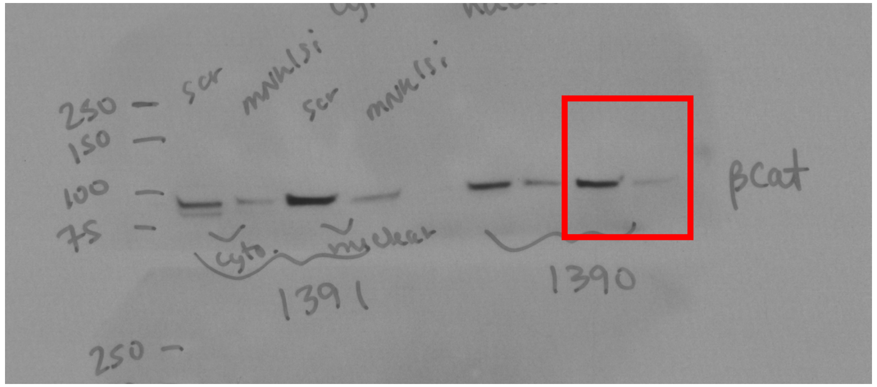
250
150
100
75
scr
mnk1si
scr
mnk1si
βcat
cyto
nuclear
1391
1390
250

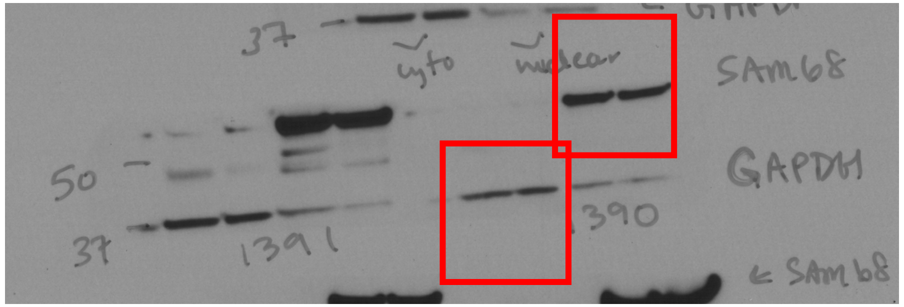
37
cyto
nuclear
SAm68
GAPDH
50
37
1391
1390
← SAm68

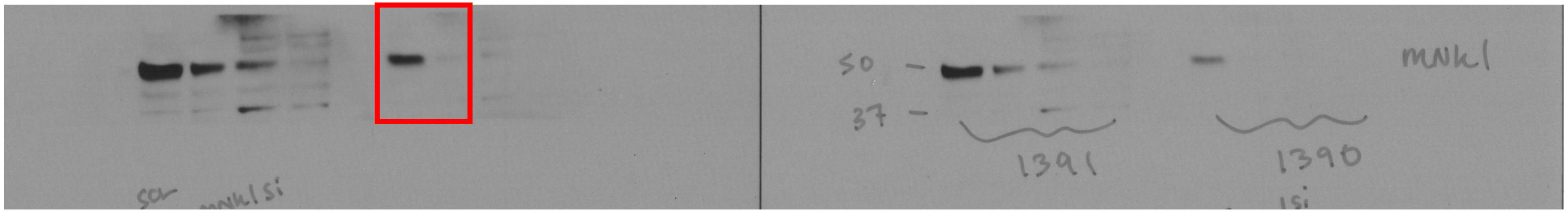
50
37
mnk1
1391
1390
scr
mnk1si

# Figure 5B

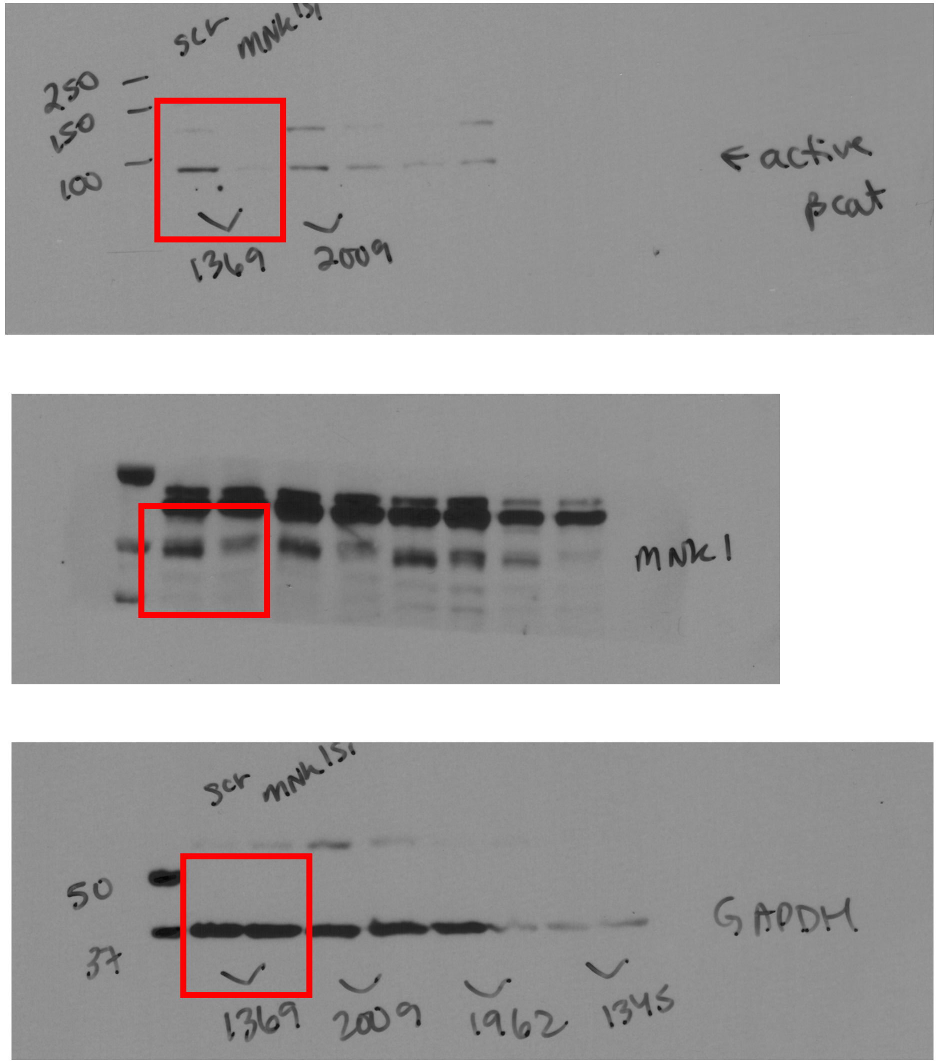
scr
MNK1si
250
150
100
1369
2009
← active βcat
MNK1
scr
MNK1si
50
37
GAPDH
1369
2009
1962
1345

# Figure 5C

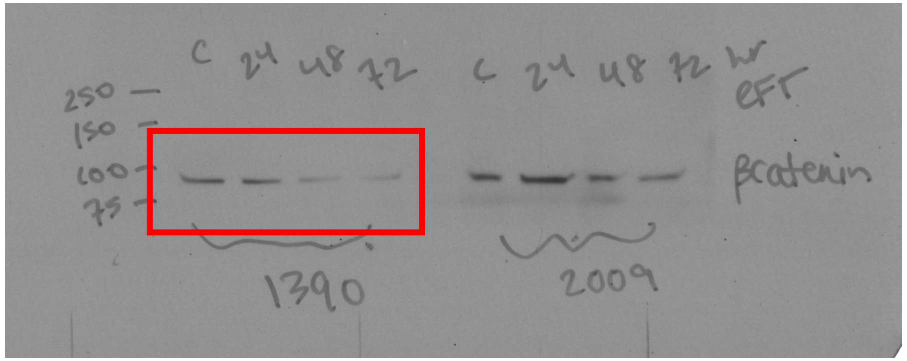

250
150
100
75
C 24 48 72
C 24 48 72 hr
eFT
βcatenin
1390
2009


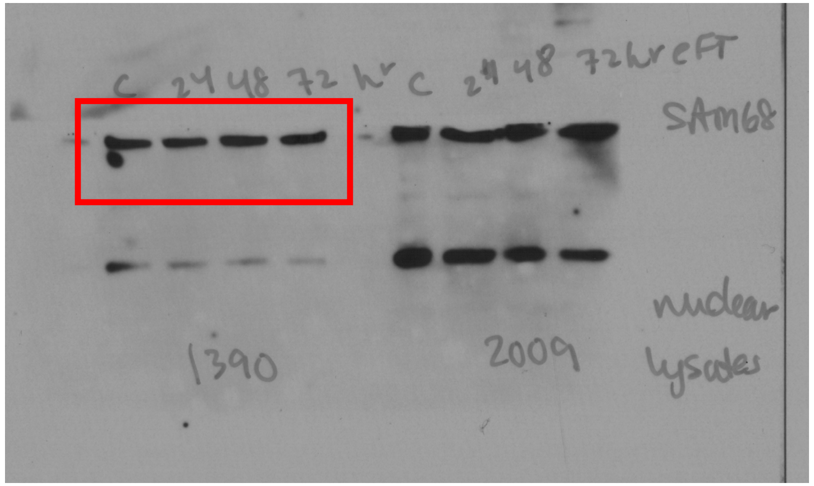

C 24 48 72 hr C 24 48 72 hr eFT
SAM68
nuclear
lysates
1390
2009

# Figure 5D

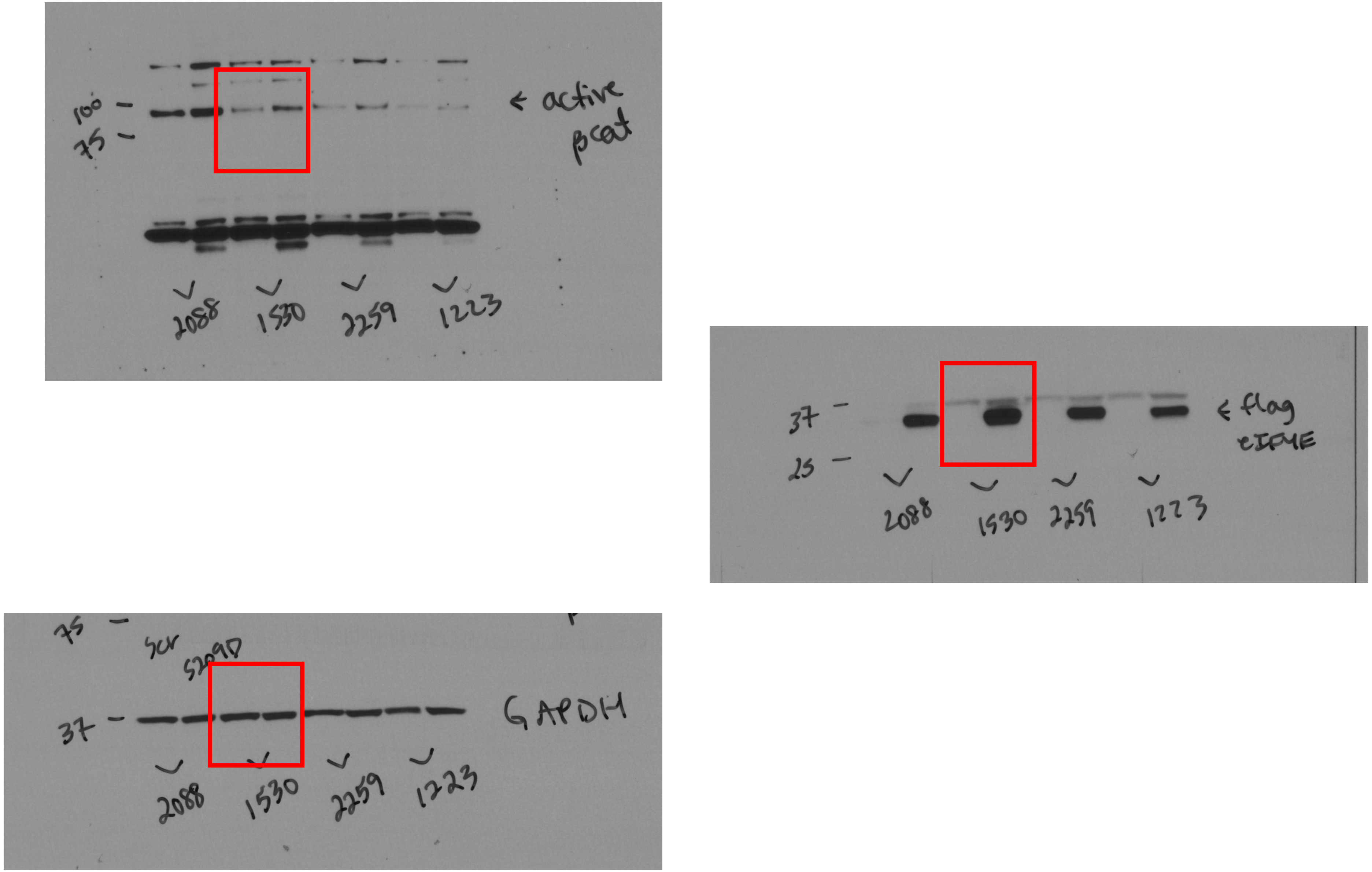
100
75
active βcat
2088
1530
2259
1223
37
25
flag eIF4E
2088
1530
2259
1223
75
scr
S209D
37
GAPDH
2088
1530
2259
1223

# Figure 5E

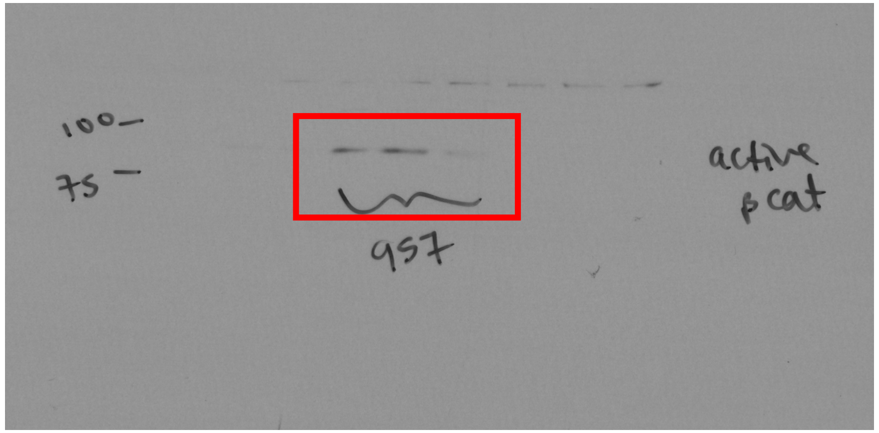
100
75
957
active
β cat

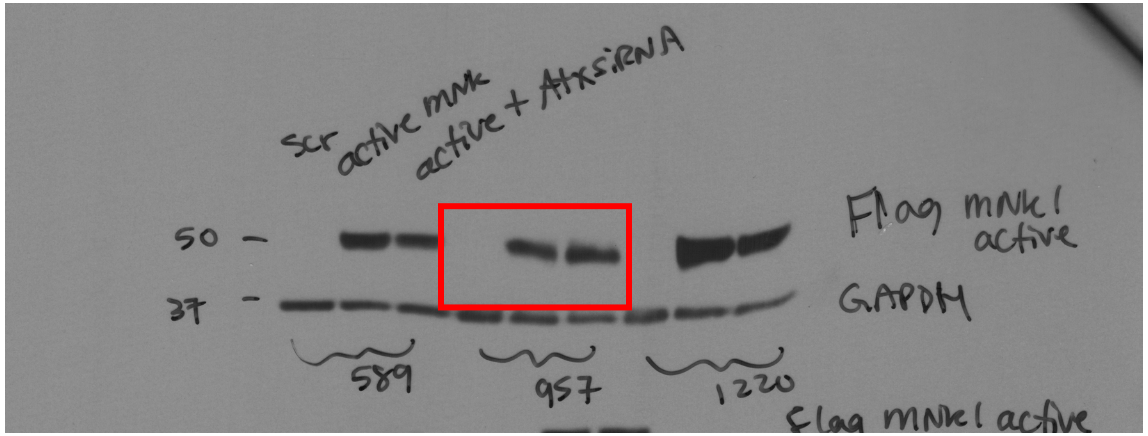
scr
active mnk
active + Atx siRNA
50
37
Flag mnk1
active
GAPDH
589
957
1220
Flag mnk1 active

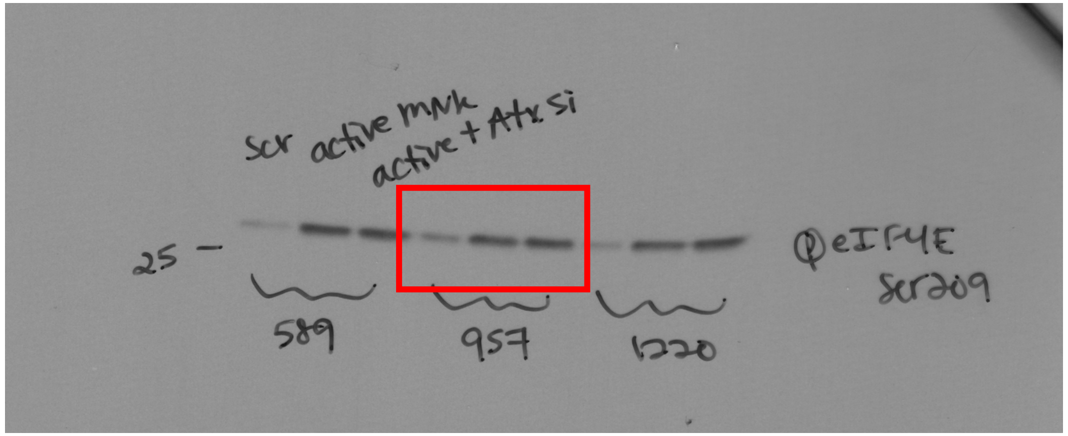
scr
active mnk
active + Atx si
25
ΦeIF4E
Ser209
589
957
1220

FIGURE 7

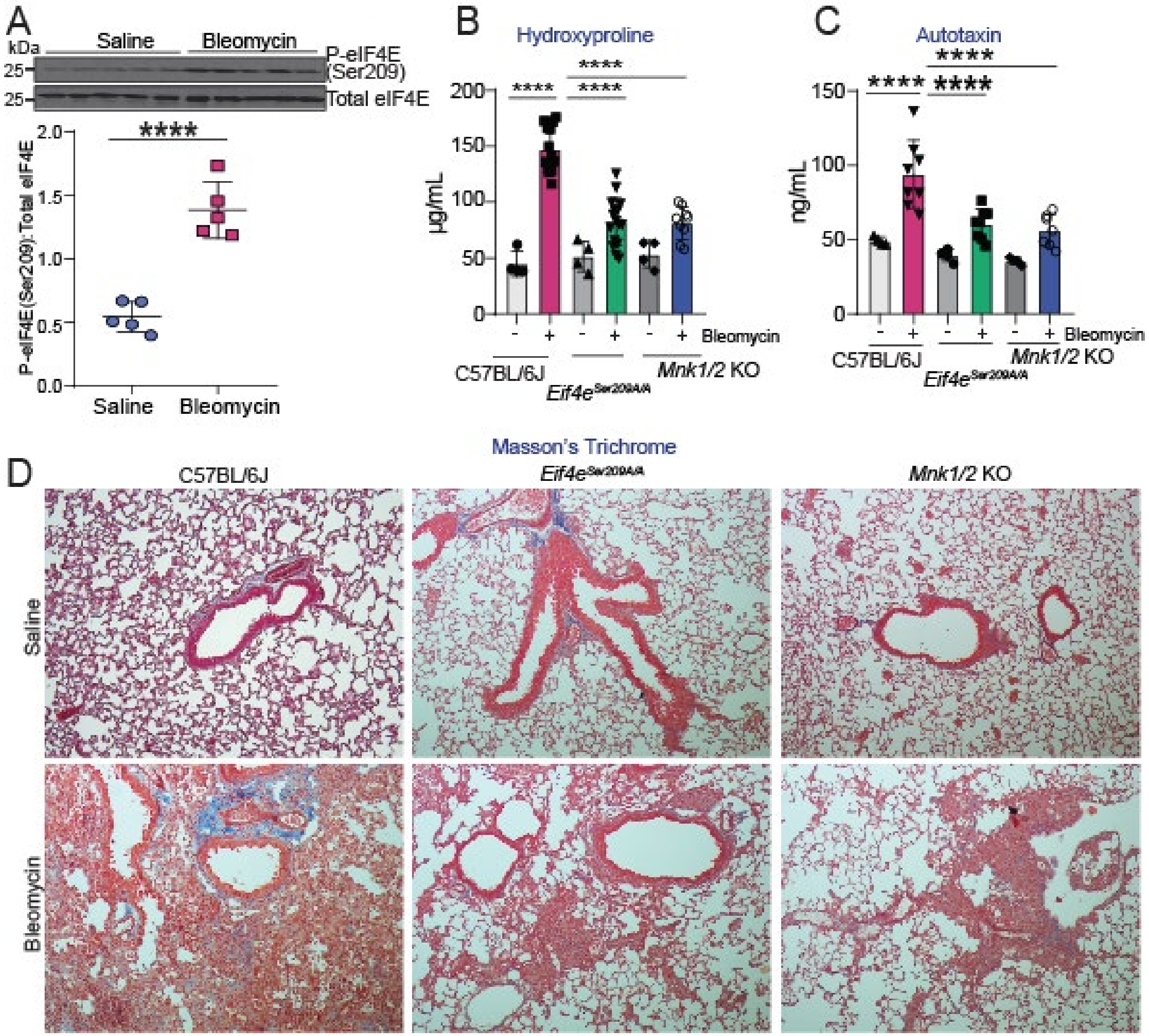

A
kDa
Saline
Bleomycin
P-eIF4E (Ser209)
Total eIF4E
25
25
P-eIF4E(Ser209): Total eIF4E
****
Saline
Bleomycin
B
Hydroxyproline
µg/mL
****
Bleomycin
C57BL/6J
Eif4e Ser209A/A
Mnk1/2 KO
C
Autotaxin
ng/mL
D
Masson's Trichrome
C57BL/6J
Eif4e Ser209A/A
Mnk1/2 KO
Saline
Bleomycin

# Figure 7A

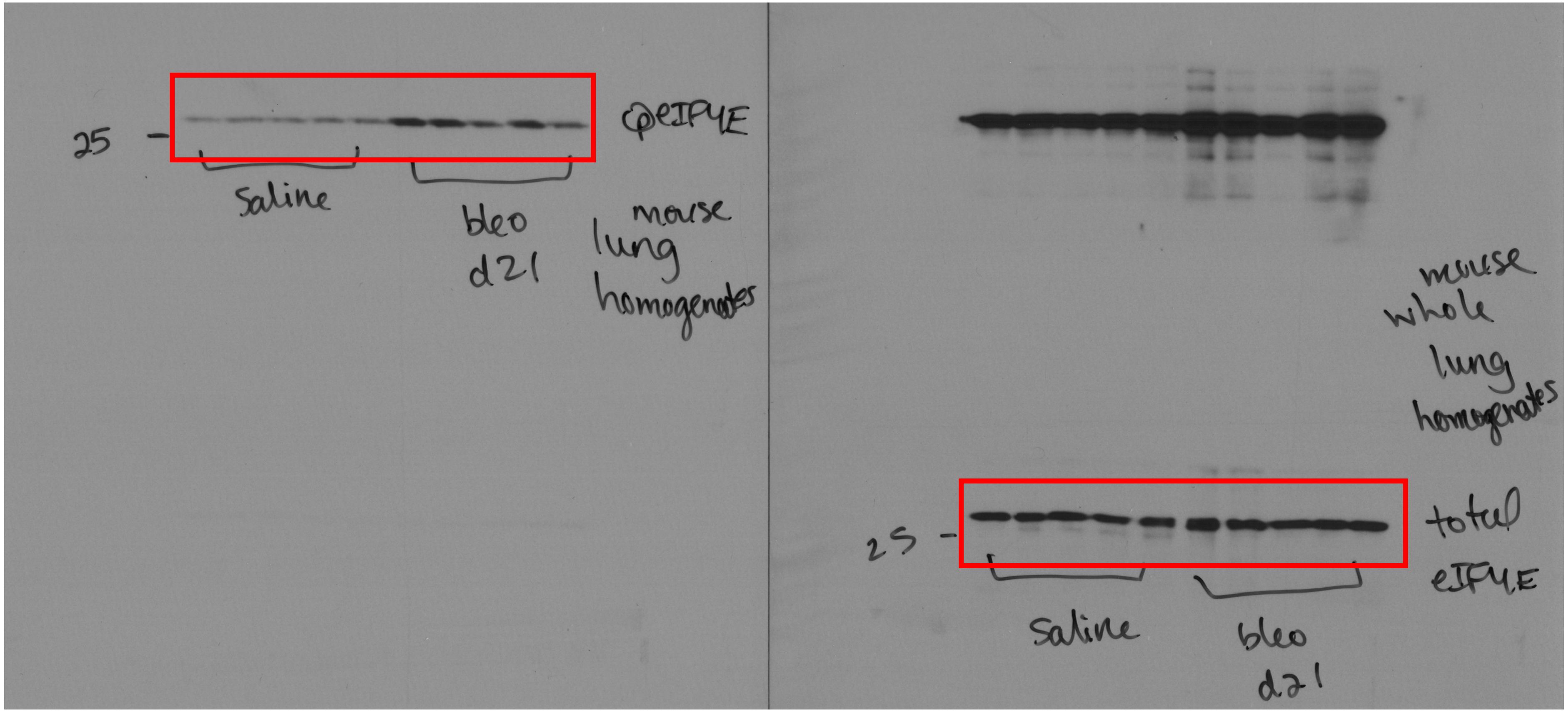
25
peIF4E
saline
bleo
d21
mouse
lung
homogenates
mouse
whole
lung
homogenates
25
total
eIF4E
saline
bleo
d21
